# Individual-based simulation of the spatial and temporal dynamics of macroinvertebrate functional groups provides insights into benthic community assembly mechanisms

**DOI:** 10.7717/peerj.5038

**Published:** 2018-06-18

**Authors:** Nikolaos Alexandridis, Cédric Bacher, Nicolas Desroy, Fred Jean

**Affiliations:** 1 DYNECO-LEBCO, IFREMER, Centre de Bretagne, Plouzané, France; 2 Laboratoire Environnement et Ressources de Bretagne Nord, IFREMER, Station CRESCO, Dinard, France; 3 LEMAR, Institut Universitaire Européen de la Mer, Université de Brest, UBO, CNRS, IRD, Plouzané, France

**Keywords:** Individual-based model, Inter-scale modeling, Functional groups, Benthic macroinvertebrates, Community assembly, Biological traits, Biotic interactions, α-diversity, β-diversity, Rance estuary

## Abstract

The complexity and scales of the processes that shape communities of marine benthic macroinvertebrates has limited our understanding of their assembly mechanisms and the potential to make projections of their spatial and temporal dynamics. Individual-based models can shed light on community assembly mechanisms, by allowing observed spatiotemporal patterns to emerge from first principles about the modeled organisms. Previous work in the Rance estuary (Brittany, France) revealed the principal functional components of its benthic macroinvertebrate communities and derived a set of functional relationships between them. These elements were combined here for the development of a dynamic and spatially explicit model that operates at two spatial scales. At the fine scale, modeling each individual’s life cycle allowed the representation of recruitment, inter- and intra-group competition, biogenic habitat modification and predation mortality. Larval dispersal and environmental filtering due to the tidal characteristics of the Rance estuary were represented at the coarse scale. The two scales were dynamically linked and the model was parameterized on the basis of theoretical expectations and expert knowledge. The model was able to reproduce some patterns of α- and β-diversity that were observed in the Rance estuary in 1995. Model analysis demonstrated the role of local and regional processes, particularly early post-settlement mortality and spatially restricted dispersal, in shaping marine benthos. It also indicated biogenic habitat modification as a promising area for future research. The combination of this mechanism with different substrate types, along with the representation of physical disturbances and more trophic categories, could increase the model’s realism. The precise parameterization and validation of the model is expected to extend its scope from the exploration of community assembly mechanisms to the formulation of predictions about the responses of community structure and functioning to environmental change.

## Introduction

Environmental change appears to adversely affect ecosystem functioning, both directly and through its impact on biodiversity (Hooper et al., 2012). This indirect effect gains importance in the face of expected high biodiversity losses (Bellard et al., 2012). The accuracy and precision of projections of biodiversity responses to environmental change depend on uncertainty regarding environmental change itself and the reliability of predictions of biodiversity responses to it. Predictions that are primarily based on observed correlations among ecosystem components are of limited reliability in novel or non-equilibrium contexts (Kearney & Porter, 2009). The reliability of predictions can be increased through the representation of community assembly mechanisms (Gotelli et al., 2009). However, mechanistic understanding of communities is often difficult to generate empirically, due to the complexity of ecological processes and the spatial and temporal scales at which they operate (Cardinale et al., 2012).

The formulation of mechanistic models of community assembly offers an alternative way to understand the processes that drive population dynamics (Amarasekare, 2003). Models based on mathematical forms that describe changes in the distribution functions of a community’s populations have the advantage of analytical tractability. Extensions to this modeling approach, such as structured populations (Tuljapurkar & Caswell, 1997) and metapopulations (Hanski, 1998), have widened the range of community assembly mechanisms that can be represented. Still, the amount of knowledge that is required for the formulation and parameterization of mathematical expressions has restricted community models to the representation of mostly trophic biotic interactions (but see Kéfi et al. (2012)).

Alternatively, simulation models that describe populations by keeping track of each individual, also known as individual-based models (IBMs), are particularly well-suited to the representation of diverse biotic interactions. This is due to their ability to model individual variation, including in the way organisms interact, in greater detail than mathematical formulations (DeAngelis & Mooij, 2005). In IBMs, complex patterns largely emerge from simple rules applied at the individual level, instead of being imposed by the modeler. Rules rooted in energetic or evolutionary theory offer higher model robustness, but community theory and empirical understanding can also form the basis for IBMs, provided that demographic rates emerge from the individuals’ behavior (Grimm & Berger, 2016). The comparison of model output with biodiversity observations allows the assessment of the community theories that initially dictated the model’s rules (Evans et al., 2013).

The development of IBMs can be particularly useful for initial understanding, and ultimately, predicting the patterns of communities of marine benthic macro- (i.e., visible with the naked eye) invertebrates. First, the potential to reproduce the dynamics of these systems has been very limited, in large part due to the challenge of allowing observed community patterns to emerge from available empirical knowledge about the interactions of individual organisms (Constable, 1999). Second, traditional non-spatial models often assume that the strengths of species interactions are proportional to their abundances, ignoring the key role of local processes in marine benthos (Dunstan & Johnson, 2005). IBMs can address both of these issues, by modeling spatially explicit processes at the level of individuals, placing emphasis on the emergence of patterns at higher levels of biological organization (Grimm & Berger, 2016).

IBMs of marine benthic communities have been mostly developed for hard bottom-dwelling organisms. For instance, Dunstan & Johnson (2005) reproduced the natural variability of macroinvertebrate epibenthic communities, based on empirical estimates of intra- and inter-specific demographic rates applied at the individual and colony level. In a similar example from a system where IBM applications abound, Wakeford, Done & Johnson (2008) used empirically derived rates to model competitive interactions within a coral community and investigate the influence of disturbance regimes. Mumby et al. (2006) included the interaction of corals, macroalgae and their invertebrate and fish grazers in one of the most comprehensive benthic community IBMs. They were thus able to investigate the system’s functioning and formulate clear management recommendations. Yñiguez, McManus & DeAngelis (2008), on the other hand, focused only on the dominant macroalgae of a coral reef community and represented their spread and growth in three dimensions to assess the role of key environmental conditions.

The definition of entities is a major challenge for models of biodiversity (Queirós et al., 2015). Benthic community IBMs have addressed this challenge through two main approaches. Some modelers, including Dunstan & Johnson (2005), opt for the representation of a community’s dominant species, whether that be in terms of density or biomass. This approach has the advantage of a clear interpretation of model entities and a straightforward assignment of biological attributes. The other approach, selected by Wakeford, Done & Johnson (2008), defines functional groups by which the majority of species can be represented. This approach offers a more comprehensive community representation, while the two can also be combined (see Mumby et al. (2006)). Both approaches tend to ignore the functional contribution of rare species (Lyons et al., 2005), while grouping of organisms and assignment of attributes can be highly subjective. There also appears to be a trade-off between the richness of modeled entities and mechanisms. For instance, the mechanistically-rich model of Mumby et al. (2006) does not reach the number of entities contained in the work of Wakeford, Done & Johnson (2008), but is well ahead of the even more mechanistically-rich model of Yñiguez, McManus & DeAngelis (2008). Furthermore, even the most comprehensive community representations are constrained by observation and, therefore, prone to fail in highly diverse areas and non-equilibrium contexts.

The lack of a holistic, systematic and testable approach to the definition of IBM entities led Boulangeat et al. (2012) to develop their own framework. Their scheme allows the classification of terrestrial vegetation into groups that are representative of plant biodiversity and consistent with the parameters and processes of dynamic models (Boulangeat, Georges & Thuiller, 2014). The parameter values assigned to members of these groups should depend on the mechanistic resolution of the modeled processes. For instance, Dunstan & Johnson (2005) modeled the transition of the occupation of substrate patches from competitively inferior to competitively superior species, lumping together the various processes that are responsible for this transition. This technique facilitated the model’s parameterization through the estimation of competition outcomes from repeated observations of pair-wise species interactions. On the other hand, Yñiguez, McManus & DeAngelis (2008) modeled the growth and reproduction of individual organisms in response to their environment, allowing species interactions and demographics to largely emerge from the simulation. As a result, model parameterization required extensive knowledge and data, while the study reproduced the interactions among only three (nevertheless dominant) species among those comprising the investigated community.

Our objective is to develop an IBM of complete communities of marine benthic macroinvertebrates, explicitly representing the mechanisms that drive their spatial and temporal dynamics. To this end, we adapted the framework of Boulangeat et al. (2012), Boulangeat, Georges & Thuiller (2014) to marine benthos: we used available biological traits as proxies for the role of benthic species in general community assembly mechanisms, aiming to build groups with distinct functional roles through a systematic and testable procedure (Alexandridis et al., 2017a); we then employed well-established ecological theory and expert knowledge to derive rules of interaction among the functional groups and their resources (Alexandridis et al., 2017b). The use of proxy traits and first principles led to a preliminary semi-quantitative model parameterization, which was largely based on educated guesses.

In order to assess the potential of the approach to reproduce natural community patterns, we applied it to benthic macrofauna of the Rance estuary (Brittany, France). The estuary was isolated from the sea from 1963 to 1966 during the construction of a tidal power station and turned into a freshwater system. After the estuary was re-opened, it took its benthos about 10 years to reach compositional stability (Kirby & Retière, 2009). It currently shows high levels of local and regional diversity (Desroy, 1998), suggesting the existence of processes that operate at different spatial scales (Kneitel & Chase, 2004). Among community assembly mechanisms in estuarine systems, environmental filtering and larval dispersal operate at relatively coarse spatial scales (Ysebaert & Herman, 2002). Biotic interactions, characterized by functional trade-offs, appear to occur at much finer scales (Van Colen et al., 2008).

To our knowledge, there has been no spatially structured IBM representation of estuarine benthic communities. IBMs have mostly been used to study the spatially explicit dynamics of single species of plants (Kendrick, Marbà & Duarte, 2005) or animals (Van de Koppel et al., 2008) found on soft bottoms, which are typical of estuaries. The representation of spatially structured processes requires the development of models of different spatial scales. This need has been addressed by benthic IBMs through the mean-field representation of one spatial scale (Melbourne-Thomas et al., 2011) or through dynamically linked IBM simulations (Mumby & Dytham, 2006). A general lack of parameterized analytical formulations and our focus on mechanistic understanding rather than prediction favored the latter approach. Similarly to Mumby & Dytham (2006), we addressed the spatial mismatch between two scales through a toroidal, boundaryless depiction of space along with a mean representation of some processes. We intend to use this study’s results for the rigorous parameterization and validation of the model, with a view to broadening its exploratory and predictive scope.

## Materials and Methods

**Figure 1:**
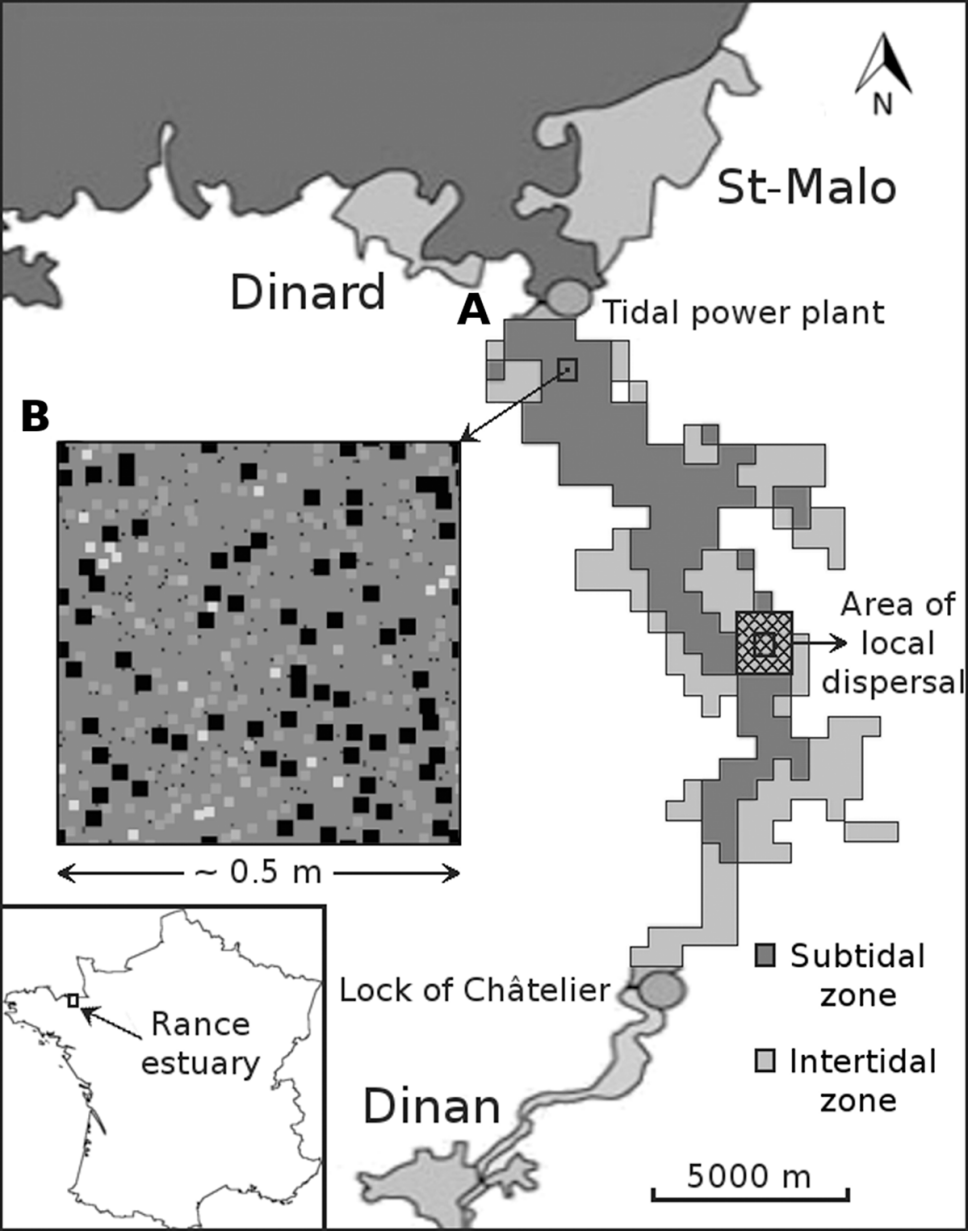
Graphical depiction of the coarse-scale model of the Rance estuary in France. Each cell of the subtidal and the intertidal zone (A) is represented by one instantiation of the respective fine-scale model (B). The latter has approximately the dimensions of a sampling area and its cells represent space occupied by individuals of different functional groups (illustrated as squares of different sizes and colors). Different instantiations of the fine-scale model interact through larval dispersal among the respective cells of the coarse-scale model. This process is limited to each cell’s immediate neighborhood (cross hatched square).

The model consists of two spatially structured cellular automaton sub-models (Fig. 1). In both sub-models, space is two-dimensional and a time step corresponds to one year. The fine-scale model represents a benthic sampling area of approximately 0.2 m^2^. The coarse-scale model represents the bottom of the entire Rance estuary. Each cell of the coarse-scale model has a one-to-one correspondence with one instantiation of the fine-scale model; still, the two have a spatial mismatch of three orders of magnitude.

**Figure 2:**
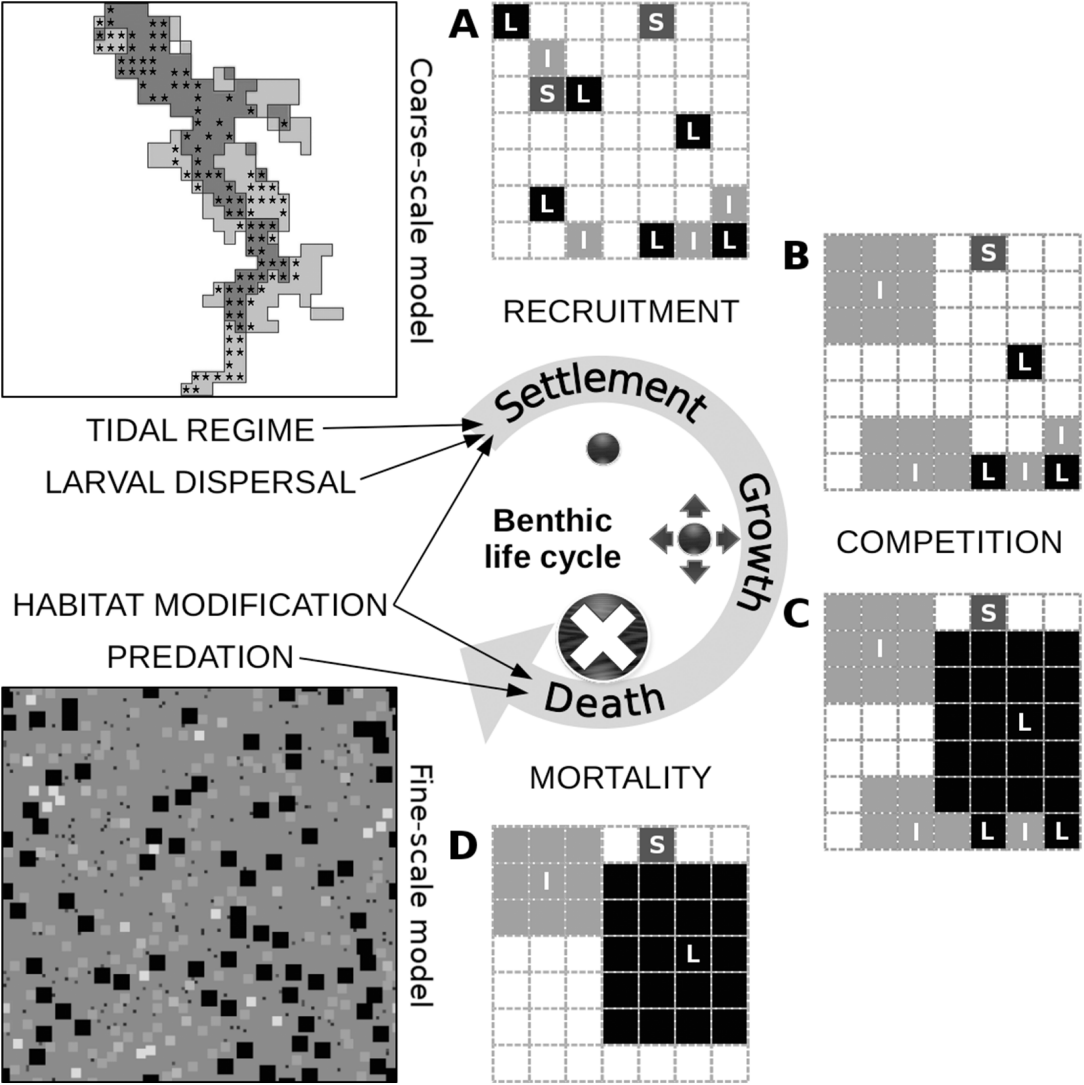
Conceptual diagram of the fine-scale model, built around a simplistic representation of the life cycle of benthic organisms. The settlement of a juvenile on the seabed is controlled by the tidal characteristics of the site and the relative abundance of its functional group in the area of local dispersal, both of which are defined at the level of the coarse-scale model, along with the relative abundance of sediment stabilizers and destabilizers in the juvenile’s close proximity, which is defined at the level of the fine-scale model. Juveniles of different groups that settle successfully and avoid post-settlement mortality generate patterns of recruitment (A). In this illustration of a segment of the fine-scale model, juveniles can belong to a group with small (S), intermediate (I) or large (L) adult body size and can occupy one cell on a grid that represents the seabed. Growth in terms of occupied space does not pertain to members of the small group. Members of the intermediate group grow first over all other juveniles, unless they are in close proximity to one another (B). The same restriction applies to members of the large group, which grow next over all other adults and juveniles (C). These growth processes result in both inter- and intra-group competitive interactions. Individuals that could consequently not grow or were grown over are assumed to be susceptible to various sources of mortality and are removed from the system (D). Death can also be caused by the loss of biogenic substrate and predation, both of which are represented at the level of the fine-scale model. The asterisks in the coarse-scale model indicate the position of the 113 cells that were selected for comparison with the empirical data.

The fine-scale model portrays the occupation of space by individual macroinvertebrates that belong to one of 20 functional groups. These groups were formed out of the 240 benthic macroinvertebrate species that were observed in the Rance estuary in 1995 (Desroy, 1998; for a list of species and their taxonomy, see section S1 in the Supplemental Material). The grouping was based on 14 biological traits describing initially the species’, and ultimately the groups’, role in a set of general community assembly mechanisms (Alexandridis et al., 2017a). The acceptable level of ecological aggregation was defined through evaluation of different grouping levels against the expectations of the emergent group hypothesis, i.e., functional differences among the groups should preserve the species’ niche attributes, while functional differences within them should be neutral (Hérault, 2007). The rules of the fine-scale model are implemented on the basis of the trait values of the group to which each individual belongs (Table 1). Individuals start as juveniles that settle in a cell on the seabed (Fig. 2). They remain on this cell, growing in terms of occupied space, unless they die due to competition, predation, loss of the organism on which they have settled (i.e., their basibiont) or ageing. The rules that describe the interaction of individuals with their environment are expected to represent the role of macroinvertebrates in the most important benthic community assembly mechanisms (Alexandridis et al., 2017b).

**Table 1:** Functional groups of species with their assigned representative species and biological trait values.

Groups	Representative species	T1 Temperature	T2 Development	T3 Dispersal	T4 Fecundity	T5 Tide/salinity	T6 Substrate	T7 Size (cm)	T8 Area	T9 Position	T10 Mobility	T11 Growth rate	T12 Lifespan (year)	T13 Epibiosis	T14 Engineering
FG1	*Myrianida edwardsi*	stenothermal	planktonic	long	low	stenohaline	mud	1.4	3.1	interface	mobile	5.8	1.9	neutral	neutral
FG2	*Thyasira flexuosa*	eurythermal	planktonic	short	low	stenohaline	mud	3.6	0.8	infauna	mobile	1.0	10.0	neutral	stabilizer
FG3	*Oligochaeta*	stenothermal	laid	short	low	emersed	muddy sand	4.5	5.0	infauna	mobile	3.4	2.0	neutral	destabilizer
FG4	*Notomastus latericeus*	stenothermal	brooded	short	low	stenohaline	muddy sand	6.0	2.9	interface	mobile	2.6	1.9	neutral	destabilizer
FG5	*Melinna palmata*	stenothermal	brooded	short	low	stenohaline	mud	7.5	0.3	interface	sessile	2.6	3.6	neutral	stabilizer
FG6	*Glycymeris glycymeris*	stenothermal	planktonic	short	high	stenohaline	muddy gravel	8.0	1.4	infauna	mobile	0.8	15.0	neutral	stabilizer
FG7	*Malacoceros fuliginosus*	eurythermal	planktonic	long	high	euryhaline	mud	8.5	1.9	interface	mobile	2.5	2.7	neutral	destabilizer
FG8	*Cerastoderma edule*	stenothermal	planktonic	long	high	emersed	muddy sand	8.6	0.5	interface	mobile	0.7	8.9	neutral	stabilizer
FG9	*Crepidula fornicata*	stenothermal	planktonic	long	high	stenohaline	rock	7.6	0.0	epifauna	sessile	1.9	11.2	basibiont	neutral
FG10	*Galathowenia oculata*	eurythermal	planktonic	long	high	euryhaline	mud	11.1	0.0	interface	sessile	2.7	4.4	neutral	stabilizer
FG11	*Hediste diversicolor*	eurythermal	laid	short	high	emersed	muddy sand	12.8	0.2	interface	mobile	2.1	3.4	neutral	destabilizer
FG12	*Sphaerosyllis bulbosa*	stenothermal	brooded	short	low	stenohaline	gravel	1.3	0.5	epifauna	mobile	4.7	1.9	neutral	neutral
FG13	*Balanus crenatus*	eurythermal	planktonic	long	high	euryhaline	rock	2.0	0.8	epifauna	sessile	2.5	2.0	epibiont	neutral
FG14	*Morchellium argus*	eurythermal	brooded	short	low	stenohaline	rock	3.3	0.1	epifauna	sessile	2.6	1.7	epibiont	neutral
FG15	*Anapagurus hyndmanni*	stenothermal	planktonic	long	high	stenohaline	gravel	10.0	0.1	epifauna	mobile	0.6	10.0	neutral	neutral
FG16	*Lepidochitona cinerea*	stenothermal	planktonic	short	high	stenohaline	rock	10.8	4.1	epifauna	mobile	0.9	11.6	epibiont	neutral
FGP1	*Syllis cornuta*	stenothermal	planktonic	long	low	stenohaline	rock	7.4	5.2	epifauna	mobile	2.3	2.3	epibiont	neutral
FGP2	*Marphysa bellii*	stenothermal	planktonic	short	high	stenohaline	muddy sand	23.3	0.3	interface	mobile	1.1	4.7	neutral	neutral
FGP3	*Nephtys hombergii*	stenothermal	planktonic	long	high	stenohaline	gravel	10.5	0.3	interface	mobile	2.2	7.3	neutral	neutral
FGP4	*Urticina felina*	eurythermal	planktonic	short	high	euryhaline	rock	16.7	10.3	epifauna	sessile	1.1	14.0	epibiont	neutral

**Note:**

Group names starting with ‘FG’ and ‘FGP’ correspond to algae/detritus feeders and predators/scavengers, respectively for details, see Alexandridis et al. (2017a).

The coarse-scale model portrays the distinction between the subtidal (i.e., below the low tide level) and the intertidal (i.e., between the low and high tide level) zones of the seabed and the interaction of populations of groups through larval dispersal. A cell of the coarse-scale model corresponds to an instantiation of one of two versions of the fine-scale model, depending on whether the cell belongs to the subtidal or the intertidal zone. Neighboring cells of the coarse-scale model interact at each time step through the dispersal of larvae from each cell’s populations of groups.

General difficulties in the evaluation of IBMs have motivated the development of the ODD (Overview, Design concepts and Details) protocol (Grimm et al., 2006). The authors proposed a standard structure for model documentation, aimed at facilitating reading and writing the description of IBMs (Grimm et al., 2010). The description of the models below is based on elements of the ODD protocol (for a detailed account of the fine-scale model, see section S2 in the Supplemental Material).

## Fine-scale model

### Basic principles

Two versions of the model represent differences in the settlement success of benthic organisms between the subtidal and the intertidal zone. The model represents inter- and intra-group competitive interactions within macroinvertebrate communities, by recreating the life cycle of individuals, from their recruitment as juveniles, to their growth and death. These basic features are further modified through a basic representation of predation and biogenic habitat modification in the form of epibiosis (i.e., living on the surface of a living organism) and sediment engineering (i.e., modifying, maintaining and creating habitats by modulating sediment characteristics).

The supply of larvae and the settlement of juveniles are key factors shaping benthic communities, but their complexity often renders simplifications particularly difficult (Pineda, Reyns & Starczak, 2009). Recruitment processes should, at least partly, depend on the size of the spawner pool (i.e., the sum of adult individuals within a specific area) and the salinity preferences of each organism. They are also expected to be influenced by biological traits, such as fecundity, dispersal distance and early development mode, whose values often form combinations indicative of important life history trade-offs (Kupriyanova et al., 2001). The recruitment of juvenile organisms is also significantly impacted by post-settlement mortality, which shows high levels of both intra- and inter-specific variation (Hunt & Scheibling, 1997). Information at the former level indicates that small initial increments in juvenile body size lead to significant increases in survival rates in the face of predation (Gosselin & Qian, 1997).

The successional dynamics of benthic systems have been found to feature space limitation due to adult–juvenile interactions and exploitative competition for food, with the functional role of organisms largely defined by their size (Van Colen et al., 2008). Smaller organisms are expected to show higher growth rates and be better competitors for limited amounts of space, while larger organisms can occupy larger areas and should be competitively superior in the face of food limitation (Alexandridis et al., 2017b). Competitively equivalent individuals also compete for food and space, which should affect their growth rates (Côté, Himmelman & Claereboudt, 1994). Trophic distinctions, like the one between suspension- and deposit-feeders, can be invalid in view of the highly facultative feeding behavior of benthic organisms (Snelgrove & Butman, 1994). Trophic interactions could, instead, be dictated by expert knowledge and theoretically anticipated allometries, which should increase the realism and potential for stability of model representations (Brose, Williams & Martinez, 2006b).

The majority of predators appear to be larger than their prey and predator size tends to increase with prey size (Cohen et al., 1993). On the other hand, predator–prey body-size ratios are generally the lowest, just over two on average, for marine invertebrates, compared to other taxonomic groups and habitat types. It is possible that the energetic costs of prey capture and consumption set a limit to predator–prey size differences (Brose et al., 2006a). In the Rance estuary, fish predation of benthic organisms is restricted and predation by birds is highly seasonal and mostly limited to the intertidal zone. The mortality caused by predatory macroinvertebrates appears likewise to be limited in magnitude, as far as adult prey is concerned. This is due partly to the greater impact of predation on juveniles and partly to the partial ingestion of adults and the regenerative properties of many of them. Predation pressure should depend on the organisms’ defensive mechanisms and position in the sediment, along with their relative abundances (Desroy, 1998).

Benthic macroinvertebrates in the Rance estuary appear to differ with regard to their use of space, between those that are buried in the sediment and those that occupy its surface supported by hard substrates (Alexandridis et al., 2017b). The dominance of soft bottoms in the estuary indicates that the latter organisms partly occur due to the benefits provided by the phenomenon of epibiosis (Wahl, 1989). The settlement of basibiotic (i.e., providing substrate for other organisms) individuals should be facilitated by the habitat modification effect of other basibionts, while the settlement success of epibionts (i.e., organisms living on the surface of other organisms) should depend on their specific substrate preferences. Still, sediment engineering is expected to play a much more prominent role in soft bottom systems (Meadows, Meadows & Murray, 2012). Its impact on benthic communities can be summarized by the mobility-mode hypothesis, which groups organisms into sediment stabilizers and destabilizers (Posey, 1987). The dominance of the largest members of a group in an area should allow them to modify sediment characteristics in a way that facilitates the settlement of the group’s own members, while inhibiting members of the other group.

### Entities, state variables and scales

The only entities are cells that make up a square grid of 60 × 60 cells. In order to avoid edge effects, the grid wraps horizontally and vertically into a torus. Cell size corresponds to the theoretical area exclusively occupied by an individual that belongs to one of the small groups. The dimensions of the grid represent an arbitrary sampling area of the real system. One time step corresponds to one year, starting right before spring larval dispersal.

A cell variable indicates whether each cell of the model is occupied and by a member of which functional group. There are twenty groups in the model (Table 2). Ten of them belong to the infauna (i.e., animals living inside the sediment) (FG1–8, FG10–11), five belong to the epifauna (i.e., animals living on the seabed’s surface) (FG12–16) and one can be part of either the infauna or the epifauna (FG9); all of these groups represent algae/detritus feeders. Four more groups represent predator/scavenger organisms (FGP1–4).

**Table 2:** Parameterization of the subtidal and the intertidal version of the fine-scale model.

Groups	Size	Position in substrate	Post-settlement mortality rate (%)	Settlement probability multiplication factors						Habitat modification		Potential prey groups
				Subtidal			Intertidal			Subtidal	Intertidal	
				Initial	Stabilized	Destabilized	Initial	Stabilized	Destabilized			
FG1	S	infauna	90	2/3	1	1	1/3	1	1	–	–	–
FG2	S	infauna	90	1/3	1/2	1/2	1/6	1	1	–	–	–
FG3	S	infauna	90	2/3	1/2	1/2	2/3	1/2	2	–	–	–
FG4	S	infauna	90	1	1/2	2	1/2	1	1	–	–	–
FG5	M	infauna	50	1	2	1/2	1/2	1	1	stabilizer	–	–
FG6	M	infauna	50	2/3	1/2	1/2	1/3	1	1	–	–	–
FG7	M	infauna	50	1	1/2	2	1	1/2	2	destabilizer	destabilizer	–
FG8	M	infauna	50	1	1/2	1/2	1	2	1/2	–	stabilizer	–
FG9	M	in-/epifauna	50	1	2	1/2	1/2	1	1	basibiont	basibiont	–
FG10	L	infauna	10	1	2	1/2	1	2	1/2	stabilizer	stabilizer	–
FG11	L	infauna	10	1	1/2	2	1	1/2	2	destabilizer	destabilizer	–
FG12	S	epifauna	90	1	–	–	1/2	–	–	–	–	–
FG13	S	epifauna	90	1	–	–	1	–	–	–	–	–
FG14	S	epifauna	90	1	–	–	1/2	–	–	–	–	–
FG15	L	epifauna	10	1	–	–	1/2	–	–	–	–	–
FG16	L	epifauna	10	2/3	–	–	1/3	–	–	–	–	–
FGP1	–	–	–	–	–	–	–	–	–	–	–	FG4, FG7
FGP2	–	–	–	–	–	–	–	–	–	–	–	FG7, FG11
FGP3	–	–	–	–	–	–	–	–	–	–	–	FG4, FG5
FGP4	–	–	–	–	–	–	–	–	–	–	–	FGP1, FGP3

**Note:**

Parameter values used at the model’s initialization (Initial) and run, in the case that functional group abundances are dominated by sediment stabilizing (Stabilized) or destabilizing groups (Destabilized).

The infauna consists of four small groups (FG1–4), which can occupy one cell, five intermediate groups (FG5–9), which start with one cell and can occupy its eight immediate neighbors during their growth, and two large groups (FG10–11), which can, through the same procedure, occupy one cell and its twenty-four closest neighbors. The group that belongs to the infauna or the epifauna (FG9) represents basibiotic organisms of intermediate size. Epifauna, including individuals of the latter group but staying small in size, can settle in cells occupied by this group. In total, epifauna consists of four small groups (FG9, FG12–14), which can occupy one cell, and two large groups (FG15–16), which start with one cell and can occupy its eight immediate neighbors during their growth.

### Process overview and scheduling

The first of the model’s actions represents the process of recruitment. Juveniles of the eleven infaunal groups settle randomly in empty cells, with probabilities defined by the contribution of each group to the infaunal spawner pool, along with the relative abundance of sediment stabilizing and destabilizing groups. Infaunal juveniles experience random post-settlement mortality and those that die are removed from the system. Juveniles of the six epifaunal groups settle randomly in cells that are occupied by infaunal adults of the basibiotic group and are empty of epibionts, with probabilities defined by the contribution of each group to the epifaunal spawner pool. Epifaunal juveniles experience random post-settlement mortality and those that die are removed from the system. During the first time step, all cells are considered empty, which corresponds with the initial conditions after the estuary was re-opened in 1966, and all groups of the infauna and the epifauna have equal contributions to the respective spawner pool.

The second action represents the growth in terms of occupied cells of juveniles that belong to groups with intermediate and large size and the process of inter- and intra-group competition for food and space that this entails. First, the juveniles of intermediate infaunal groups, then only those infaunal juveniles of the basibiotic group that settled near other group members and finally the juveniles of large infaunal groups grow in random order within each of these categories. The juveniles of the two large epifaunal groups are next to grow, also in random order within each category, first those of the group that is associated with hard substrate and then those of the group that is associated with gravel. During these growth processes, juveniles that grow earlier are allowed to grow over non-growing individuals and juveniles that grow later. Those of the latter that have not been grown over are allowed to grow over all other individuals. Juveniles are in all cases not allowed to grow over individuals within the same category.

The third action of the model represents the process of ageing by one year of all individuals that survived the previous time step.

During the fourth and final action, all individuals that could not grow to their full predefined size, were grown over, reached their lifespan during the current time step or were epibionts of deceased basibionts that could not be replaced, die and are removed from the system. Basibiotic individuals that die of ageing and have epibionts of the same group, take the age of their oldest epibiotic basibiont and retain the rest of their epibionts. Individuals of potential prey groups are then randomly selected to be removed due to predation. This is done in decreasing order of the predators’ size and starts with their most abundant prey group. If this is less abundant than each predator’s total prey, individuals from its next most abundant prey group are additionally removed, until the number of individuals removed is the closest possible to each predator’s total prey.

## Fine-scale model parameterization

The representation of the modeled processes is of highly qualitative nature, in the sense that parameterization is based on extreme or intermediate values along a continuum of possible options. The values were selected to reflect the previously stated basic principles of the fine-scale model and can in most cases be considered as educated guesses. The behavior of the different functional groups was parameterized on the basis of their values for the 14 biological traits, which are below indicated as (T#), in reference to the trait coding of Table 1. An overview of the model parameterization is given in Table 2.

### Subtidal settlement probabilities

The settlement probabilities of all infaunal and epifaunal groups that are employed during the model’s initialization are defined by each group’s early development mode (T2), dispersal distance (T3) and fecundity (T4). Specifically, groups with brooded early development mode (i.e., development of eggs or juveniles under parental care) (FG4, FG5, FG12, FG14) or long dispersal distance and high fecundity (FG7, FG8, FG9, FG10, FG13, FG15), along with groups with any of the last two trait levels and laid early development mode (i.e., development of eggs actively deposited on the substrate) (FG11), have settlement probabilities that are three times the settlement probabilities of groups with none of the aforementioned trait levels (FG2). The latter groups have settlement probabilities that are half the settlement probabilities of groups with planktonic early development mode (i.e., development in the water column without the ability to swim against a current) and either long dispersal distance (FG1) or high fecundity (FG6, FG16), along with groups with short dispersal distance, low fecundity and laid early development mode (FG3).

The settlement probabilities of infaunal and epifaunal groups that are employed during the model’s action of recruitment use the settlement probabilities employed during the model’s initialization, multiplied by a factor proportional to each group’s contribution to the respective spawner pool. The settlement probabilities of infaunal groups are additionally determined by each group’s position in the sediment (T9) and role in sediment engineering (T14), along with the relative abundance of sediment stabilizing and destabilizing groups. This represents the impact of sediment engineering and is applied as follows. First, the settlement probabilities of mobile stabilizers and groups that live deep in the sediment (FG2, FG3, FG6, FG8) are divided by 2. The rest of the rules apply only to the remaining infaunal groups. If the effective sediment stabilizers (intermediate and large sessile stabilizers FG5 and FG10) outnumber the effective destabilizers (intermediate and large destabilizers FG7 and FG11), the settlement probabilities of the stabilizers (FG5, FG10) and the basibiotic group FG9 are multiplied by 2 and those of the destabilizers (FG4, FG7, FG11) are divided by 2. Otherwise, the settlement probabilities of the stabilizers (FG5, FG10) and the basibiotic group FG9 are divided by 2 and those of the destabilizers (FG4, FG7, FG11) are multiplied by 2. The settlement probability of the infaunal group that is neutral with respect to epibiosis and sediment engineering (FG1) is not modified during this process.

### Intertidal settlement probabilities

The settlement probabilities of all infaunal and epifaunal groups that are employed during the model’s initialization are partly defined by each group’s early development mode (T2), dispersal distance (T3), and fecundity (T4). The same multiplication factors are therefore used as those employed during the initialization of the subtidal version of the model. These factors are additionally modified based on each group’s tolerance for low salinity and tidal exposure (T5). Specifically, the settlement probabilities of all stenohaline (i.e., not tolerating long tidal exposure and salinities that differ greatly from those of the open sea) groups (FG1, FG2, FG4, FG5, FG6, FG9, FG12, FG14, FG15, FG16) are divided by 2.

The settlement probabilities of infaunal and epifaunal groups that are employed during the model’s action of recruitment use the settlement probabilities employed during the model’s initialization, multiplied by a factor proportional to each group’s contribution to the respective spawner pool. The settlement probabilities of infaunal groups are additionally determined by each group’s role in sediment engineering (T14) and the relative abundance of sediment stabilizing and destabilizing groups. This represents the impact of sediment engineering and is applied similarly to the subtidal version of the model; only this time the settlement probabilities of euryhaline (i.e., tolerating salinities that differ greatly from those of the open sea) and emersed (i.e., tolerating long tidal exposure) groups (FG3, FG7, FG8, FG10, FG11) are modified. If the effective sediment stabilizers (intermediate and large, euryhaline and emersed stabilizers FG8 and FG10) outnumber the effective destabilizers (intermediate and large, euryhaline and emersed destabilizers FG7 and FG11), the settlement probabilities of the stabilizers (FG8, FG10) are multiplied by 2 and those of the destabilizers (FG3, FG7, FG11) are divided by 2. Otherwise, the settlement probabilities of the stabilizers (FG8, FG10) are divided by 2 and those of the destabilizers (FG3, FG7, FG11) are multiplied by 2.

### Post-settlement mortality

The mortality rates experienced by juveniles of all groups after their settlement on the seabed are defined by each group’s body size (T7). The selected values represent extreme and intermediate levels observed in nature. A fraction of the juveniles’ abundance equivalent to 90% for the small groups, 50% for the intermediate groups and 10% for the large groups are removed from the system following their settlement.

### Predation

The potential prey of the predator groups consists of groups that are smaller or similar in size but no smaller than 1/3 of their own size (T7), are not buried deep in the sediment (T9) and their representative species are not protected by plates, shells or tubes. Specifically, FGP1 has groups FG4 and FG7 as its potential prey, while the potential prey of FGP2 consists of FG7 and FG11. The sessile predator group FGP4 is additionally limited to mobile epifaunal organisms (T10) and can, therefore, feed on groups FGP1 and FGP3. The availability of precise information on the diet of the representative species of predator group FGP3 in the Rance estuary allows the assignment of groups FG4 and FG5 as its potential prey. The abundance of predator groups at each time step is equal to 1/10 of the total abundance of their potential prey in the subtidal and 1/10 of the abundance of the most abundant group among their potential prey in the intertidal version of the model, both observed at the previous time step. The 1/10 value was derived by assuming 10% energy transfer efficiency between the two trophic levels, as well as similar values of biomass-relative productivity and individual biomass. The less and more conservative levels of total and most abundant potential prey were used in the subtidal and the intertidal version of the model, respectively. The total number of individuals of the potential prey groups that explicitly die due to predation and are removed from the system at each time step is equal to the abundance of their respective predators.

## Coarse-scale model

### Basic principles

The spatial scales at which pre- and post-settlement processes take place are not expected to overlap considerably, as pre-settlement processes usually operate at much coarser scales than post-settlement ones (Fraschetti et al., 2002). Exchanges across spatial scales are mainly the result of larval dispersal; immigration and emigration of adults can in most cases be considered as trivial for the population dynamics of benthic organisms (Eckman, 1996).

### Initialization

The grid of the coarse-scale model represents the bottom of the Rance estuary, with cell size corresponding to an area of approximately 0.2 km^2^. A sediment type is attributed to each cell, based on a sedimentary map of the Rance estuary from 1994 (Bonnot-Courtois, 1997). Each cell is then assigned to the subtidal or the intertidal zone, based on its sediment type. Areas covered by gravel, coarse sand, intermediate/coarse sand, fine/intermediate sand, muddy sand and sandy mud are assigned to the subtidal zone. Areas covered by silty mud, mud, pure mud and salt marshes are assigned to the intertidal zone.

The subtidal version of the fine-scale model is the first to be loaded and the cells of the subtidal zone, in random order, ask it to initialize and export the generated model instances in files named after their own *x* and *y* coordinates. At the same time, each cell of the subtidal zone is attributed with the generated group abundances, which are printed out, as well as a color on the grid, which indicates whether these abundances are dominated by sediment stabilizers or destabilizers. The same procedure is then repeated for the cells of the intertidal zone and the intertidal version of the fine-scale model.

### Process overview and scheduling

One time step corresponds to one year, starting right before spring dispersal. The cells of the subtidal zone, in random order, ask the subtidal version of the fine-scale model to import the model instances that were generated for them during the previous time step and set each infaunal and epifaunal group’s contribution to the respective spawner pool equal to the median abundance of each group within the cells themselves and their eight immediate neighbors that are part of the system. Within the same procedure, the cells ask the fine-scale model to move one step forward and export the generated model instances in files named after their *x* and *y* coordinates. At the same time, each cell is attributed with the generated group abundances, which are printed out, as well as a color on the grid, which indicates whether these abundances are dominated by sediment stabilizers or destabilizers. The same procedure is then repeated for the cells of the intertidal zone and the intertidal version of the fine-scale model.

## Model analysis

The lack of detailed knowledge on a number of important ecological processes allowed only their very basic, often semi-quantitative, representation. Accordingly, the analysis of the model focused on its structural characteristics and theoretical background, rather than its parameterization or validation. The effect of spatial resolution on the model output is not presented, as doubling its value for either the fine- or the coarse-scale model led to qualitatively similar results.

### Model simulation

A 10-year simulation of the model was replicated three times. The number of time steps was limited to 10, because the possibility of all sites in the Rance estuary to stay undisturbed and evolve concurrently should decrease significantly as the number of years increases. The choice of three replicate simulations was made for practical reasons. The simulation that produced the highest level of β-diversity (see section Sensitivity analysis) was singled out for a more detailed analysis of the model output (hereafter called the “benchmark simulation”).

### Sensitivity analysis

Four modifications were independently applied to different elements of the model, in order to examine the role of the respective processes. The role of dispersal distance was examined first. The contribution of each infaunal and epifaunal group to the respective spawner pool in each cell of the coarse-scale model was derived from group abundances in all of the model’s cells, instead of just each cell’s immediate neighbors. The role of post-settlement mortality was examined next, by completely removing it from the fine-scale model. The role of sediment engineering was examined by eliminating its effect on the settlement probabilities used in the fine-scale model. Finally, the role of predation was examined by removing explicit predation mortality from the fine-scale model. Each of these model configurations was simulated for 10 years and replicated three times.

Changes in the model’s behavior were investigated by depicting the evolution of modelled β-diversity, compared to the value that was observed at the level of functional groups in the Rance estuary in 1995 (data can be found in section S3 in the Supplemental Material). β-diversity was in all cases quantified as the variance of a Hellinger-transformed table of group abundances in different sites (Legendre & De Cáceres, 2013). In the case of the observations, the sites correspond to 113 stations (71 subtidal, 42 intertidal) that were sampled in the Rance estuary in 1995. In the case of the output of the five different model configurations, the sites correspond to 113 of the 230 cells of the coarse-scale model (71 subtidal, 42 intertidal; see Fig. 2 for the exact position of the cells in the model). These cells were used with the goal of approximating the location in the estuary of the sampled stations.

### Correspondence analysis

Correspondence analysis (Legendre & Legendre, 1998) was first performed on the table of functional group abundances in 113 stations that were sampled in the Rance estuary in 1995. It was then performed on tables of group abundances in 113 cells selected out of the 230 cells of the coarse-scale model (see section Sensitivity analysis) in the 1st, 2nd, 3rd and 10th year of the benchmark simulation. These years were chosen to represent the qualitatively greatest changes in model output. All tables of group abundances were Hellinger-transformed before analysis. The goal was to compare patterns in the relative frequencies of functional groups across stations or cells, so scaling 2 was selected for the projection of groups on the first two axes of the reduced multivariate space (Borcard, Gillet & Legendre, 2011).

### Spatial correlation

Mantel correlograms were used to quantify spatial correlation in the multivariate domain of functional group abundances. The technique is based on calculation of the normalized Mantel statistic between pairs of site dissimilarity matrices. One matrix in each pair quantifies differences in multivariate community composition and the other is derived by attributing the value 0 to pairs of sites that belong to the same distance class and the value 1 to all other pairs of sites. The process is repeated for each distance class and values of the Mantel statistic are tested by permutations. Mantel correlograms were produced for the 113 stations that were sampled in the Rance estuary in 1995 and the output of the 1st, 2nd, 3rd and 10th year of the benchmark simulation in 113 cells selected out of the 230 cells of the coarse-scale model (see section Sensitivity analysis). Distances in the model were measured between the cells’ centers of symmetry, by assuming cell dimensions of 450 m × 450 m. The tables of group abundances were Hellinger-transformed and Holm’s correction for multiple testing was applied to the permutation tests. The number of distance classes was in each case based on Sturge’s rule and the correlograms were restricted to distances that included all sites (Borcard, Gillet & Legendre, 2011).

### Software

Simulations of both the fine- and the coarse-scale model were implemented in the multi-agent modeling environment NetLogo version 5.3.1 (Wilensky, 1999). Interactions between the two scales were realized through the NetLogo extension LevelSpace (Hjorth, Head & Wilensky, 2015). The source code of the subtidal and the intertidal version of the fine-scale model and the coarse-scale model can be found in section S4 in the Supplemental Material. The NetLogo model files and the GIS data of substrate types in the Rance estuary are also available in the Supplemental Material. All model analyses were performed using the statistical software R version 3.2.2 (R Core Team, 2015) with the packages vegan (Oksanen et al., 2015) and raster (Hijmans, 2016) and the function beta.div (Legendre & De Cáceres, 2013, Appendix S4).

## Results

### Model simulation

A common pattern in all simulations of the standard model configuration was the initial presence of all functional groups in most cells of the coarse-scale model and the gradual dominance of different groups in different sets of cells (see section S5 in the Supplemental Material). During this transition, three obligate epibiotic groups (FG12–14) initially became rare and were eventually eliminated from the system. The median number of groups per cell of the coarse-scale model declined from 20 at initialization to 11 after 10 years of simulation in all three replicates. The maximum and minimum number of groups per cell in the 10th year was also the same in all replicate simulations, equal to 16 and four, respectively. The general trend was for the whole system to be covered by stabilizer-dominated cells. Pockets of resistance to this trend were, however, formed by destabilizer-dominated cells of the subtidal and the intertidal zones, within which the opposite trend could be observed.

### Sensitivity analysis

**Figure 3:**
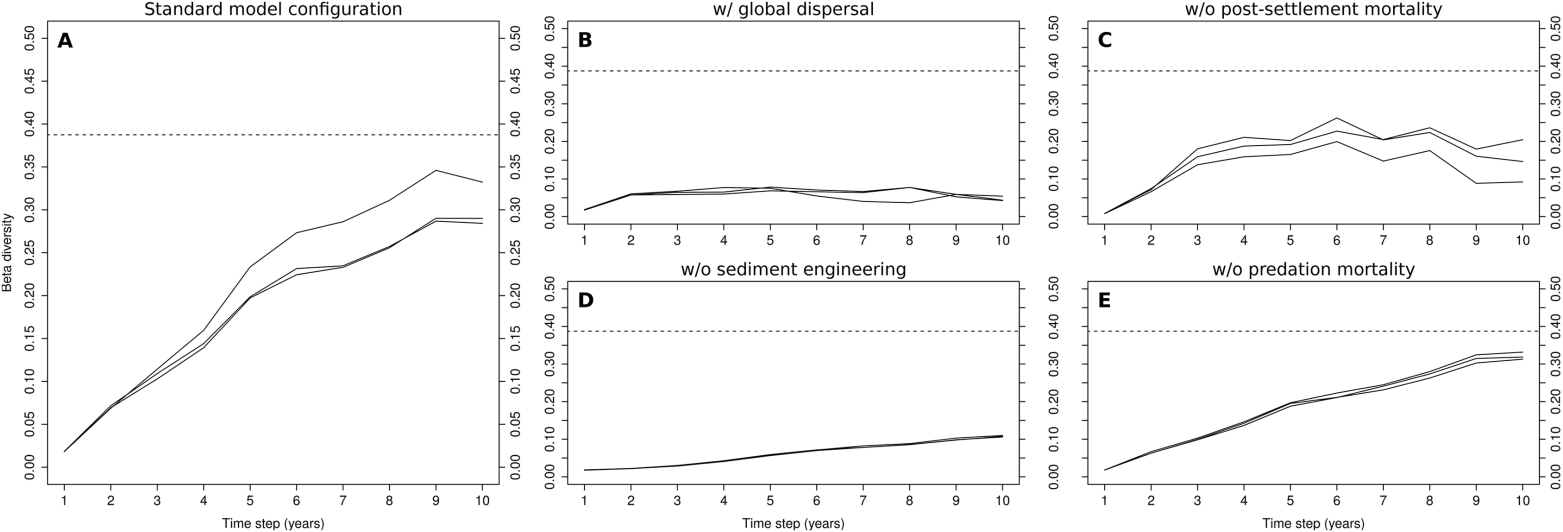
Evolution of β-diversity in three 10-year simulations of different model configurations. Output from the model (A) with standard configuration, (B) with global dispersal, (C) without post-settlement mortality, (D) without sediment engineering and (E) without predation mortality. The dotted lines indicate the level of β-diversity that was observed in the Rance estuary in 1995.

The evolution of β-diversity during the 10-year simulations of the standard model configuration shows a clear increasing trend during the first nine years, after which it levels off (Fig. 3A). The levels reached in the 9th year were, at least in one of the three simulations (0.35), close to those observed in the Rance estuary (0.38). The replacement of local dispersal by its global counterpart drastically changed β-diversity in the modeled system (Fig. 3B), as no clusters of cells that were dominated by sediment stabilizers or destabilizers were formed. The very low levels of β-diversity that were reached in this case were the result of the distinction between the two tidal zones, along with stochasticity regarding the relative abundances of habitat modification groups in the fine-scale model’s initialization. Without sediment engineering, this stochastic effect was removed and all simulations had almost identical output (Fig. 3D). β-diversity increased at a very slow rate, as differences in community composition were gradually amplified through local dispersal. The removal of post-settlement mortality allowed small groups to overwhelmingly dominate the fine-scale model, resulting in unrealistic abundance levels and low β-diversity (Fig. 3C). The removal of predation mortality had a minimal impact on β-diversity (Fig. 3E). Still, the values that were observed in the 10th year were slightly higher than those of the standard model configuration.

### Correspondence analysis

**Figure 4:**
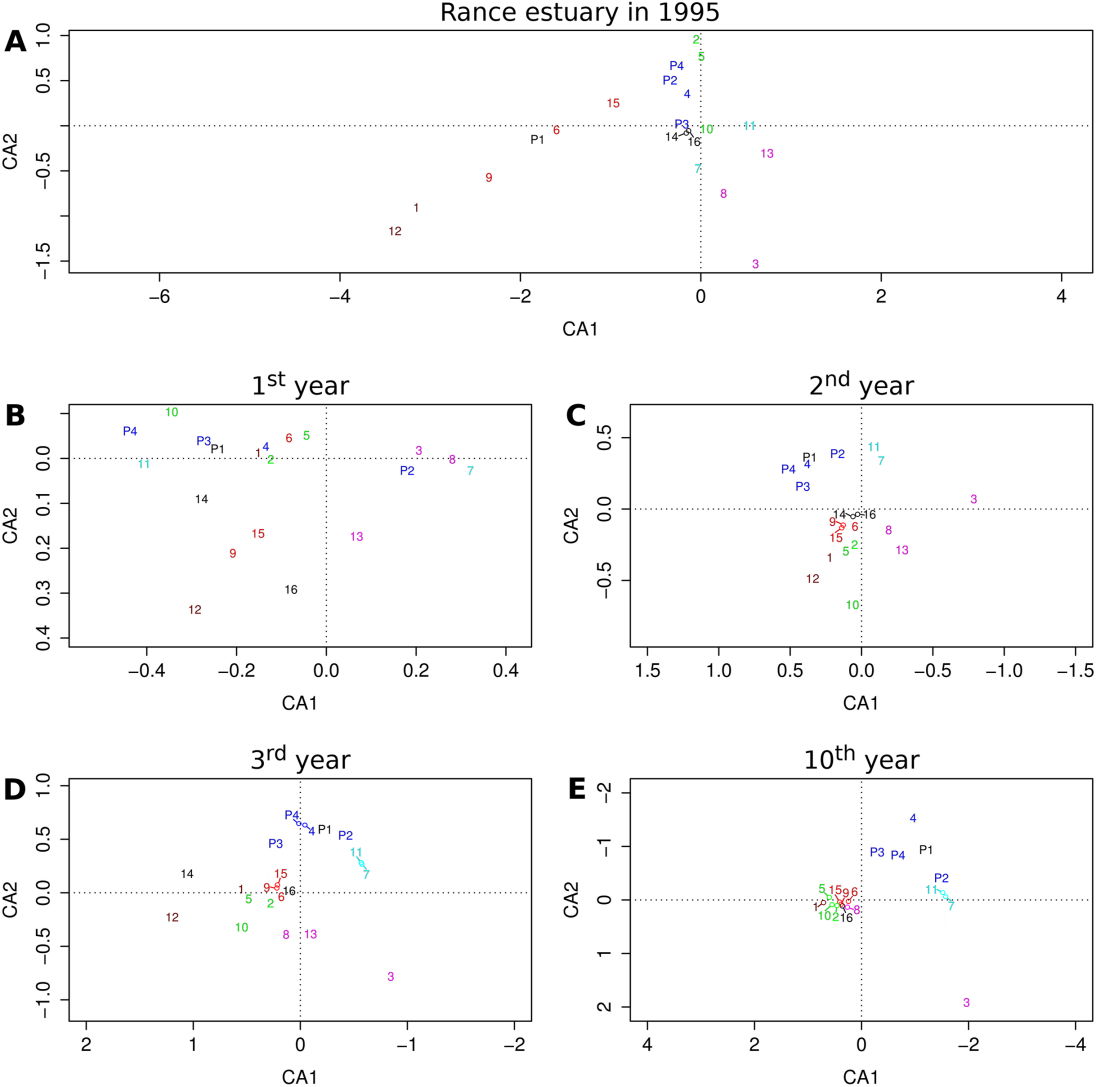
Projections of functional groups on the first two axes of correspondence analysis. Results for (A) the observations from the Rance estuary in 1995 and the model output in the (B) 1st, (C) 2nd, (D) 3rd and (E) 10th year of the benchmark simulation. Numbers 1–16 refer to groups FG1–FG16 and numbers P1–P4 refer to groups FGP1–FGP4. Colors indicate similarities between empirical data and model output, regarding the association of habitat modification neutral (brown), intertidal (purple), basibiotic and gravel-associated (red), predator and subtidal prey (blue), intertidal prey (cyan) and mud-associated stabilizer (green) groups.

The relative position of groups as they are projected on the first two axes of the reduced multivariate space illustrates their similarity with regard to their relative frequencies across sites or cells (Fig. 4). The first two axes of the correspondence analysis that was performed on observed group abundances (Fig. 4A) represent only about 32% of the total variation, whereas the same value for the output of the 1st, 2nd, 3rd and 10th year of the benchmark simulation (Fig. 4B–4E) equals 72%, 84%, 79% and 73%, respectively, all above 70%.

Some of the patterns that can be seen in the observed group associations are also evident in the model output. The observed association of groups FG1 and FG12, both of which are neutral with regard to epibiosis and sediment engineering, can be seen after the first simulation year. The same holds true for the association of the intertidal groups FG3, FG8 and FG13. Groups FG12 and FG13 gradually get eliminated and groups FG1 and FG8 converge toward the majority of the groups, but the separation of group FG3 remains a constant feature of the model output. The basibiotic group FG9 is positioned near the gravel-associated groups FG6 and FG15 in both the observations and the initial simulation years, before epibiosis is largely eliminated from the modeled system. The association of subtidal prey group FG4 and, to a lesser extent, intertidal prey groups FG7 and FG11 with predatory groups FGP2, FGP3 and FGP4 is also a pattern shared by the observations and the entire simulation period after the initialization, as is the association of mud-associated, stabilizer groups FG2, FG5 and FG10.

### Spatial correlation

**Figure 5:**
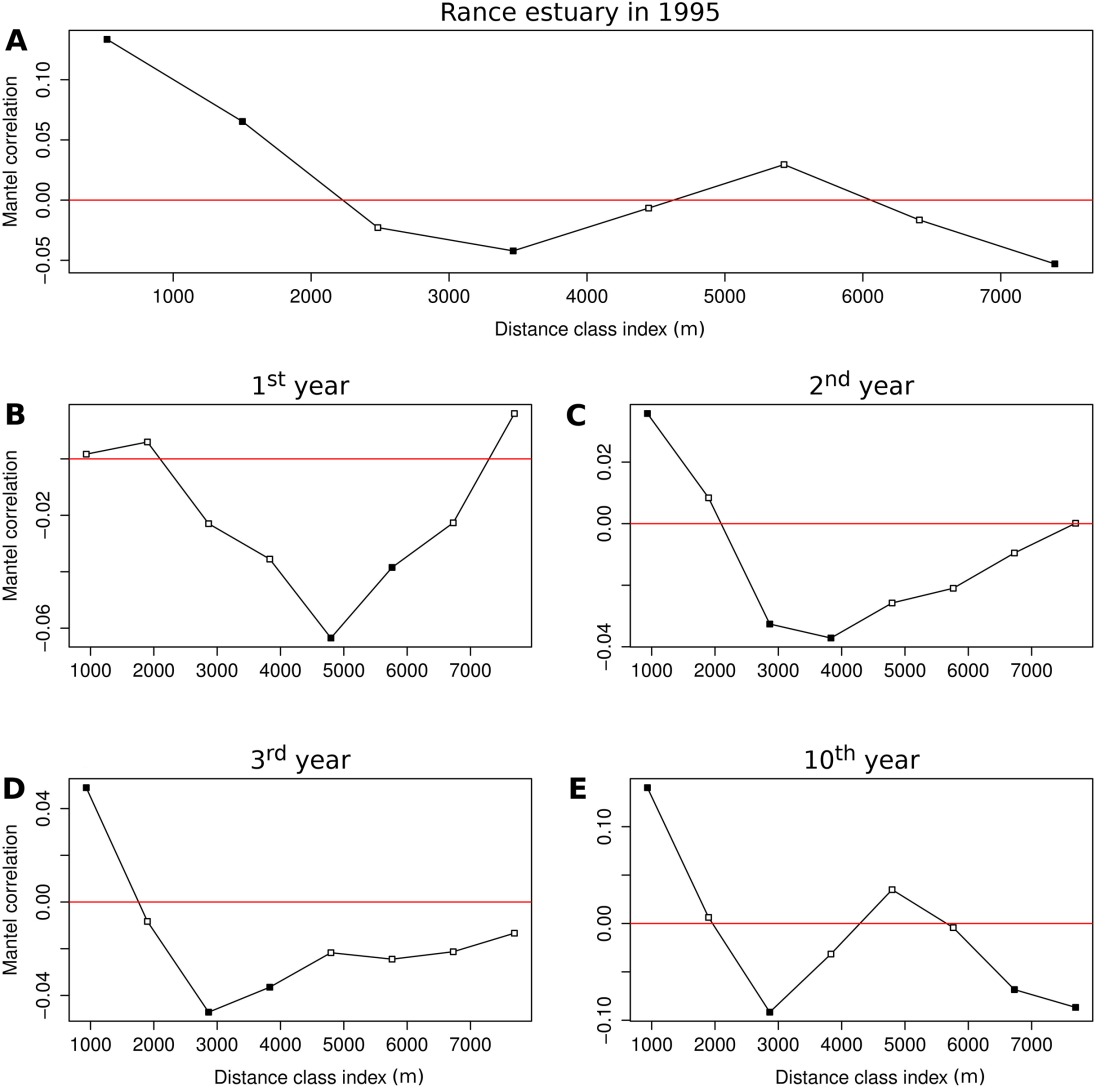
Correlograms of the Mantel statistic in different distance classes. Results for (A) the observations from the Rance estuary in 1995 and the model output in the (B) 1st, (C) 2nd, (D) 3rd and (E) 10th year of the benchmark simulation. Filled squares indicate statistically significant values at 0.05 level.

The Mantel correlogram of the observed group abundances demonstrates significantly similar community composition within distances of 0.5 and 1.5 km and significant compositional dissimilarity at distances of 3.5 and 7.5 km (Fig. 5A). Spatial correlation in the 1st year of the benchmark simulation was demonstrated as significantly dissimilar community composition at distances around 5 and 6 km (Fig. 5B). Similar levels of compositional dissimilarity were observed at slightly shorter distances in the subsequent simulation years (Figs. 5C and 5D), accompanied by significantly similar community composition within a distance of 1 km. The latter feature was retained in the 10th year (Fig. 5E), along with significant compositional dissimilarity at distances of 3 km and around 7 and 8 km, in a pattern that is similar to the one observed in the empirical data.

## Discussion

### Mechanistic approach

This study aimed to explore the potential to reproduce the dynamics of benthic macroinvertebrate communities through a mechanistic representation of the system, based primarily on theoretical expectations about community assembly mechanisms. The first step was taken with the reduction of the system’s components through a systematic and testable procedure that retained sufficient information on the organisms’ functional role (Alexandridis et al., 2017a). The verification of the basic assumptions of the emergent group hypothesis (Hérault, 2007) affirmed the removal of functionally equivalent variability, allowing the subsequently built model to only reproduce the stochastic variation in a community’s composition that is relevant to its functioning.

The second step involved the study of associations of biological traits with environmental variables and with each other, seeking support for ecological theories that would allow the definition of functional relationships in the marine benthos (Alexandridis et al., 2017b). Each of the community assembly mechanisms that are represented by these relationships encompasses a variety of processes that could potentially be modeled in more detail. The level of representation was dictated by the available trait, environmental and theoretical knowledge. Hence, biological traits were used as proxies for the role of functional groups in a set of theoretically anticipated community assembly mechanisms.

Ideally, the rules of interaction in IBMs are formulated in terms of fitness maximization, providing a representation that is more general and reliable than empirical formulations (Stillman et al., 2015). In cases where entire communities and their complex webs of interactions need to be represented, IBMs have had to settle for more implicit representations of this first principle. Our rules of interaction are partly phenomenological, but they represent well-established ecological theories that use fitness maximization as their basis. Furthermore, the algorithmic representation of interactions allowed the incorporation of expert knowledge, which is widely available but often difficult to formulate mathematically.

### Inter-scale modeling

The transfer of knowledge from the level of individual organisms, where most observations and experiments are performed, to the level at which biodiversity patterns are typically observed is one of the central problems in ecology (Denny & Benedetti-Cecchi, 2012). The nonparametric up-scaling approach of Cipriotti et al. (2015) is an example of how this issue can be addressed in IBMs. The important state variables of the fine-scale model define a state space, which is divided into a finite number of discrete states. Simulation runs of this model, covering a range of pertinent initial conditions, states and drivers, define transition matrices that are used by the coarse-scale model. The resulting up-scaling is not dynamic and it is restricted to the range of the simulations. Mumby & Dytham (2006) opted, instead, for the development of two dynamically linked, spatially structured models. Computational limitations imposed a mismatch between the two scales, which was bridged by using a toroidal lattice with no boundaries and a mean representation of some processes.

The representation of the dynamic link between processes that operate at different spatial scales is important for the reproduction of the main consequence of cross-scale interactions, namely nonlinear dynamics with threshold values (Peters, Bestelmeyer & Turner, 2007). This study identified transfer processes and spatial heterogeneity at intermediate scales as important components of the link between fine- and coarse-scale patterns and processes. Similarly to this conclusion, the model presented here consists of two spatially distinct representations that are linked by larval dispersal and cell variability. The cells correspond to large ecosystem areas and each of them is represented by a community of much smaller surface. The link between community and ecosystem is implemented through aggregate community measures fed into the ecosystem model, whose output in turn influences community assembly parameters.

### Emerging patterns

The output of the fine-scale model can be considered to represent α-diversity. Diversity at this scale is maintained as a result of inter-group competitive trade-offs and intra-group inhibition. Pre-emptive competition for space is represented explicitly, while exploitative competition for food is implied in the overgrowth competition. The trait of body size is central to the definition of each group’s role in competition, additionally controlling predatory interactions and post-settlement mortality. The role of epibiosis and sediment engineering is represented through the modification of settlement probabilities, which are initially defined by a combination of reproduction-related traits. The generated abundance patterns were characterized by the gradual dominance of a few groups, through a process that represents competitive exclusion.

The turnover of group abundances in the coarse-scale model can be considered to represent β-diversity. Abundance patterns at this level were shaped by diversity among the cells and the process of local dispersal. Inter-cell diversity was driven by the distinction between the subtidal and the intertidal zone and the random initial dominance of stabilizers or destabilizers in each cell. The few areas of destabilizer-dominated cells that were left after 10 years of simulation did not seem able to resist the general trend of cells becoming dominated by sediment stabilizers. This trend could be the result of allowing the system to evolve undisturbed and/or due to overestimating the effect of sediment engineering, particularly sediment stabilization, which might be contingent on groups reaching certain density levels (Posey, 1987).

The model was able to generate levels of β-diversity that were not far from those observed in the Rance estuary in 1995. Exploratory longer simulations of the model indicate that it would probably fail at sustaining high diversity levels, mostly due to the eventual dominance of sediment stabilizers. Local dispersal and sediment engineering were indicated as major drivers of β-diversity. They played this role by facilitating the creation of clusters of cells that were dominated by sediment stabilizers or destabilizers. Early post-settlement mortality also contributed to the maintenance of high levels of β-diversity, but its impact on community composition appeared to be more profound. It prevented small functional groups from dominating and restricted total group abundances to realistic levels. It can probably be largely attributed to adult–juvenile interactions and predation (Desroy, Retière & Thiébaut, 1998).

The gradual elimination of the three epibiotic groups could indicate that epibiosis cannot by itself sustain observed levels of epifaunal diversity, calling for the inclusion of hard substrate types. However, the resulting rareness of groups is also a feature of the observations, which disappears from the model output only when these groups are eliminated. This discrepancy between observations and the final years of the simulation could be due to the concurrent development of cells in the model. Assemblages that are at different successional stages should sustain group rareness and be a more realistic representation of a system that is subject to frequent perturbations, such as wave action or extreme cold (Desroy, 1998). Marine benthic systems are known to demonstrate such transient dynamics (Beukema, Essink & Dekker, 2000).

Some associations of group distribution in the observations could also be seen in the model output, indicating the adequate representation of certain community assembly mechanisms, such as environmental filtering caused by tidal zonation and trophic interactions. It is not clear whether the similarity of observations with patterns produced by the model due to biogenic habitat modification reflects a good depiction of this phenomenon or just mimicry of environmental filtering caused by substrate type. The same holds true for patterns of spatial correlation. Compositional similarity at small distances and dissimilarity at intermediate distances emerged early in the simulation, due to tidal zonation. Compositional dissimilarity at large distances, on the other hand, emerged only toward the end of the simulation, probably due to the formation of clusters of synchronized cells. Similar patterns have been observed in other estuarine macroinvertebrate systems, where they were also attributed to synchronous community development, driven by local dispersal (Ysebaert & Herman, 2002). However, the role of substrate, especially in interaction with biogenic habitat modification, is not clear.

### Future improvements

It appears that the potential of the model to explore the assembly mechanisms of benthic communities could be drastically increased through a few extensions. The combination of sediment engineering with a distinction between different substrate types could allow the model to sustain high diversity levels. This task would be facilitated by the addition of a realistic disturbance regime, which would keep the model away from equilibrium and promote the persistence of rare groups. The explicit representation of these processes would help disentangle the role of substrate type, habitat modification and physical disturbance in shaping marine benthos. The model might also benefit from an improved formulation of trophic interactions. A more detailed description of trophic strategies could be combined with known links between community structure and organic fluxes (Herman et al., 1999), toward the definition of individual-based interactions. Information on primary production would help define organic fluxes, while its combination with hydrodynamics could extend competition to larger distances (Fréchette, Butman & Geyer, 1989). The latter extension could additionally allow for a more explicit representation of larval dispersal, based on the groups’ reproductive trait values.

Any understanding of community assembly mechanisms that is gained by this and future versions of the model should eventually allow its transition to a predictive modeling tool, which could improve projections of benthic biodiversity responses to environmental change. The impact of change could be readily predicted by the model for certain of its aspects, while others might require modifications to the model’s formulation. For instance, the response of benthic communities to changes in the tidal zonation of the Rance estuary due to alternative operation schemes of the tidal power station could be modeled thanks to the inclusion of the environmental filtering effect of tidal zonation in the functional groups’ traits. The same holds true for the response of benthic communities to expected additions of invasive species with certain combinations of the available traits. The responses of benthic biodiversity to increased temperatures or ocean acidification would, on the other hand, require an extension of the model’s functional scope, in order to incorporate these aspects of environmental change. Ultimately, the use of the model for predictive purposes would require its precise parameterization and validation through context-oriented approaches (Kubicek et al., 2015).

Modeling community dynamics largely owes its value to the well-established but poorly understood link between biodiversity and ecosystem functioning (Srivastava & Vellend, 2005). The study of this link requires biodiversity patterns to be explicitly associated with ecosystem functions, such as energy and elemental cycling, the provision of habitat or the modification of physical properties of the system (Frid et al., 2008). These functions have long been the subject of statistical analyses based on many of the biological traits that were here assumed to represent the organisms’ role in community assembly mechanisms (Bremner, Paramor & Frid, 2006). The role of these traits can be extended to that of indicators of ecosystem functioning, in order to associate the latter with modeled biodiversity. This task can be assisted by theoretical (Brown et al., 2004) or empirical (Brey, 2010) links between traits and ecosystem functions.

## Conclusions

Our mechanistic model of the processes that shape estuarine benthos at different spatial scales incorporates novel approaches to the ecological aggregation of communities and the formulation of functional relationships, filling existing gaps in benthic biodiversity modeling. The model reached levels of α- and β-diversity that were similar to those observed in the Rance estuary and indicated local dispersal and high early post-settlement mortality as key processes shaping estuarine benthos. The former allowed the separation of areas with synchronous community composition and the latter kept the abundances of benthic macroinvertebrates well below the levels expected on the basis of resources availability. Environmental filtering due to tidal zonation and prey–predator interactions were represented sufficiently well, while biogenic habitat modification and its interaction with substrate type would benefit from additional research and a more realistic representation. The model’s realism could be further improved through the inclusion of perturbation and a more detailed formulation of benthic trophic strategies. Ultimately, the main challenges of this modeling framework will consist in improving projections of biodiversity responses to environmental change and investigating the links between benthic biodiversity and ecosystem functioning.

## Supplemental Information

10.7717/peerj.5038/supp-1 Supplemental Information 1 Data and code. List of species and their classification. ODD description of the fine-scale model. Observed functional group abundances. Source code of the NetLogo models. Model output of the benchmark simulation.

10.7717/peerj.5038/supp-2 Supplemental Information 2 Code and data used to run the models. Models subtidal and intertidal are the fine-scale models of the subtidal and the intertidal zone, respectively. Model rance is the coarse-scale model. The folders sed and ls contain the GIS substrate data and the LevelSpace extension, respectively.

## Additional Information and Declarations

### Data Availability

The following information was supplied regarding data availability: The raw data and code are provided as Supplemental Files.
