# Supplementary material for: Individual-based simulation of the spatial and temporal dynamics of macroinvertebrate functional groups provides insights into benthic community assembly mechanisms
Source: PeerJ. 2018 Jun 18;6:e5038. doi: 10.7717/peerj.5038 (PMC6011875; doi:10.7717/peerj.5038)
Supplement: Supplemental Information 1 — List of species and their classification. ODD description of the fine-scale model. Observed functional group abundances. Source code of the NetLogo models. Model output of the benchmark simulation. [file peerj-06-5038-s001.doc]

**Supplemental Information**

**S.1 Observed benthic macroinvertebrate species**

**Table A** List of species observed in the Rance estuary in 1995 (for details, see Desroy (1998)), the functional groups to which they belong (for details, see Alexandridis et al. (2017a)) and their classification into phyla, classes, orders and families

| **Species** | **Functional Group** | **Phylum** | **Class** | **Order** | **Family** |
| --- | --- | --- | --- | --- | --- |
| *Abludomelita gladiosa* | FG4 | Arthropoda | Malacostraca | Amphipoda | Melitidae |
| *Abludomelita obtusata* | FG4 | Arthropoda | Malacostraca | Amphipoda | Melitidae |
| *Abra alba* | FG7 | Mollusca | Bivalvia | Veneroida | Semelidae |
| *Abra nitida* | FG7 | Mollusca | Bivalvia | Veneroida | Semelidae |
| *Abra prismatica* | FG7 | Mollusca | Bivalvia | Veneroida | Semelidae |
| *Abra tenuis* | FG7 | Mollusca | Bivalvia | Veneroida | Semelidae |
| *Acanthocardia tuberculata* | FG8 | Mollusca | Bivalvia | Veneroida | Cardiidae |
| *Acanthochitona discrepans* | FG16 | Mollusca | Polyplacophora | Chitonida | Acanthochitonidae |
| *Achelia echinata* | FGP1 | Arthropoda | Pycnogonida | Pantopoda | Ammotheidae |
| *Acrocnida brachiata* | FG8 | Echinodermata | Ophiuroidea | Ophiurida | Amphiuridae |
| *Ampelisca brevicornis* | FG5 | Arthropoda | Malacostraca | Amphipoda | Ampeliscidae |
| *Ampelisca diadema* | FG5 | Arthropoda | Malacostraca | Amphipoda | Ampeliscidae |
| *Ampelisca tenuicornis* | FG4 | Arthropoda | Malacostraca | Amphipoda | Ampeliscidae |
| *Ampelisca typica* | FG4 | Arthropoda | Malacostraca | Amphipoda | Ampeliscidae |
| *Ampharete acutifrons* | FG11 | Annelida | Polychaeta | Terebellida | Ampharetidae |
| *Amphicteis gunneri* | FG10 | Annelida | Polychaeta | Terebellida | Ampharetidae |
| *Amphiglena mediterranea* | FG10 | Annelida | Polychaeta | Sabellida | Sabellidae |
| *Amphilochus spencebatei* | FG4 | Arthropoda | Malacostraca | Amphipoda | Amphilochidae |
| *Amphipholis squamata* | FG2 | Echinodermata | Ophiuroidea | Ophiurida | Amphiuridae |
| *Anapagurus hyndmanni* | FG15 | Arthropoda | Malacostraca | Decapoda | Paguridae |
| *Animoceradocus semiserratus* | FG4 | Arthropoda | Malacostraca | Amphipoda | Maeridae |
| *Antalis vulgaris* | FGP2 | Mollusca | Scaphopoda | Dentaliida | Dentaliidae |
| *Aonides oxycephala* | FG5 | Annelida | Polychaeta | Spionida | Spionidae |
| *Aora typica* | FG5 | Arthropoda | Malacostraca | Amphipoda | Aoridae |
| *Aphelochaeta marioni* | FG11 | Annelida | Polychaeta | Terebellida | Cirratulidae |
| *Apherusa bispinosa* | FG12 | Arthropoda | Malacostraca | Amphipoda | Calliopiidae |
| *Apherusa bispinosa* | FGP2 | Arthropoda | Malacostraca | Amphipoda | Calliopiidae |
| *Aponuphis bilineata* | FG10 | Annelida | Polychaeta | Eunicida | Onuphidae |
| *Apseudopsis latreillii* | FG5 | Arthropoda | Malacostraca | Tanaidacea | Apseudidae |
| *Arenicola marina* | FG11 | Annelida | Polychaeta | Capitellidae | Arenicolidae |
| *Aricidea (Acmira) cerrutii* | FG11 | Annelida | Polychaeta | Cirratulida | Paraonidae |
| *Astacilla longicornis* | FG4 | Arthropoda | Malacostraca | Isopoda | Arcturidae |
| *Balanus crenatus* | FG13 | Arthropoda | Maxillopoda | Sessilia | Balanidae |
| *Bela nebula* | FGP3 | Mollusca | Gastropoda | Neogastropoda | Mangeliidae |
| *Calliostoma zizyphinum* | FG6 | Mollusca | Gastropoda | Vetigastropoda | Calliostomatidae |
| *Callipallene emaciata* | FGP1 | Arthropoda | Pycnogonida | Pantopoda | Pallenidae |
| *Calyptraea chinensis* | FG16 | Mollusca | Gastropoda | Littorinimorpha | Calyptraeidae |
| *Capitella capitata* | FG11 | Annelida | Polychaeta | Capitellida | Capitellidae |
| *Carcinus maenas* | FGP1 | Arthropoda | Malacostraca | Decapoda | Portunidae |
| *Caulleriella alata* | FG5 | Annelida | Polychaeta | Terebellida | Cirratulidae |
| *Cerastoderma edule* | FG8 | Mollusca | Bivalvia | Veneroida | Cardiidae |
| *Cerastoderma glaucum* | FG8 | Mollusca | Bivalvia | Veneroida | Cardiidae |
| *Cereus pedunculatus* | FG16 | Cnidaria | Anthozoa | Actiniaria | Sagartiidae |
| *Cerianthus lloydii* | FGP3 | Cnidaria | Anthozoa | Ceriantharia | Cerianthidae |
| *Chaetozone setosa* | FG11 | Annelida | Polychaeta | Terebellida | Cirratulidae |
| *Cheirocratus intermedius* | FG4 | Arthropoda | Malacostraca | Amphipoda | Cheirocratidae |
| *Cheirocratus sundevalli* | FG4 | Arthropoda | Malacostraca | Amphipoda | Cheirocratidae |
| *Cirratulus cirratus* | FG11 | Annelida | Polychaeta | Terebellida | Cirratulidae |
| *Cirriformia tentaculata* | FG7 | Annelida | Polychaeta | Terebellida | Cirratulidae |
| *Corophium volutator* | FG4 | Arthropoda | Malacostraca | Amphipoda | Corophiidae |
| *Crangon crangon* | FGP3 | Arthropoda | Malacostraca | Decapoda | Crangonidae |
| *Crepidula fornicata* | FG9 | Mollusca | Gastropoda | Littorinimorpha | Calyptraeidae |
| *Cyathura carinata* | FG12 | Arthropoda | Malacostraca | Isopoda | Anthuridae |
| *Cymodoce truncata* | FG12 | Arthropoda | Malacostraca | Isopoda | Sphaeromatidae |
| *Deflexilodes tuberculatus* | FG4 | Arthropoda | Malacostraca | Amphipoda | Oedicerotidae |
| *Dendrodoa grossularia* | FG14 | Chordata | Ascidiacea | Stolidobranchia | Styelidae |
| *Dexamine spinosa* | FG4 | Arthropoda | Malacostraca | Amphipoda | Dexaminidae |
| *Diastylis bradyi* | FG4 | Arthropoda | Malacostraca | Cumacea | Diastylidae |
| *Diodora graeca* | FG16 | Mollusca | Gastropoda | Vetigastropoda | Fissurellidae |
| *Diplocirrus glaucus* | FG7 | Annelida | Polychaeta | Terebellida | Flabelligeridae |
| *Dodecaceria concharum* | FG9 | Annelida | Polychaeta | Terebellida | Cirratulidae |
| *Ebalia tuberosa* | FGP3 | Arthropoda | Malacostraca | Decapoda | Leucosiidae |
| *Edwardsia claparedii* | FGP3 | Cnidaria | Anthozoa | Actiniaria | Edwardsiidae |
| *Ericthonius punctatus* | FG5 | Arthropoda | Malacostraca | Amphipoda | Ischyroceridae |
| *Eteone longa* | FGP3 | Annelida | Polychaeta | Phyllodocida | Phyllodocidae |
| *Euclymene oerstedi* | FG5 | Annelida | Polychaeta | Capitellida | Maldanidae |
| *Eudorella truncatula* | FG4 | Arthropoda | Malacostraca | Cumacea | Leuconidae |
| *Eulalia viridis* | FGP1 | Annelida | Polychaeta | Phyllodocida | Phyllodocidae |
| *Eumida sanguinea* | FGP3 | Annelida | Polychaeta | Phyllodocida | Phyllodocidae |
| *Eunereis longissima* | FGP3 | Annelida | Polychaeta | Phyllodocida | Nereididae |
| *Eupolymnia nebulosa* | FG10 | Annelida | Polychaeta | Terebellida | Terebellidae |
| *Eurysyllis tuberculata* | FG10 | Annelida | Polychaeta | Phyllodocida | Syllidae |
| *Eusyllis blomstrandi* | FGP3 | Annelida | Polychaeta | Phyllodocida | Syllidae |
| *Exogone (Exogone) naidina* | FGP1 | Annelida | Polychaeta | Phyllodocida | Syllidae |
| *Galathea intermedia* | FG12 | Arthropoda | Malacostraca | Decapoda | Galatheidae |
| *Galathowenia oculata* | FG15 | Annelida | Polychaeta | Sabellida | Oweniidae |
| *Gammarus locusta* | FG10 | Arthropoda | Malacostraca | Amphipoda | Gammaridae |
| *Gammarus salinus* | FG4 | Arthropoda | Malacostraca | Amphipoda | Gammaridae |
| *Gibbula magus* | FG4 | Mollusca | Gastropoda | Vetigastropoda | Trochidae |
| *Glycera alba* | FG6 | Annelida | Polychaeta | Phyllodocida | Glyceridae |
| *Glycera tridactyla* | FGP3 | Annelida | Polychaeta | Phyllodocida | Glyceridae |
| *Glycymeris glycymeris* | FGP3 | Mollusca | Bivalvia | Arcoida | Glycymerididae |
| *Golfingia (Golfingia) vulgaris vulgaris* | FG6 | Sipuncula | Sipunculidea | Golfingiida | Golfingiidae |
| *Goniada emerita* | FG3 | Annelida | Polychaeta | Phyllodocida | Goniadidae |
| *Goodallia triangularis* | FGP3 | Mollusca | Bivalvia | Carditoida | Astartidae |
| *Haminoea navicula* | FG8 | Mollusca | Gastropoda | Cephalaspidea | Haminoeidae |
| *Haplosyllis spongicola* | FGP3 | Annelida | Polychaeta | Phyllodocida | Syllidae |
| *Hediste diversicolor* | FGP1 | Annelida | Polychaeta | Phyllodocida | Nereididae |
| *Hesionura elongata* | FG11 | Annelida | Polychaeta | Phyllodocida | Phyllodocidae |
| *Heteromastus filiformis* | FG1 | Annelida | Polychaeta | Capitellida | Capitellidae |
| *Hilbigneris gracilis* | FG10 | Annelida | Polychaeta | Eunicida | Lumbrineridae |
| Holothuriidae | FGP2 | Echinodermata | Holothuroidea | Aspidochirotida | Holothuridae |
| *Idotea granulosa* | FG7 | Arthropoda | Malacostraca | Isopoda | Idoteidae |
| *Iphimedia obesa* | FG14 | Arthropoda | Malacostraca | Amphipoda | Iphimediidae |
| *Iphinoe tenella* | FGP1 | Arthropoda | Malacostraca | Cumacea | Bodotriidae |
| *Janira maculosa* | FG4 | Arthropoda | Malacostraca | Isopoda | Janiridae |
| *Jasmineira elegans* | FG14 | Annelida | Polychaeta | Sabellida | Sabellidae |
| *Jassa falcata* | FG14 | Arthropoda | Malacostraca | Amphipoda | Ischyroceridae |
| *Kurtiella bidentata* | FG14 | Mollusca | Bivalvia | Veneroida | Montacutidae |
| *Lanice conchilega* | FG8 | Annelida | Polychaeta | Terebellida | Terebellidae |
| *Leiochone leiopygos* | FG10 | Annelida | Polychaeta | Capitellida | Maldanidae |
| *Leonnates glauca* | FG10 | Annelida | Polychaeta | Phyllodocida | Nereididae |
| *Lepidochitona (Lepidochitona) cinerea* | FG10 | Mollusca | Polyplacophora | Chitonida | Lepidochitonidae |
| *Lepidonotus squamatus* | FG16 | Annelida | Polychaeta | Phyllodocida | Polynoidae |
| *Leptocheirus hirsutimanus* | FGP3 | Arthropoda | Malacostraca | Amphipoda | Corophiidae |
| *Leptochelia dubia* | FG4 | Arthropoda | Malacostraca | Tanaidacea | Leptocheliidae |
| *Leucothoe incisa* | FG5 | Arthropoda | Malacostraca | Amphipoda | Leucothoidae |
| *Leucothoe spinicarpa* | FG14 | Arthropoda | Malacostraca | Amphipoda | Leucothoidae |
| *Liljeborgia pallida* | FG14 | Arthropoda | Malacostraca | Amphipoda | Liljeborgiidae |
| *Liocarcinus arcuatus* | FG5 | Arthropoda | Malacostraca | Decapoda | Polybiidae |
| *Lucinoma borealis* | FGP3 | Mollusca | Bivalvia | Lucinoida | Lucinidae |
| *Lumbrineris japonica* | FG8 | Annelida | Polychaeta | Eunicida | Lumbrineridae |
| *Lyonsia norwegica* | FGP2 | Mollusca | Bivalvia | Anomalodesmata | Lyonsiidae |
| *Lysianassa ceratina* | FG8 | Arthropoda | Malacostraca | Amphipoda | Lysianassidae |
| *Lysianassa insperata* | FGP1 | Arthropoda | Malacostraca | Amphipoda | Lysianassidae |
| *Lysidice ninetta* | FGP1 | Annelida | Polychaeta | Eunicida | Eunicidae |
| *Lysidice unicornis* | FGP2 | Annelida | Polychaeta | Eunicida | Eunicidae |
| *Macoma balthica* | FGP2 | Mollusca | Bivalvia | Veneroida | Tellinidae |
| *Macropodia rostrata* | FG8 | Arthropoda | Malacostraca | Decapoda | Inachidae |
| *Maera grossimana* | FGP1 | Arthropoda | Malacostraca | Amphipoda | Maeridae |
| *Malacoceros fuliginosus* | FG5 | Annelida | Polychaeta | Spionida | Spionidae |
| *Malmgreniella ljungmani* | FG7 | Annelida | Polychaeta | Phyllodocida | Polynoidae |
| *Malmgreniella lunulata* | FGP1 | Annelida | Polychaeta | Phyllodocida | Polynoidae |
| *Manayunkia aestuarina* | FGP1 | Annelida | Polychaeta | Sabellida | Fabriciidae |
| *Marphysa bellii* | FG10 | Annelida | Polychaeta | Eunicida | Eunicidae |
| *Marphysa sanguinea* | FGP2 | Annelida | Polychaeta | Eunicida | Eunicidae |
| *Mediomastus fragilis* | FGP2 | Annelida | Polychaeta | Capitellida | Capitellidae |
| *Megalomma vesiculosum* | FG3 | Annelida | Polychaeta | Sabellida | Sabellidae |
| *Melinna palmata* | FG5 | Annelida | Polychaeta | Terebellida | Ampharetidae |
| *Microdeutopus anomalus* | FG5 | Arthropoda | Malacostraca | Amphipoda | Aoridae |
| *Microdeutopus damnoniensis* | FG5 | Arthropoda | Malacostraca | Amphipoda | Aoridae |
| *Microdeutopus gryllotalpa* | FG5 | Arthropoda | Malacostraca | Amphipoda | Aoridae |
| *Microdeutopus versiculatus* | FG5 | Arthropoda | Malacostraca | Amphipoda | Aoridae |
| *Mimachlamys varia* | FG5 | Mollusca | Bivalvia | Pectinoida | Pectinidae |
| *Monocorophium acherusicum* | FG9 | Arthropoda | Malacostraca | Amphipoda | Corophiidae |
| *Monocorophium sextonae* | FG5 | Arthropoda | Malacostraca | Amphipoda | Corophiidae |
| *Monoculodes carinatus* | FG5 | Arthropoda | Malacostraca | Amphipoda | Oedicerotidae |
| *Morchellium argus* | FG12 | Chordata | Ascidiacea | Aplousobranchia | Polyclinidae |
| Munnidae | FG14 | Arthropoda | Malacostraca | Isopoda | Munnidae |
| *Myrianida edwardsi* | FG4 | Annelida | Polychaeta | Phyllodocida | Syllidae |
| Mysidae | FG1 | Arthropoda | Malacostraca | Mysida | Mysidae |
| *Mytilus edulis* | FG4 | Mollusca | Bivalvia | Mytiloida | Mytilidae |
| *Myxicola infundibulum* | FG9 | Annelida | Polychaeta | Sabellida | Sabellidae |
| *Nassarius pygmaeus* | FG10 | Mollusca | Gastropoda | Neogastropoda | Nassariidae |
| *Nassarius reticulatus* | FGP3 | Mollusca | Gastropoda | Neogastropoda | Nassariidae |
| Nemertea | FGP3 | Nemertea |  |  |  |
| *Nephtys caeca* | FGP2 | Annelida | Polychaeta | Phyllodocida | Nephtyidae |
| *Nephtys cirrosa* | FGP3 | Annelida | Polychaeta | Phyllodocida | Nephtyidae |
| *Nephtys hombergii* | FGP3 | Annelida | Polychaeta | Phyllodocida | Nephtyidae |
| *Nicolea venustula* | FGP3 | Annelida | Polychaeta | Terebellida | Terebellidae |
| *Notomastus latericeus* | FG10 | Annelida | Polychaeta | Capitellida | Capitellidae |
| *Nototropis vedlomensis* | FG4 | Arthropoda | Malacostraca | Amphipoda | Atylidae |
| *Nucula nucleus* | FG4 | Mollusca | Bivalvia | Nuculida | Nuculidae |
| *Nucula turgida* | FG4 | Mollusca | Bivalvia | Nuculida | Nuculidae |
| *Nudibranchia* | FG4 | Mollusca | Gastropoda | Nudibranchia |  |
| *Nymphon brevirostre* | FGP1 | Arthropoda | Pycnogonida | Pantopoda | Nymphonidae |
| *Odontosyllis ctenostoma* | FGP1 | Annelida | Polychaeta | Phyllodocida | Syllidae |
| *Odontosyllis gibba* | FGP1 | Annelida | Polychaeta | Phyllodocida | Syllidae |
| Oligochaeta | FGP1 | Annelida | Clitellata |  |  |
| *Ophelina acuminata* | FG3 | Annelida | Polychaeta | Opheliida | Opheliidae |
| *Orbinia cuvierii* | FG4 | Annelida | Polychaeta | Orbiniida | Orbiniidae |
| *Oridia armandi* | FG7 | Annelida | Polychaeta | Sabellida | Fabriciidae |
| *Ostrea edulis* | FG14 | Mollusca | Bivalvia | Ostreoida | Ostreidae |
| *Othomaera othonis* | FG9 | Arthropoda | Malacostraca | Amphipoda | Maeridae |
| *Oxydromus flexuosus* | FG4 | Annelida | Polychaeta | Phyllodocida | Hesionidae |
| *Pagurus bernhardus* | FGP3 | Arthropoda | Malacostraca | Decapoda | Paguridae |
| *Palaemon serratus* | FG15 | Arthropoda | Malacostraca | Decapoda | Palaemonidae |
| *Pandora albida* | FGP1 | Mollusca | Bivalvia | Anomalodesmata | Pandoridae |
| *Paradoneis armata* | FG8 | Annelida | Polychaeta | Cirratulida | Paraonidae |
| *Paradoneis lyra* | FG7 | Annelida | Polychaeta | Cirratulida | Paraonidae |
| *Pariambus typicus* | FG7 | Arthropoda | Malacostraca | Amphipoda | Caprellidae |
| *Parvicardium exiguum* | FG4 | Mollusca | Bivalvia | Veneroida | Cardiidae |
| *Parvicardium scabrum* | FG8 | Mollusca | Bivalvia | Veneroida | Cardiidae |
| *Perinereis cultrifera* | FG8 | Annelida | Polychaeta | Phyllodocida | Nereididae |
| *Peringia ulvae* | FGP1 | Mollusca | Gastropoda | Littorinimorpha | Hydrobiidae |
| *Perioculodes longimanus* | FG7 | Arthropoda | Malacostraca | Amphipoda | Oedicerotidae |
| *Phascolion (Phascolion) strombus strombus* | FG4 | Sipuncula | Sipunculidea | Golfingiida | Phascolionidae |
| *Pholoe minuta* | FG4 | Annelida | Polychaeta | Phyllodocida | Pholoidae |
| *Phoronis psammophila* | FGP3 | Phoronida |  |  | Phoronidae |
| *Phtisica marina* | FG5 | Arthropoda | Malacostraca | Amphipoda | Caprellidae |
| *Phyllodoce laminosa* | FGP1 | Annelida | Polychaeta | Phyllodocida | Phyllodocidae |
| *Phyllodoce longipes* | FGP1 | Annelida | Polychaeta | Phyllodocida | Phyllodocidae |
| *Phyllodoce mucosa* | FGP3 | Annelida | Polychaeta | Phyllodocida | Phyllodocidae |
| *Pirakia punctifera* | FGP3 | Annelida | Polychaeta | Phyllodocida | Phyllodocidae |
| *Pisidia longicornis* | FG16 | Arthropoda | Malacostraca | Decapoda | Porcellanidae |
| *Pista cristata* | FG10 | Annelida | Polychaeta | Terebellida | Terebellidae |
| *Platynereis dumerilii* | FGP4 | Annelida | Polychaeta | Phyllodocida | Nereididae |
| *Poecilochaetus serpens* | FG10 | Annelida | Polychaeta | Spionida | Poecilochaetidae |
| *Polititapes aureus* | FG8 | Mollusca | Bivalvia | Veneroida | Veneridae |
| *Polititapes virgineus* | FG8 | Mollusca | Bivalvia | Veneroida | Veneridae |
| *Polycirrus aurantiacus* | FG9 | Annelida | Polychaeta | Terebellida | Terebellidae |
| *Polydora ciliata* | FG13 | Annelida | Polychaeta | Spionida | Spionidae |
| *Pontocrates arenarius* | FG4 | Arthropoda | Malacostraca | Amphipoda | Oedicerotidae |
| *Potamilla torelli* | FG14 | Annelida | Polychaeta | Sabellida | Sabellidae |
| *Prionospio fallax* | FG10 | Annelida | Polychaeta | Spionida | Spionidae |
| *Proceraea aurantiaca* | FGP1 | Annelida | Polychaeta | Phyllodocida | Syllidae |
| *Protodorvillea kefersteini* | FGP3 | Annelida | Polychaeta | Eunicida | Dorvilleidae |
| *Pseudocuma (Pseudocuma) longicorne* | FG4 | Arthropoda | Malacostraca | Cumacea | Pseudocumatidae |
| *Pseudopolydora antennata* | FG10 | Annelida | Polychaeta | Spionida | Spionidae |
| *Pseudopolydora pulchra* | FG10 | Annelida | Polychaeta | Spionida | Spionidae |
| *Pseudoprotella phasma* | FGP1 | Arthropoda | Malacostraca | Amphipoda | Caprellidae |
| *Pygospio elegans* | FG10 | Annelida | Polychaeta | Spionida | Spionidae |
| *Sabella pavonina* | FG16 | Annelida | Polychaeta | Sabellida | Sabellidae |
| *Saccocirrus papillocercus* | FG7 | Annelida | Polychaeta |  | Saccocirridae |
| *Scalibregma celticum* | FG4 | Annelida | Polychaeta | Opheliida | Scalibregmatidae |
| *Scoloplos (Scoloplos) armiger* | FG11 | Annelida | Polychaeta | Orbiniida | Orbiniidae |
| *Scrobicularia plana* | FG8 | Mollusca | Bivalvia | Veneroida | Semelidae |
| *Solen marginatus* | FG8 | Mollusca | Bivalvia | Veneroida | Solenidae |
| *Sphaerosyllis bulbosa* | FG12 | Annelida | Polychaeta | Phyllodocida | Syllidae |
| *Sphaerosyllis hystrix* | FG12 | Annelida | Polychaeta | Phyllodocida | Syllidae |
| *Spio filicornis* | FG10 | Annelida | Polychaeta | Spionida | Spionidae |
| *Spio martinensis* | FG10 | Annelida | Polychaeta | Spionida | Spionidae |
| *Spirobranchus lamarcki* | FG9 | Annelida | Polychaeta | Sabellida | Serpulidae |
| *Spisula elliptica* | FG8 | Mollusca | Bivalvia | Veneroida | Mactridae |
| *Spisula solida* | FG8 | Mollusca | Bivalvia | Veneroida | Mactridae |
| *Sthenelais boa* | FGP3 | Annelida | Polychaeta | Phyllodocida | Sigalionidae |
| *Streblospio shrubsolii* | FG10 | Annelida | Polychaeta | Spionida | Spionidae |
| *Streptodonta pterochaeta* | FG1 | Annelida | Polychaeta | Phyllodocida | Syllidae |
| *Syllides longocirratus* | FG1 | Annelida | Polychaeta | Phyllodocida | Syllidae |
| *Syllidia armata* | FG1 | Annelida | Polychaeta | Phyllodocida | Hesionidae |
| *Syllis armillaris* | FGP1 | Annelida | Polychaeta | Phyllodocida | Syllidae |
| *Syllis cornuta* | FGP1 | Annelida | Polychaeta | Phyllodocida | Syllidae |
| *Syllis gracilis* | FGP1 | Annelida | Polychaeta | Phyllodocida | Syllidae |
| *Syllis hyalina* | FGP1 | Annelida | Polychaeta | Phyllodocida | Syllidae |
| *Syllis prolifera* | FGP1 | Annelida | Polychaeta | Phyllodocida | Syllidae |
| *Syllis variegata* | FGP1 | Annelida | Polychaeta | Phyllodocida | Syllidae |
| *Tectura virginea* | FG16 | Mollusca | Gastropoda | Docoglossa | Acmaeidae |
| *Terebellides stroemii* | FG10 | Annelida | Polychaeta | Terebellida | Trichobranchidae |
| *Tharyx* sp. | FG11 | Annelida | Polychaeta | Terebellida | Cirratulidae |
| *Thia scutellata* | FGP3 | Arthropoda | Malacostraca | Decapoda | Thiidae |
| *Thyasira flexuosa* | FG2 | Mollusca | Bivalvia | Lucinoida | Thyasiridae |
| *Tricolia pullus* | FG16 | Mollusca | Gastropoda | Vetigastropoda | Phasianellidae |
| *Trypanosyllis (Trypanosyllis) coeliaca* | FGP1 | Annelida | Polychaeta | Phyllodocida | Syllidae |
| *Tubulanus polymorphus* | FGP2 | Nemertea | Palaeonemertea | Palaeonemertea | Tubulanidae |
| *Upogebia deltaura* | FG8 | Arthropoda | Malacostraca | Decapoda | Upogebiidae |
| *Urothoe elegans* | FG4 | Arthropoda | Malacostraca | Amphipoda | Urothoidae |
| *Urothoe pulchella* | FG4 | Arthropoda | Malacostraca | Amphipoda | Urothoidae |
| *Urticina felina* | FGP4 | Cnidaria | Anthozoa | Actiniaria | Actiniidae |
| *Venerupis corrugata* | FG8 | Mollusca | Bivalvia | Veneroida | Veneridae |
| *Venus verrucosa* | FG8 | Mollusca | Bivalvia | Veneroida | Veneridae |

**S.2 ODD description of the fine-scale model**

**S.2.1 Purpose**

The model was designed to reproduce the abundance distribution of functional groups within benthic macroinvertebrate communities, using simple rules related to environmental filtering, inter- and intra-group competition for food and space, predation, sediment engineering and epibiosis. It is in this sense a model of α-diversity and a general framework that can include additional community assembly mechanisms and a quantitatively more realistic representation of benthic systems.

**S.2.2 Entities, state variables and scales**

The only entities are cells that make up a square grid with dimensions of 60 × 60 cells. In order to avoid edge effects, the grid wraps horizontally and vertically into a torus. Each cell has two sets of state variables, one for the infauna and one for the epifauna (henceforth *infauna*/*epifauna*).

One cell variable in each set indicates whether a cell is occupied and by a member of which functional group (*sp*/*ep*). There are twenty groups (Table 1). Ten of them belong to the infauna (FG1-8, FG10-11), five groups belong to the epifauna (FG12-16), one can be part of both the infauna and the epifauna (FG9) and four groups represent predator/scavenger organisms (FGP1-4). The infauna consists of four small groups (FG1-4), which can occupy one cell, five intermediate groups (FG5-9), which start at one cell and can occupy its eight immediate neighbours during their growth, and two large groups (FG10-11), which can, through the same procedure, occupy one cell and its twenty-four closest neighbours. The group that can belong to the infauna and the epifauna (FG9) represents basibiotic organisms of intermediate size. It is on cells occupied by this group that epifauna, including individuals of the same group but staying small in size, can settle. In total, the epifauna consists of four small groups (FG9, FG12-14), which can occupy one cell, and two large groups (FG15-16), which start at one cell and can occupy its eight immediate neighbours during their growth.

The other two cell variables of each set indicate the central cells of individuals. The first identifies those among all intermediate and large individuals that grow in spite of competition (*g*/*h*) and the second denotes the age of all individuals that survive at the end of each year (*a*/*ag*).

One global variable for each group of the infauna and the epifauna describes the group’s abundance (*g1*-*16*). One additional variable gives the same number only for the infaunal individuals of the basibiotic group (*ba*). One global variable for each group of the infauna and the epifauna represents the contribution of the group to the respective spawner pool, for use in the recruitment process at the start of each year (*fg1*-*16*). Two global variables give the number of sediment stabilizing and destabilizing individuals (*es*, *ed*), while another one gives the x and y coordinates of the site (*xy*), employed during exporting and importing the model's current status. Five more global variables keep track of the central cells of the individuals of small, intermediate and large infaunal and epifaunal groups that survive at the end of each year (*s*/*sm*, *m*, *l*/*la*). One global variable for each predator/scavenger group describes the group’s abundance (*p1*-*4*).

Cell size corresponds to the theoretical area exclusively occupied by an individual that belongs to one of the small groups. The dimensions of the grid represent an arbitrary sampling area of the real system. One time step corresponds to one year, starting right before an event of spring dispersal.

**S.2.3 Process overview and scheduling**

The model starts by moving time one step forward.

The first of the model's actions represents the process of recruitment. The contribution of each group of the infauna and the epifauna to the respective spawner pool is first defined. Then juveniles of the eleven infaunal groups settle randomly on empty cells. Infaunal juveniles experience post-settlement mortality and those that die are removed from the system. Next, juveniles of the six epifaunal groups settle randomly on cells that are occupied by infaunal adults of the basibiotic group and are empty of epibionts. Epifaunal juveniles experience post-settlement mortality and those that die are removed from the system.

The second action represents the growth in terms of occupied cells of juveniles that belong to groups with intermediate and large size and the process of inter- and intra-group competition for food and space that this entails. First, the juveniles of intermediate infaunal groups, then the infaunal juveniles of the basibiotic group that settled near other group members and finally the juveniles of large infaunal groups grow in random order. The juveniles of the two large epifaunal groups are next to grow, first those of the group that is associated with hard substrate and then those of the group that is associated with gravel.

The third action of the model represents the process of ageing by one year of all individuals that survived the previous time step.

During the fourth and final action, all individuals that could not grow to their full predefined size or were overgrown, those that reached their lifespan during the current time step or were epibionts of deceased basibionts, die and vacate their previously occupied cells. Basibiotic individuals that die of ageing and have epibionts of the same group, take the age of their oldest epibiotic basibiont and retain the rest of their epibionts. Individuals of prey groups die and are removed in decreasing order of their predators' size. This process starts with each predator's most abundant prey and, if this is less abundant than a selected number, individuals from its next most abundant prey are removed, until the total of removed individuals is the closest possible to the selected number.

Finally, all global variables and the model's grid display are updated and functional group abundances are printed out.

**S.2.4 Design concepts**

***Basic principles***

Two versions of the model represent differences in the settlement success of benthic organisms between the subtidal and the intertidal zone. The model represents inter- and intra-group competitive interactions within macroinvertebrate communities, by recreating the life cycle of individuals, from their recruitment as juveniles, to their growth and death. These basic features are further modified through a basic representation of predation and biogenic habitat modification in the form of epibiosis and sediment engineering.

The supply of larvae and the settlement of juveniles are key factors shaping benthic communities, but their complexity often renders simplifications particularly difficult (Pineda et al., 2009). Recruitment processes should, at least partly, depend on the size of spawning populations and the substrate preferences of each organism. They are also expected to be influenced by biological traits such as fecundity, dispersal distance and early development mode, whose values often form combinations indicative of important life history trade-offs (Kupriyanova et al., 2001). The recruitment of juvenile organisms is also significantly impacted by post-settlement mortality, which shows high levels of both intra- and inter-specific variation (Hunt and Scheibling, 1997). Information at the former level indicates that small initial increments in juvenile body size lead to significant increases in survival rates in the face of predation (Gosselin and Qian, 1997).

The successional dynamics of benthic systems have been found to feature space limitation due to adult–juvenile interactions and exploitative competition for food, with the functional role of organisms largely defined by their size (Van Colen et al., 2008). Smaller organisms are expected to show higher growth rates and be better competitors for limited amounts of space, while larger organisms can occupy larger areas and should be competitively superior in the face of food limitation (Alexandridis et al., 2017b). Competitively equivalent individuals also compete for food and space, which should affect their growth rates (Côté et al., 1994). Trophic distinctions, like the one between suspension- and deposit-feeders, can be invalid in view of the highly facultative feeding behaviour of benthic organisms (Snelgrove and Butman, 1994). Trophic interactions could, instead, be dictated by expert knowledge and theoretically anticipated allometries, which should increase the realism and potential for stability of model representations (Brose et al., 2006b).

The majority of predators appear to be larger than their prey and predator size tends to increase with prey size (Cohen et al., 1993). On the other hand, predator–prey body-size ratios are generally the lowest, just over 2 on average, for marine invertebrates, compared to other taxonomic groups and habitat types. It is possible that the energetic costs of prey capture and consumption set a limit to predator–prey size differences (Brose et al., 2006a). In the Rance estuary, fish predation of benthic organisms is restricted and predation by birds is highly seasonal and mostly limited to the intertidal zone. The mortality caused by predatory macroinvertebrates appears likewise to be limited in magnitude, as far as adult prey is concerned. This is due partly to the greater impact of predation on juveniles and partly to the partial ingestion of adults and the regenerative properties of many of them. Predation pressure should depend on the organisms’ position in the sediment and defensive mechanisms along with their relative abundance (Desroy, 1998).

Benthic macroinvertebrates in the Rance estuary appear to differ with regard to their use of space between those that are buried in the sediment and those that occupy its surface supported by hard substrates (Alexandridis et al., 2017b). The dominance of soft bottoms in the estuary indicates that the latter organisms partially occur due to the benefits provided by the phenomenon of epibiosis (Wahl, 1989). Still, sediment engineering is expected to play a much more prominent role in soft bottom systems (Meadows et al., 2012). Its impact on benthic communities can be summarized by the use of the mobility-mode hypothesis, which groups organisms into sediment stabilizers and destabilizers (Posey, 1987). The dominance of the largest members of a group in an area should allow them to modify sediment characteristics in a way that facilitates the settlement of the group’s own members, while inhibiting members of the other group.

***Emergence***

The main output of the model consists of the abundance distribution of functional groups over time. Differences in group abundances can be attributed to the groups’ trophic behaviour, early development mode (T2), dispersal distance (T3), fecundity (T4), preferred substrate (T6), body size (T7), position in the sediment (T9), maximum lifespan (T12) and role in epibiosis (T13) and sediment engineering (T14).

***Adaptation***

Individuals adapt their settlement, growth and lifespan to their environment, namely, the relative contribution of their functional group to the spawner pool, the dominance of sediment stabilizers or destibilizers, the availability of suitable substrate for settlement, other individuals in their potential occupation area, their basibionts or epibionts, the abundance of their group compared to the abundance of potential prey and the abundance of their predator groups.

***Objectives***

The rules of recruitment, competition and mortality are derived from ecological theory and expert knowledge, so objectives are not explicitly modelled. It is, however, implied that the ability to outgrow other individuals and reach the maximum lifespan reflects the fitness of an individual phenotype.

***Prediction***

Because outgrowing other individuals and reaching the maximum lifespan are assumed a priori to be the objectives of each individual, prediction is not modelled.

***Sensing***

There is no sensing in the model. Interactions among individuals are controlled by rules of recruitment, competition and mortality that represent processes of inter- and intra-group competition for food and space, sediment engineering and epibiosis.

***Interaction***

Individuals interact through the explicit representation of pre-emptive competition for space and the implicit representation of exploitative competition for food. Competition for space among infaunal or epifaunal individuals is first demonstrated during the settlement of juveniles on unoccupied cells. Competition for space and food additionally occurs among members of groups with the same size (T7), which prevents them from growing over each other. Competition for the same resources takes place among individuals of different sizes (T7) and involves taking over cells that were already occupied.

Another set of interactions is associated with the phenomenon of epibiosis. Individuals of the basibiotic group provide the cells on which epifaunal individuals can settle. Additionally, the oldest individual of the basibiotic group settled on a basibiont that dies of ageing takes its place. Infaunal basibionts also allow for the growth of juveniles of the same group that settle in their immediate neighbourhood.

Apart from these direct interactions, individuals can interact through their group’s contribution to the infaunal and epifaunal spawner pools (*fg1*-*16*), the relative abundance of sediment stabilizing and destabilizing groups (*es*, *ed*) and the relative abundance of the potential prey groups.

***Stochasticity***

The settlement of juveniles during recruitment is represented by a stochastic process, in which each empty cell is randomly attributed a juvenile that belongs to one of the potential settling groups. Settlement probabilities are determined by each group's early development mode (T2), dispersal distance (T3), fecundity (T4), position in the sediment (T9), role in sediment engineering (T14) and contribution to the spawner pool (*fg1-16*), along with the relative abundance of sediment stabilizing and destabilizing groups (*es*, *ed*) at the previous time step. Post-settlement mortality is also stochastic, as the juveniles to be removed are selected at random, based on each group’s body size (T7). Stochasticity is added to the process of competition through the order in which individuals of the same competitive ability grow. Finally, the individuals to be removed from the system within the preferred prey groups of each predator are also selected at random.

***Collectives***

The functional groups are collectives of individuals that are imposed on the model through the assignment of values to the cell variables of infaunal (*sp*) and epifaunal group occupation (*ep*) during the process of recruitment. The behaviour of members of different groups varies in their initial settlement probabilities, their post-settlement mortality rates, the order and manner in which they grow, their maximum lifespan, their role in the phenomena of sediment engineering and epibiosis and their role as potential prey of the predator groups.

***Observation***

The contribution of each infaunal and epifaunal group to the respective spawner pool (*fg1-16*) is printed out for comparison with the output of the coarse-scale model. Most relevant to the purpose of the model is the abundance distribution of the groups at the end of each time step (*g1-16*). For this to be derived, the total area covered by each group is divided by the number of cells that are occupied by an individual of the group. This is done separately for the infaunal and epifaunal individuals of the basibiotic group, as the two differ in size. The abundance of the former is assigned to a separate variable (*ba*), which is then used to produce the group's total abundance. The abundance of the predator groups is derived from the abundance of their potential prey. Group abundances are printed out at the end of each time step. They are also plotted at the beginning of each time step in the form of a histogram of group abundances and a new entry in a graph that depicts their evolution through time.

**S.2.5 Initialization**

Every cell is randomly attributed a juvenile that belongs to one of the eleven infaunal groups. The chances for a juvenile of each group to be attributed to a cell are defined by the group’s early development mode (T2), dispersal distance (T3) and fecundity (T4). Newly settled juveniles die randomly with chances defined by their group’s body size (T7) and are removed from the system.

Juveniles of obligate infaunal groups with intermediate size (T7) in random order occupy their eight immediate neighbour cells, unless any of them is occupied by a group with the same of size, in which case no cells are occupied. The process is repeated for juveniles of the basibiotic group, on the additional condition that at least one of their twenty-four closest neighbours is occupied by a member of the same group. Juveniles of groups with large size (T7) in random order occupy their twenty-four closest neighbour cells, unless any of them is occupied by a group with the same size, in which case no cells are occupied.

Juveniles of groups with small size (T7) that were not overgrown are attributed to the respective set (*s*) and their age is set to one year (*a*). The same is done for juveniles of groups with intermediate size (T7) that fully grew and were not overgrown (*m*), along with juveniles of groups with large size (T7) that fully grew (*l*). Individuals that did not fully grow or were at least partially overgrown are removed from the system.

Every cell that is occupied by infaunal individuals of the basibiotic group is randomly attributed a juvenile that belongs to one of the six epifaunal groups. The chances for a juvenile of each group to be attributed to a cell are defined by the group’s early development mode (T2), dispersal distance (T3) and fecundity (T4). Newly settled juveniles die randomly with chances defined by their group’s body size (T7) and are removed from the system.

Epifaunal juveniles of groups with large size (T7), first those associated with rock and then those associated with gravel (T6), in random order occupy their eight immediate neighbour cells, unless any of them is occupied by an epifaunal group with the same size, in which case no cells are occupied.

Epifaunal juveniles of groups with small size (T7) that were not overgrown are attributed to the respective set (*sm*) and their age is set to one year (*ag*). The same is done for epifaunal juveniles of groups with large size (T7) that fully grew (*la*). Juveniles of these groups that did not fully grow are removed from the system.

The variables representing the abundance of each functional group (*g1-16*, *ba*) and the abundances of sediment stabilizers and destabilizers (*es*, *ed*) are updated and colours are given to the cells based on their variables of, first, infaunal (*sp*) and then epifaunal group occupation (*ep*).

**S.2.6 Input data**

The model’s abiotic environment is assumed to be constant in both space and time. The contribution of each infaunal and epifaunal group to the respective spawner pool (*fg1-16*) is imported at each time step from the coarse-scale model.

**S.2.7 Submodels**

***Recruitment***

The contribution of each infaunal and epifaunal group to the respective spawner pool (*fg1-16*) is defined as each group’s relative abundance among the respective set of groups at the previous time step within an area defined in the coarse-scale model.

Every cell that is not occupied by an infaunal individual is randomly attributed a juvenile that belongs to one of the eleven infaunal groups. The chances for a juvenile of each group to be attributed to an empty cell are defined by the group’s contribution to the infaunal spawner pool (*fg1-11*), its early development mode (T2), dispersal distance (T3), fecundity (T4), position in the sediment (T9) and role in sediment engineering (T14), along with the relative abundance of sediment stabilizing and destabilizing groups (*es*, *ed*). Newly settled juveniles die randomly with chances defined by their group’s body size (T7) and are removed from the system.

Every cell that is occupied by adult infaunal individuals of the basibiotic group and is empty of epifaunal individuals, is randomly attributed a juvenile that belongs to one of the six epifaunal groups. The chances for a juvenile of each group to be attributed to an empty cell are defined by the group’s contribution to the epifaunal spawner pool (*fg9, fg12-16*), its early development mode (T2), dispersal distance (T3) and fecundity (T4). Newly settled juveniles die randomly with chances defined by their group’s body size (T7) and are removed from the system.

***Competition***

Juveniles of obligate infaunal groups with intermediate size (T7) in random order occupy their eight immediate neighbour cells, unless any of them is occupied by an infaunal group with the same size or adult of a large group, in which case no cells are occupied. The process is repeated for infaunal juveniles of the basibiotic group, on the additional condition that at least one of their twenty-four closest neighbours is occupied by an infaunal adult of the same group. Infaunal juveniles of groups with large size (T7) in random order occupy their twenty-four closest neighbour cells, unless any of them is occupied by an infaunal group with the same size, in which case no cells are occupied.

Epifaunal juveniles of groups with large size (T7), first those associated with rock and then those associated with gravel (T6), in random order occupy their eight immediate neighbour cells, unless any of them is occupied by an epifaunal group with the same size, in which case no cells are occupied.

***Ageing***

One year is added to the age of infaunal (*a*) and epifaunal individuals (*ag*) that survived the previous time step.

***Mortality***

Infaunal juveniles of groups with small size (T7) that were not overgrown are attributed to the respective set (*s*) and their age is set to one year (*a*). The same is done for infaunal juveniles of groups with intermediate size (T7) that fully grew and were not overgrown (*m*), along with infaunal juveniles of groups with large size (T7) that fully grew (*l*). Obligate infaunal individuals that did not fully grow or were at least partially overgrown along with those that reached their lifespan are removed from the system.

Epifaunal juveniles of groups with small size (T7) that were not overgrown are attributed to the respective set (*sm*) and their age is set to one year (*ag*). The same is done for epifaunal juveniles of groups with large size (T7) that fully grew (*la*). Juveniles of these groups that did not fully grow, along with adults that reached their lifespan, are removed from the system. Infaunal individuals of the basibiotic group that reached their lifespan are removed from the system, along with their epibiotic individuals, unless any of the epibionts is member of the basibiotic group. In this case, the oldest among these epibionts becomes infaunal and keeps the rest of the epibionts.

The abundances of predator groups are derived from the abundances of their potential prey groups. The predator group with the largest size (T7) starts with the most abundant group among its potential prey and removes individuals at random, until the selected number has been removed (group abundances as observed at the end of the previous time step). If this number is not reached, it moves to the next group of its potential prey and repeats the process, until no potential prey groups are left. The same is done for the predator groups with the second and third largest sizes (T7).

The variables representing the abundance of each functional group (*g1-16*, *ba*) and the abundances of sediment stabilizers and destabilizers (*es*, *ed*) are updated, colours are given to the cells based on their variables of, first, infaunal (*sp*) and then epifaunal group occupation (*ep*) and the abundances of all groups are printed out.

**S.3 Observed functional group abundances**

Comma-separated values of the abundances of 20 functional groups (FG1-16, FGP1-4) in 113 stations (along with their geographic coordinates) that were sampled in the Rance estuary in 1995 prior to the spring recruitment. Two replicate samples were collected at each of 103 submerged stations using a 0.1 m^2^ Smith Mac-Intyre grab, while 10 emerged stations were sampled using a hand corer (5 replicates; replicate area of 1/55 m^2^) to a depth of 20 cm. Although densities of organisms were extrapolated to a standard surface area, some bias was unavoidably introduced, due to the different characteristics of the sampling gears. All samples were gently washed in situ through a 1 mm sieve and preserved in 4.5% formalin before being sorted, identified and counted in the laboratory. Macroinvertebrates retained on the mesh were determined at species level when possible. A total of 240 species or higher taxonomic groups belonging to 9 phyla were thus identified (for details, see Desroy (1998)). These taxa were classified into 20 functional groups (for details, see Alexandridis et al. (2017a)) and their abundances were pooled within each group and station to generate the following functional group abundances.

Station,Longitude_E,Latitude_N,FG1,FG2,FG3,FG4,FG5,FG6,FG7,FG8,FG9,FG10,FG11,FG12,FG13,FG14,FG15,FG16,FGP1,FGP2,FGP3,FGP4

1,-1.97746,48.5699,1,0,51,7,105,0,1,0,0,1,26,0,0,0,0,5,0,0,64,0

2,-1.97709,48.57,0,0,0,0,41,0,2,0,0,1,19,0,0,0,0,3,0,1,66,0

3,-1.97788,48.5696,0,5,7,6,118,0,8,0,0,0,15,0,0,0,0,1,0,0,61,0

4,-1.97825,48.5695,0,6,2,35,277,0,11,3,0,8,25,0,0,0,0,2,0,0,54,0

5,-1.99155,48.563,0,6,0,169,631,0,9,10,0,117,42,0,14,1,0,0,4,1,49,0

6,-1.99161,48.5695,0,9,8,32,199,0,7,5,1,32,185,1,1,0,0,0,5,5,24,1

7,-1.97934,48.5686,0,30,0,135,506,0,24,2,0,34,87,0,11,0,0,0,0,0,56,2

8,-1.98149,48.5672,0,11,2,44,78,0,51,7,84,63,161,1,5,8,0,10,6,0,35,0

9,-1.9873,48.5658,0,2,9,66,90,0,56,10,4,120,157,2,0,0,0,10,1,6,21,0

10,-1.98811,48.5611,0,20,1,142,590,0,12,11,0,168,44,0,6,2,0,2,22,7,81,0

11,-1.98337,48.563,0,16,0,62,165,1,54,3,19,74,158,1,0,1,2,6,6,18,33,0

12,-1.97912,48.563,0,15,0,41,539,0,16,1,0,42,5,0,0,0,0,0,0,0,72,0

13,-1.98074,48.5592,0,17,1,85,412,0,7,11,0,107,79,0,6,1,0,0,1,12,43,0

14,-1.98574,48.5602,0,14,1,33,470,0,10,5,2,74,127,0,1,0,0,0,4,7,56,0

15,-1.97493,48.5547,0,0,1,0,40,0,6,1,0,92,9,0,12,0,0,0,0,0,2,0

16,-1.98118,48.5532,0,46,0,34,663,0,12,4,1,88,26,0,2,0,0,6,9,13,40,0

17,-1.99118,48.5792,5,4,0,55,57,0,0,5,598,8,1,42,0,3,0,7,167,2,68,0

18,-2.00023,48.5755,0,1,34,5,249,0,6,0,0,41,211,0,0,0,0,0,0,0,71,0

19,-1.9971,48.5738,0,11,1,42,584,0,11,14,0,111,87,0,5,2,0,2,71,1,79,0

20,-1.9987,48.5689,0,3,1,157,333,0,11,6,0,60,50,0,25,0,0,1,2,4,38,0

21,-1.99558,48.5689,0,21,1,70,761,0,12,17,0,157,63,4,16,1,1,1,7,12,60,2

22,-1.98934,48.5708,0,2,14,76,76,1,72,8,27,109,65,0,1,2,0,6,10,21,34,0

23,-1.99558,48.5724,0,3,2,21,355,0,3,18,0,84,664,0,17,1,0,12,32,3,57,0

24,-2.00183,48.5719,0,2,1,1,224,0,5,2,0,58,146,0,3,0,0,3,2,0,66,0

25,-2.00161,48.5744,1,0,476,3,27,0,13,0,0,120,312,39,1,0,0,4,5,0,52,0

26,-1.99861,48.5657,0,0,73,9,151,0,2,0,0,33,127,3,0,0,0,0,48,0,68,0

27,-1.98871,48.5542,0,1,39,1,117,0,2,1,0,13,25,1,0,0,0,0,0,0,91,0

28,-1.97493,48.5655,0,0,5,0,17,0,4,2,0,52,154,1,2,0,0,6,2,1,12,0

29,-1.99333,48.5806,1,2,0,46,140,2,8,4,1,47,178,0,1,2,1,1,6,4,20,0

30,-1.97881,48.5707,0,1,0,3,211,0,1,1,0,9,13,0,0,0,0,2,0,0,52,0

31,-1.97643,48.5689,0,2,0,1,209,0,3,0,0,5,17,0,0,0,0,1,0,0,55,0

32,-1.976,48.5673,0,3,1,5,267,0,3,1,0,87,42,2,1,0,0,25,1,0,55,0

33,-1.97072,48.5259,0,0,2828,0,0,0,571,68,0,109,182,0,0,0,0,16,0,0,14,0

34,-1.97537,48.5243,0,0,572,1,1,0,267,91,0,129,391,0,6,0,0,24,0,0,29,0

35,-1.96116,48.5316,0,0,2473,0,0,0,2374,52,0,389,51,0,1,0,0,10,5,2,13,0

36,-1.96234,48.5362,0,0,259,2,0,0,4,1,0,14,17,0,1,0,0,0,1,0,69,0

37,-1.97525,48.5869,0,3,0,9,269,0,0,1,0,27,11,1,0,4,0,18,25,0,29,0

38,-1.96685,48.5827,0,0,618,3,68,0,186,2,0,175,1112,0,949,3,0,1,7,0,55,0

39,-1.98655,48.5899,0,0,1,4,61,0,2,3,1,36,279,0,93,0,0,4,2,0,11,0

40,-1.97847,48.5476,0,8,1,49,213,0,72,7,9,49,63,0,0,0,0,2,6,3,38,0

41,-1.98096,48.5501,0,22,0,41,203,0,5,9,0,63,42,0,0,4,0,0,5,6,31,0

42,-1.9774,48.551,0,19,1,42,350,0,4,8,0,93,19,0,2,4,0,2,5,8,38,0

43,-1.96912,48.5489,0,0,0,3,115,0,55,2,0,78,67,0,1,0,0,2,1,2,15,0

44,-1.96707,48.5457,0,4,0,7,180,0,4,1,0,15,26,0,1,0,0,2,5,0,33,0

45,-1.97244,48.5343,0,0,0,0,0,0,0,0,0,5,0,0,0,0,0,0,1,0,4,0

46,-1.96772,48.5358,0,0,26,3,50,0,2,2,0,31,33,0,2,0,0,1,0,0,64,0

47,-1.96847,48.5338,0,0,17,1,20,0,28,3,17,31,6,0,1,0,0,3,3,1,4,0

48,-1.96772,48.5325,0,0,1179,0,15,0,109,34,0,456,197,3,7,0,0,1,4,3,40,0

49,-1.97278,48.5322,0,0,0,0,0,0,0,0,0,3,0,1,0,0,0,0,0,0,1,0

50,-1.98246,48.5265,0,0,0,0,1,0,693,43,0,3,6,0,1,0,0,0,0,0,64,0

51,-1.98052,48.5259,0,0,3,0,0,0,0,4,1,2,0,0,0,0,0,1,0,0,3,0

52,-1.97869,48.525,0,0,0,0,1,0,0,0,0,2,2,1,0,0,0,1,0,0,3,0

53,-1.9773,48.5302,0,0,16,1,2,0,1,0,0,8,2,0,0,0,0,5,0,0,28,0

54,-1.97525,48.529,0,0,1,1,1,0,0,0,0,14,0,0,0,0,0,0,0,0,5,0

55,-1.97428,48.5279,0,0,34,15,24,0,11,0,2,12,96,0,1,0,0,17,0,0,36,0

56,-1.98396,48.5192,0,0,2,0,1,0,2,1,0,3,0,0,0,0,0,0,0,0,14,0

57,-1.98256,48.5197,0,0,10,0,47,0,25,1,0,9,4,0,0,0,0,0,1,0,40,0

58,-1.97987,48.5206,0,0,0,2,238,0,0,0,0,14,2,0,0,0,0,0,0,0,73,0

59,-1.97805,48.5214,0,0,54,0,88,0,6,0,0,16,21,1,0,0,0,0,0,0,51,0

60,-1.97772,48.5173,0,0,3,0,0,0,0,3,0,8,1,0,0,0,0,0,0,0,18,0

61,-1.9759,48.5183,0,0,10,0,37,0,89,57,0,24,28,0,0,0,0,0,3,0,31,0

62,-1.97375,48.5197,0,0,8,0,49,0,9,7,0,7,10,0,0,0,0,0,1,0,70,0

63,-1.9774,48.5155,0,0,2,0,2,0,1,0,0,13,0,0,0,0,0,0,0,0,16,0

64,-1.97643,48.5141,0,0,4,1,2,0,0,0,0,3,0,0,0,0,0,0,0,0,3,0

65,-1.97525,48.515,0,0,4,0,34,0,152,8,0,26,26,0,0,23,0,0,0,0,72,0

66,-1.98052,48.513,0,0,0,7,0,0,39,11,0,7,4,0,0,2,0,0,0,0,18,0

67,-1.98321,48.5101,0,0,0,0,0,0,0,1,0,4,0,0,0,0,0,0,0,0,7,0

68,-1.98096,48.5033,0,0,0,0,0,0,20,12,0,15,60,1,0,0,0,0,0,0,4,0

69,-1.98062,48.4995,0,0,15,42,1,0,20,56,0,19,43,4,0,0,0,0,0,1,2,0

70,-1.9858,48.4986,0,0,2,0,0,0,0,0,0,2,7,0,0,0,0,0,0,0,0,0

71,-1.98924,48.4992,0,0,8,1,0,0,3,2,2,21,36,1,0,0,0,0,0,0,0,0

72,-1.99892,48.4949,0,0,39,3,0,0,0,3,0,14,87,0,0,1,0,0,0,0,0,0

73,-1.99452,48.4944,0,0,0,0,0,0,0,0,0,0,22,0,0,1,0,0,0,0,0,0

74,-1.99301,48.4983,0,0,3,2,0,0,0,18,0,13,41,0,0,3,0,0,0,0,0,0

75,-2.0226,48.6,0,1,19,65,80,0,113,5,39,69,48,0,1,1,0,2,10,32,35,1

76,-2.02356,48.6009,0,7,1,32,349,0,10,12,1,209,54,0,6,1,1,2,4,3,29,0

77,-2.02141,48.6011,0,2,0,308,369,0,6,11,1,295,54,0,25,5,2,6,6,12,35,0

78,-2.01141,48.603,0,1,0,9,66,0,2,1,0,36,44,0,0,1,2,1,0,1,9,1

79,-2.01313,48.6032,0,0,27,69,155,0,71,6,35,52,53,2,0,0,2,5,42,23,88,0

80,-2.02044,48.6043,0,0,13,115,172,0,12,13,17,131,36,1,0,6,0,10,6,16,29,0

81,-2.02141,48.6043,0,1,10,32,152,0,17,3,1,32,56,0,1,2,2,5,6,5,36,0

82,-2.02228,48.6043,0,4,4,13,190,0,38,7,2,36,25,0,2,0,0,2,3,4,40,0

83,-2.00861,48.6004,0,3,0,74,220,0,35,6,17,46,32,0,4,1,0,6,7,23,45,1

84,-2.00797,48.5983,3,0,25,13,18,0,5,1,1,6,0,13,0,1,1,4,166,4,150,0

85,-2.0042,48.5978,1,2,0,54,37,2,2,3,20,23,3,4,0,4,0,5,59,7,33,1

86,-2.00366,48.5997,0,0,0,22,41,0,2,5,0,40,71,0,0,1,0,0,1,0,9,0

87,-2.00173,48.5928,3,0,0,4,1,0,10,1,0,1,0,36,0,1,0,1,11,0,1,0

88,-2.01453,48.592,0,7,0,78,494,0,14,8,0,230,20,0,14,1,0,1,7,8,31,0

89,-2.01216,48.6121,0,0,0,2,11,0,6,0,0,1,1,0,0,0,0,0,0,5,2,0

90,-2.02003,48.6121,2,0,2,2,5,1,0,2,18,5,2,88,0,0,1,0,34,3,49,0

91,-2.01173,48.6112,0,0,0,2,28,0,10,1,1,1,10,0,0,0,0,0,0,6,2,0

92,-2.01281,48.6137,0,3,62,0,130,0,4,3,1,32,212,0,0,0,0,3,0,0,41,0

93,-2.01066,48.6138,0,0,216,1,22,0,5,1,0,103,37,1,0,0,0,11,0,0,40,0

94,-2.02206,48.6091,23,2,31,102,176,0,138,10,111,50,56,4,0,5,6,21,43,55,172,0

95,-2.02475,48.6071,0,2,4,16,132,0,4,14,0,32,117,1,0,1,0,0,3,0,26,0

96,-2.02744,48.6163,3,0,0,94,124,0,6,9,43,34,0,4,0,4,10,25,207,5,112,0

97,-2.0226,48.6118,0,0,0,30,27,0,29,0,20,29,16,1,0,2,1,2,5,9,10,0

98,-2.02777,48.6066,0,0,338,39,30,0,423,0,0,1084,296,5,25,19,0,0,8,0,14,0

99,-2.02659,48.6098,0,0,0,29,35,0,3,3,0,15,508,0,15,0,0,0,1,0,3,0

100,-2.02509,48.6104,0,0,1,8,265,0,1,2,0,26,37,0,6,0,0,0,3,2,15,0

101,-2.02819,48.6157,0,1,0,12,39,0,0,1,1,23,19,0,0,1,3,1,1,10,7,0

102,-2.02949,48.6145,0,0,8,8,43,0,2,0,0,11,32,0,1,0,0,18,0,1,1,0

103,-2.02475,48.6135,0,1,62,13,54,0,112,0,622,6,8,1,0,1,10,18,27,6,50,0

104,-2.00742,48.6125,0,0,0,204,4,0,91,2,0,2,279,0,0,0,0,0,4,0,36,0

105,-2.00916,48.6123,0,0,0,0,15,0,82,6,0,2,771,0,0,0,0,0,0,0,45,0

106,-2.00689,48.6107,0,0,0,287,11,0,169,2,0,0,689,0,0,0,0,98,0,2,28,0

107,-2.00807,48.6107,0,0,0,4,22,0,47,7,0,2,508,0,0,0,0,4,2,0,34,0

108,-2.01023,48.6111,0,0,0,2,11,0,2,0,0,2,102,0,0,0,0,0,0,0,8,0

109,-1.96997,48.5872,0,0,5333,764,197,0,80,8,0,67,39,5,61,0,0,0,0,0,23,0

110,-1.96288,48.5792,0,0,815,0,0,0,20,20,0,533,106,0,193,0,0,0,0,0,19,0

111,-1.96987,48.5836,0,0,7,28,214,0,35,27,0,499,802,0,97,0,0,0,0,0,55,0

112,-1.98062,48.5928,0,0,42,0,76,0,78,26,0,307,643,2,38,0,0,42,4,0,22,0

113,-1.97203,48.5175,0,0,1020,0,60,0,262,1398,0,35,155,0,0,0,0,0,0,0,97,0

**S.4 Source code of the NetLogo models**

**S.4.1 Subtidal version of the fine-scale model**

globals [es ed s m l sm la xy n p1 p2 p3 p4 g1 g2 g3 g4 g5 g6 g7 g8 g9 g10 g11 g12 g13 g14 g15 g16 ba fg1 fg2 fg3 fg4 fg5 fg6 fg7 fg8 fg9 fg10 fg11 fg12 fg13 fg14 fg15 fg16] ; define variables of abundances of sediment stabilizing (es) and destabilizing (ed) groups, central patches of the individuals of small (s), intermediate (m), large (l) infaunal and small (sm), intermediate (la) epifaunal groups that survive at the end of each year, IBM name according to x-y coordinates of respective Rance patch (xy), number of functional groups in the system (n), abundances of 4 predatory groups (p1-p4), abundances of 16 infaunal/epifaunal groups (g1-g16), abundance of infaunal individuals of basibiotic group (ba) and contributions of infaunal/epifaunal groups to the respective spawner pool (fg1-fg16)

patches-own [sp ep g h a ag] ; define patch variables of infaunal (sp) and epifaunal (ep) group occupation, central patches of intermediate and large infaunal (g) and intermediate epifaunal (h) individuals that grow in spite of competition and age of infaunal (a) and epifaunal (ag) individuals that survive at the end of a year

to make-movie ; make movie of model interface during initialization plus 9 time steps

user-message "First, save your new movie file (choose a name ending with .mov)"

let path user-new-file

if not is-string? path [ stop ]

setup

movie-start path

movie-set-frame-rate 1

movie-grab-interface

while [ ticks < 10 ]

[go

movie-grab-interface ]

movie-close

user-message (word "Exported movie to " path)

end

to export ; export all model entities and output to an external file named after the value of the xy variable

export-world (word xy ".csv")

end

to import ; import all model entities and output from an external file named after the value of the xy variable

import-world (word xy ".csv")

end

to setup ; initialize the model

clear-all ; clear all model entities and output

set s no-patches ; set variable s to an empty patch agentset

set m no-patches ; set variable m to an empty patch agentset

set l no-patches ; set variable l to an empty patch agentset

set sm no-patches ; set variable sm to an empty patch agentset

set la no-patches ; set variable la to an empty patch agentset

ask patches [set sp one-of (list 45 45 55 65 65 75 75 75 85 85 85 95 95 105 105 105 115 115 115 125 125 125 135 135 135 145 145 145)] ; randomly attribute an infaunal group to the infaunal group occupation variable of each patch/the chances of each group to be attributed are defined by its fecundity, dispersal distance and early development mode

ask n-of ( 0.9 * count patches with [ sp = 45 or sp = 55 or sp = 65 or sp = 75 ] ) patches with [ sp = 45 or sp = 55 or sp = 65 or sp = 75 ] [ set sp 35 ] ; randomly remove a subset of newly settled infaunal juveniles of each group from the system/juvenile mortality rates are defined by the body size of each group

ask n-of ( 0.5 * count patches with [ sp = 85 or sp = 95 or sp = 105 or sp = 115 or sp = 125 ] ) patches with [ sp = 85 or sp = 95 or sp = 105 or sp = 115 or sp = 125 ] [ set sp 35 ]

ask n-of ( 0.1 * count patches with [ sp = 135 or sp = 145 ] ) patches with [ sp = 135 or sp = 145 ] [ set sp 35 ]

ask patches with [sp > 80 and sp < 120] [ ; in random order expand patch occupation of surviving juveniles of purely infaunal intermediate groups to 8 immediate neighbors, unless any of them is occupied by an infaunal individual of the same size

if map [? < 85 or ? > 125] [sp] of neighbors = [true true true true true true true true] [

ask neighbors [set sp [sp] of myself]

set g -3 ; assign the value -3 to the central patches variable of purely infaunal intermediate individuals that grew in spite of competition

]

]

ask patches with [sp = 125] [ ; in random order expand patch occupation of surviving juveniles of the basibiotic group to 8 immediate neighbors, unless any of them is occupied by an infaunal individual of the same size and provided that at least one patch within each patch's 24 closest neighbors is occupied by an individual of the same group

if map [? < 85 or ? > 125] [sp] of neighbors = [true true true true true true true true] and count patches in-radius 2.9 with [sp = 125] > 1 [

ask neighbors [set sp [sp] of myself]

set g -3 ; assign the value -3 to the central patches variable of basibiotic intermediate individuals that grew in spite of competition

]

]

ask patches with [sp > 130] [ ; in random order expand patch occupation of surviving juveniles of infaunal large groups to 24 closest neighbors, unless any of them is occupied by an infaunal individual of the same size

if map [? < 135] [sp] of other patches in-radius 2.9 = [true true true true true true true true true true true true true true true true true true true true true true true true] [

ask patches in-radius 2.9 [set sp [sp] of myself]

set g -5 ; assign the value -5 to the central patches variable of large infaunal individuals that grew in spite of competition

]

]

ask patches with [sp > 40 and sp < 80] [set sp (sp - 35) / 10 set g -11 set a 0] ; assign the value -11 to the central patches variable of small infaunal individuals that were not overgrown and set their age to 1 year

set s (patch-set patches with [g = -11]) ; assign the central patches of small infaunal individuals that were not overgrown to the respective patch set

ask patches with [g = -3 and sp < 130 and length (filter [? = sp] [sp] of patches in-radius 1.9) = 9] [ask patches in-radius 1.9 [set sp (sp - 35) / 10] set g -7 set a 0] ; assign the value -7 to the central patches variable of intermediate infaunal individuals that grew and were not overgrown and set their age to 1 year

set m (patch-set patches with [g = -7]) ; assign the central patches of intermediate infaunal individuals that grew and were not overgrown to the respective patch set

ask patches with [g = -5 and length (filter [? = sp] [sp] of patches in-radius 2.9) = 25] [ask patches in-radius 2.9 [set sp (sp - 35) / 10] set g -9 set a 0] ; assign the value -9 to the central patches variable of large infaunal individuals that grew and were not overgrown and set their age to 1 year

set l (patch-set patches with [g = -9]) ; assign the central patches of large infaunal individuals that grew and were not overgrown to the respective patch set

ask patches with [sp > 11] [set sp 35] ; clear the infaunal group occupation variable of patches occupied by infaunal individuals that did not grow or were overgrown

ask patches with [sp = 9] [set ep one-of (list 165 165 165 175 175 175 185 185 185 195 195 195 205 205 205 215 215)] ; randomly attribute an epifaunal group to the epifaunal group occupation variable of patches occupied by infaunal individuals of the basibiotic group/the chances of each group to be attributed are defined by its fecundity, dispersal distance and early development mode

ask n-of ( 0.9 * count patches with [ ep = 165 or ep = 175 or ep = 185 ] ) patches with [ ep = 165 or ep = 175 or ep = 185 ] [ set ep 0 ] ; randomly remove a subset of newly settled epifaunal juveniles of each group from the system/juvenile mortality rates are defined by the body size of each group

ask n-of ( 0.5 * count patches with [ ep = 195 ] ) patches with [ ep = 195 ] [ set ep 0 ]

ask n-of ( 0.1 * count patches with [ ep = 205 or ep = 215 ] ) patches with [ ep = 205 or ep = 215 ] [ set ep 0 ]

ask patches with [ep = 215] [ ; in random order expand patch occupation of surviving juveniles of epifaunal intermediate groups associated with rock to 8 immediate neighbors, unless any of them is occupied by an epifaunal individual of the same size

if map [? < 200] [ep] of neighbors = [true true true true true true true true] [

ask neighbors [set ep [ep] of myself]

set h 3 ; assign the value 3 to the central patches variable of epifaunal intermediate individuals associated with rock that grew in spite of competition

]

]

ask patches with [ep = 205] [ ; in random order expand patch occupation of surviving juveniles of epifaunal intermediate groups associated with gravel to 8 immediate neighbors, unless any of them is occupied by an epifaunal individual of the same size

if map [? < 200] [ep] of neighbors = [true true true true true true true true] [

ask neighbors [set ep [ep] of myself]

set h 3 ; assign the value 3 to the central patches variable of epifaunal intermediate individuals associated with gravel that grew in spite of competition

]

]

ask patches with [ep > 160 and ep < 200] [set ep (ep - 35) / 10 set h 5 set ag 0] ; assign the value 5 to the central patches variable of small epifaunal individuals that were not overgrown and set their age to 1 year

set sm (patch-set patches with [h = 5]) ; assign the central patches of small epifaunal individuals that were not overgrown to the respective patch set

ask patches with [h = 3 and length (filter [? = ep] [ep] of patches in-radius 1.9) = 9] [ask patches in-radius 1.9 [set ep (ep - 35) / 10] set h 7 set ag 0] ; assign the value 7 to the central patches variable of intermediate epifaunal individuals that grew and were not overgrown and set their age to 1 year

set la (patch-set patches with [h = 7]) ; assign the central patches of intermediate epifaunal individuals that grew and were not overgrown to the respective patch set

ask patches with [ep > 18] [set ep 0] ; clear the epifaunal group occupation variable of patches occupied by epifaunal individuals that did not grow

set g1 count patches with [ sp = 1 ] ; give the small infaunal groups abundance variables values equal to the number of patches occupied by each group

set g2 count patches with [ sp = 2 ]

set g3 count patches with [ sp = 3 ]

set g4 count patches with [ sp = 4 ]

set g5 count patches with [ sp = 5 ] / 9 ; give the intermediate infaunal groups abundance variables values equal to the number of patches occupied by each group divided by the individually occupied number of patches

set g6 count patches with [ sp = 6 ] / 9

set g7 count patches with [ sp = 7 ] / 9

set g8 count patches with [ sp = 8 ] / 9

set g9 count patches with [ sp = 9 ] / 9

set ba g9 ; give the infaunal basibionts abundance variable a value equal to the infaunal abundance of the basibiotic group

set g10 count patches with [ sp = 10 ] / 25 ; give the large infaunal groups abundance variables values equal to the number of patches occupied by each group divided by the individually occupied number of patches

set g11 count patches with [ sp = 11 ] / 25

set p1 round ((g4 + g7) / 10) ; give the predatory groups abundance variables values equal to the sum of their prey groups abundance divided by 10

set p3 round ((g4 + g5) / 10)

set p2 round ((g7 + g11) / 10)

set p4 round ((p1 + p3) / 10)

set es (g5 + g10) ; give the sediment stabilizing groups abundance variable a value equal to the sum of the abundances of intermediate and large sessile stabilizers

set ed (g7 + g11) ; give the sediment destabilizing groups abundance variable a value equal to the sum of the abundances of intermediate and large destabilizers

ask patches [set pcolor sp] ; color patches according to their infaunal group occupation variable

set g12 count patches with [ ep = 13 ] ; give the small epifaunal groups abundance variables values equal to the number of patches occupied by each group

set g13 count patches with [ ep = 14 ]

set g14 count patches with [ ep = 15 ]

set g9 g9 + count patches with [ ep = 16 ] ; give the basibiotic group abundance variable a value equal to the sum of the abundances of the infaunal and epifaunal individuals of the group

set g15 count patches with [ ep = 17 ] / 9 ; give the intermediate epifaunal groups abundance variables values equal to the number of patches occupied by each group divided by the individually occupied number of patches

set g16 count patches with [ ep = 18 ] / 9

ask patches with [ ep > 0 ] [ set pcolor ep ] ; color patches occupied by epifaunal individuals according to their epifaunal group occupation variable

set n length filter [? > 0] (list g1 g2 g3 g4 g5 g6 g7 g8 g9 g10 g11 g12 g13 g14 g15 g16 p1 p2 p3 p4) ; give the variable of number of functional groups in the system a value equal to the number of groups with abundances larger than 0

reset-ticks ; set tick counter to zero, set up and update all plots

end

to go ; move the model one step forward

set-current-plot "Functional group abundance" ; clear the histogram of functional group abundances

clear-plot

tick ; advance the tick counter by one and update all plots

do-recruitment ; run the recruitment submodel

do-competition ; run the competition submodel

do-ageing ; run the ageing submodel

do-mortality ; run the mortality submodel

end

to do-recruitment ; run the recruitment submodel

output-print " " output-type "f " output-type fg1 output-type " " output-type fg2 output-type " " output-type fg3 output-type " " output-type fg4 output-type " " output-type fg5 output-type " " output-type fg6 output-type " " output-type fg7 output-type " " output-type fg8 output-type " " output-type fg9 output-type " " output-type fg10 output-type " " output-type fg11 output-type " " output-type fg12 output-type " " output-type fg13 output-type " " output-type fg14 output-type " " output-type fg15 output-type " " output-print fg16 ; print out the values of infaunal/epifaunal groups contribution to the respective spawner pool

let fg ( fg1 + fg2 + fg3 + fg4 + fg5 + fg6 + fg7 + fg8 + fg9 + fg10 + fg11 ) ; define the contribution of infaunal groups to the respective spawner pool as the contribution of each group to the total abundance of the respective set of groups during the previous time step

ifelse es > ed ; randomly attribute an infaunal group to the infaunal group occupation variable of patches that are not occupied by infauna/the chances of each group to be attributed are defined by its fecundity, dispersal distance, early development mode, role in sediment engineering, position in the sediment, contribution to the infaunal spawner pool and the relative abundance of sediment stabilizing and destabilizing groups

[ ask patches with [sp = 35] [ set sp one-of (se n-values ((fg1 / fg) * 200) [45] n-values ((fg2 / fg) * 50) [55] n-values ((fg3 / fg) * 100) [65] n-values ((fg4 / fg) * 150) [75] n-values ((fg5 / fg) * 600) [85] n-values ((fg6 / fg) * 100) [95] n-values ((fg7 / fg) * 150) [105] n-values ((fg8 / fg) * 150) [115] n-values ((fg9 / fg) * 600) [125] n-values ((fg10 / fg) * 600) [135] n-values ((fg11 / fg) * 150) [145]) ] ]

[ ask patches with [sp = 35] [ set sp one-of (se n-values ((fg1 / fg) * 200) [45] n-values ((fg2 / fg) * 50) [55] n-values ((fg3 / fg) * 100) [65] n-values ((fg4 / fg) * 600) [75] n-values ((fg5 / fg) * 150) [85] n-values ((fg6 / fg) * 100) [95] n-values ((fg7 / fg) * 600) [105] n-values ((fg8 / fg) * 150) [115] n-values ((fg9 / fg) * 150) [125] n-values ((fg10 / fg) * 150) [135] n-values ((fg11 / fg) * 600) [145]) ] ]

ask n-of ( 0.9 * count patches with [ sp = 45 or sp = 55 or sp = 65 or sp = 75 ] ) patches with [ sp = 45 or sp = 55 or sp = 65 or sp = 75 ] [ set sp 35 ] ; randomly remove a subset of newly settled infaunal juveniles of each group from the system/juvenile mortality rates are defined by the body size of each group

ask n-of ( 0.5 * count patches with [ sp = 85 or sp = 95 or sp = 105 or sp = 115 or sp = 125 ] ) patches with [ sp = 85 or sp = 95 or sp = 105 or sp = 115 or sp = 125 ] [ set sp 35 ]

ask n-of ( 0.1 * count patches with [ sp = 135 or sp = 145 ] ) patches with [ sp = 135 or sp = 145 ] [ set sp 35 ]

let fge ( fg9 + fg12 + fg13 + fg14 + fg15 + fg16 ) ; define the contribution of epifaunal groups to the respective spawner pool as the contribution of each group to the total abundance of the respective set of groups during the previous time step

ask patches with [sp = 9 and ep = 0] [ set ep one-of (se n-values ((fg9 / fge) * 300) [195] n-values ((fg12 / fge) * 300) [165] n-values ((fg13 / fge) * 300) [175] n-values ((fg14 / fge) * 300) [185] n-values ((fg15 / fge) * 300) [205] n-values ((fg16 / fg) * 200) [215] ) ] ; randomly attribute an epifaunal group to the epifaunal group occupation variable of patches occupied by infaunal individuals of the basibiotic group that are not occupied by epifauna/the chances of each group to be attributed are defined by its fecundity, dispersal distance, early development mode and contribution to the infaunal spawner pool

ask n-of ( 0.9 * count patches with [ ep = 165 or ep = 175 or ep = 185 ] ) patches with [ ep = 165 or ep = 175 or ep = 185 ] [ set ep 0 ] ; randomly remove a subset of newly settled epifaunal juveniles of each group from the system/juvenile mortality rates are defined by the body size of each group

ask n-of ( 0.5 * count patches with [ ep = 195 ] ) patches with [ ep = 195 ] [ set ep 0 ]

ask n-of ( 0.1 * count patches with [ ep = 205 or ep = 215 ] ) patches with [ ep = 205 or ep = 215 ] [ set ep 0 ]

end

to do-competition ; run the competition submodel

ask patches with [sp > 80 and sp < 120] [ ; in random order expand patch occupation of surviving juveniles of purely infaunal intermediate groups to 8 immediate neighbors, unless any of them is occupied by an infaunal individual of the same size

if map [(? < 5 or ? > 11) and (? < 85 or ? > 125)] [sp] of neighbors = [true true true true true true true true] [

ask neighbors [set sp [sp] of myself]

set g 1 / ticks ; assign the value 1/time step to the central patches variable of purely infaunal intermediate individuals that grew in spite of competition

]

]

ask patches with [sp = 125] [ ; in random order expand patch occupation of surviving juveniles of the basibiotic group to 8 immediate neighbors, unless any of them is occupied by an infaunal individual of the same size and provided that at least one patch within each patch's 24 closest neighbors is occupied by an individual of the same group

if map [(? < 5 or ? > 11) and (? < 85 or ? > 125)] [sp] of neighbors = [true true true true true true true true] and count patches in-radius 2.9 with [sp = 9] > 0 [

ask neighbors [set sp [sp] of myself]

set g 1 / ticks ; assign the value 1/time step to the central patches variable of basibiotic intermediate individuals that grew in spite of competition

]

]

ask patches with [sp > 130] [ ; in random order expand patch occupation of surviving juveniles of infaunal large groups to 24 closest neighbors, unless any of them is occupied by an infaunal individual of the same size

if map [(? < 10 or ? > 11) and ? < 135] [sp] of other patches in-radius 2.9 = [true true true true true true true true true true true true true true true true true true true true true true true true] [

ask patches in-radius 2.9 [set sp [sp] of myself]

set g 2 * ticks ; assign the value 2xtime step to the central patches variable of large infaunal individuals that grew in spite of competition

]

]

ask patches with [ep = 215] [ ; in random order expand patch occupation of surviving juveniles of epifaunal intermediate groups associated with rock to 8 immediate neighbors, unless any of them is occupied by an epifaunal individual of the same size

if map [(? < 17 or ? > 18) and ? < 205] [ep] of neighbors = [true true true true true true true true] [

ask neighbors [set ep [ep] of myself]

set h 2 * ticks ; assign the value 2xtime step to the central patches variable of epifaunal intermediate individuals associated with rock that grew in spite of competition

]

]

ask patches with [ep = 205] [ ; in random order expand patch occupation of surviving juveniles of epifaunal intermediate groups associated with gravel to 8 immediate neighbors, unless any of them is occupied by an epifaunal individual of the same size

if map [(? < 17 or ? > 18) and ? < 205] [ep] of neighbors = [true true true true true true true true] [

ask neighbors [set ep [ep] of myself]

set h 2 * ticks ; assign the value 2xtime step to the central patches variable of epifaunal intermediate individuals associated with gravel that grew in spite of competition

]

]

end

to do-ageing ; run the ageing submodel

ask s [set a a + 1] ; add one year to the age of small infaunal individuals that survived from the previous time step

ask m [set a a + 1] ; add one year to the age of intermediate infaunal individuals that survived from the previous time step

ask l [set a a + 1] ; add one year to the age of large infaunal individuals that survived from the previous time step

ask sm [set ag ag + 1] ; add one year to the age of small epifaunal individuals that survived from the previous time step

ask la [set ag ag + 1] ; add one year to the age of intermediate epifaunal individuals that survived from the previous time step

end

to do-mortality ; run the mortality submodel

ask patches with [sp > 40 and sp < 80] [set sp (sp - 35) / 10 set a 0] ; set the age of newly settled small infaunal individuals that were not overgrown to 1 year and

set s patches with [sp < 5] ; add them to the respective patch set

ask m with [length (filter [? = sp] [sp] of patches in-radius 1.9) != 9 or sp > 9] [ask patches in-radius 1.9 with [sp < 130] [set sp 35] ask la in-radius 1.9 [set ep 0] ask patches in-radius 1.9 with [ep < 17 or (ep > 160 and ep < 200) or h = 2 * ticks] [set ep 0]] ; clear the infaunal and epifaunal group occupation variable of patches occupied by intermediate infaunal individuals that were overgrown

set m m with [sp < 10] ; remove the central patches of intermediate infaunal individuals that were overgrown from the respective patch set of individuals that survive at the end of the year

ask patches with [g = 1 / ticks and sp > 80 and sp < 130 and length (filter [? = sp] [sp] of patches in-radius 1.9) = 9] [ask patches in-radius 1.9 [set sp (sp - 35) / 10] set g -1 / ticks set a 0] ; assign the value -1/time step to the central patches variable of intermediate infaunal individuals that grew and were not overgrown and set their age to 1 year

set m (patch-set m patches with [g = -1 / ticks]) ; add the central patches of intermediate infaunal individuals that grew and were not overgrown to the respective patch set

ask patches with [g = 2 * ticks] [ask patches in-radius 2.9 [set sp (sp - 35) / 10] set a 0] ; set the age of newly settled large infaunal individuals that grew to 1 year and

set l (patch-set l patches with [g = 2 * ticks]) ; add them to the respective patch set

ask patches with [sp > 11] [set sp 35] ; clear the infaunal group occupation variable of patches occupied by newly settled infaunal individuals that did not grow or were overgrown

ask s with [sp = 1 and a > 0] [set sp 35] ; clear the infaunal group occupation variable of patches occupied by small infaunal individuals that reached their lifespan

ask s with [sp = 2 and a > 8] [set sp 35]

ask s with [sp = 3 and a > 0] [set sp 35]

ask s with [sp = 4 and a > 0] [set sp 35]

ask m with [sp = 5 and a > 2] [ask patches in-radius 1.9 [set sp 35]] ; clear the infaunal group occupation variable of patches occupied by intermediate infaunal individuals that reached their lifespan

ask m with [sp = 6 and a > 13] [ask patches in-radius 1.9 [set sp 35]]

ask m with [sp = 7 and a > 1] [ask patches in-radius 1.9 [set sp 35]]

ask m with [sp = 8 and a > 7] [ask patches in-radius 1.9 [set sp 35]]

ask patches with [ep > 160 and ep < 200] [set ep (ep - 35) / 10 set ag 0] ; set the age of newly settled small epifaunal individuals that were not overgrown to 1 year

ask sm with [ep = 13 and ag > 0] [set ep 0] ; clear the epifaunal group occupation variable of patches occupied by small epifaunal individuals that reached their lifespan

ask sm with [ep = 14 and ag > 0] [set ep 0]

ask sm with [ep = 15 and ag > 0] [set ep 0]

ask sm with [ep = 16 and ag > 9] [set ep 0]

ask m with [sp = 9 and a > 9] [ ; ask infaunal individuals of the basibiotic group that reached their lifespan

ifelse length (filter [? = 16] [ep] of patches in-radius 1.9) > 0 ; if they have any epibiotic individuals of the same group

[set a max [ag] of patches in-radius 1.9 with [ep = 16] ask max-one-of patches in-radius 1.9 with [ep = 16] [ag] [set ep 0]] ; to take the age of the oldest one and remove it from the epibionts

[ask patches in-radius 1.9 [set sp 35 set ep 0]]] ; otherwise clear the infaunal and epifaunal group occupation variable of patches occupied by them

set sm patches with [ep > 0 and ep < 17] ; add newly settled small epifaunal individuals that were not overgrown to the respective patch set

ask la with [length (filter [? = ep] [ep] of patches in-radius 1.9) != 9] [ask patches in-radius 1.9 [set ep 0]] ; clear the epifaunal group occupation variable of patches occupied by intermediate epifaunal individuals that are no more epibionts

ask patches with [h = 2 * ticks and ep > 200] [ask patches in-radius 1.9 [set ep (ep - 35) / 10] set h -2 * ticks set ag 0] ; set the age of newly settled intermediate epifaunal individuals that grew to 1 year and

set la (patch-set la with [ep > 0] patches with [h = -2 * ticks]) ; add them to the respective patch set

ask patches with [ep > 18] [set ep 0] ; clear the epifaunal group occupation variable of patches occupied by intermediate epifaunal individuals that did not grow

ask la with [ep = 17 and ag > 8] [ask patches in-radius 1.9 [set ep 0]] ; clear the epifaunal group occupation variable of patches occupied by intermediate epifaunal individuals that reached their lifespan

ask la with [ep = 18 and ag > 10] [ask patches in-radius 1.9 [set ep 0]]

set sm sm with [(ep = 13 and ag < 1) or (ep = 14 and ag < 1) or (ep = 15 and ag < 1) or (ep = 16 and ag < 10)] ; update the patch set of small epifaunal individuals

set la la with [(ep = 17 and ag < 9) or (ep = 18 and ag < 11)] ; update the patch set of intermediate epifaunal individuals

ask l with [sp = 10 and a > 2] [ask patches in-radius 2.9 [set sp 35]] ; clear the infaunal group occupation variable of patches occupied by large infaunal individuals that reached their lifespan

ask l with [sp = 11 and a > 1] [ask patches in-radius 2.9 [set sp 35]]

let pr4 count s with [sp = 4] ; assign the abundances of prey groups to the potential prey variables

let pr5 count m with [sp = 5]

let pr7 count m with [sp = 7]

let pr11 count l with [sp = 11]

ifelse pr11 >= pr7 ; if larger prey was more or equally abundant to smaller prey

[ask n-of min list p2 count l with [sp = 11 and a < 2] l with [sp = 11 and a < 2] [ask patches in-radius 2.9 [set sp 35]]] ; randomly remove as many larger prey individuals as there were large predators

[ask n-of min list max list 0 (p2 - count m with [sp = 7 and a < 2]) count l with [sp = 11 and a < 2] l with [sp = 11 and a < 2] [ask patches in-radius 2.9 [set sp 35]]] ; otherwise randomly remove as many larger prey individuals as there were large predators minus the abundance of the smaller prey

set l l with [(sp = 10 and a < 3) or (sp = 11 and a < 2)] ; update the patch set of large epifaunal individuals

ifelse pr7 > pr11 ; if smaller prey was more abundant than larger prey

[ask n-of min list p2 count m with [sp = 7 and a < 2] m with [sp = 7 and a < 2] [ask patches in-radius 1.9 [set sp 35]]] ; randomly remove as many smaller prey individuals as there were large predators

[ask n-of min list max list 0 (p2 - count l with [sp = 11 and a < 2]) count m with [sp = 7 and a < 2] m with [sp = 7 and a < 2] [ask patches in-radius 1.9 [set sp 35]]] ; otherwise randomly remove as many smaller prey individuals as there were large predators minus the abundance of the larger prey

ifelse pr5 >= pr4 ; if larger prey was more or equally abundant to smaller prey

[ask n-of min list p3 count m with [sp = 5 and a < 3] m with [sp = 5 and a < 3] [ask patches in-radius 1.9 [set sp 35]]] ; randomly remove as many larger prey individuals as there were intermediate predators

[ask n-of min list max list 0 (p3 - count s with [sp = 4 and a < 1]) count m with [sp = 5 and a < 3] m with [sp = 5 and a < 3] [ask patches in-radius 1.9 [set sp 35]]] ; otherwise randomly remove as many larger prey individuals as there were intermediate predators minus the abundance of the smaller prey

ifelse pr7 >= pr4 ; if larger prey was more or equally abundant to smaller prey

[ask n-of min list p1 count m with [sp = 7 and a < 2] m with [sp = 7 and a < 2] [ask patches in-radius 1.9 [set sp 35]]] ; randomly remove as many larger prey individuals as there were small predators

[ask n-of min list max list 0 (p1 - count s with [sp = 4 and a < 1]) count m with [sp = 7 and a < 2] m with [sp = 7 and a < 2] [ask patches in-radius 1.9 [set sp 35]]] ; otherwise randomly remove as many larger prey individuals as there were small predators minus the abundance of the smaller prey

set m m with [(sp = 5 and a < 3) or (sp = 6 and a < 14) or (sp = 7 and a < 2) or (sp = 8 and a < 8) or (sp = 9 and a < 10)] ; update the patch set of intermediate epifaunal individuals

ifelse pr4 > pr5 ; if smaller prey was more abundant than larger prey

[ask n-of min list p3 count s with [sp = 4] s with [sp = 4] [set sp 35]] ; randomly remove as many smaller prey individuals as there were intermediate predators

[ask n-of min list max list 0 (p3 - count m with [sp = 5]) count s with [sp = 4] s with [sp = 4] [set sp 35]] ; otherwise randomly remove as many smaller prey individuals as there were small predators minus the abundance of the larger prey

ifelse pr4 > pr7 ; if smaller prey was more abundant than larger prey

[ask n-of min list p1 count s with [sp = 4] s with [sp = 4] [set sp 35]] ; randomly remove as many smaller prey individuals as there were small predators

[ask n-of min list max list 0 (p1 - count m with [sp = 7]) count s with [sp = 4] s with [sp = 4] [set sp 35]] ; otherwise randomly remove as many smaller prey individuals as there were small predators minus the abundance of the larger prey

set s s with [(sp = 1 and a < 1) or (sp = 2 and a < 9) or (sp = 3 and a < 1) or (sp = 4 and a < 1)] ; update the patch set of small epifaunal individuals

set g1 count patches with [ sp = 1 ] ; give the small infaunal groups abundance variables values equal to the number of patches occupied by each group

set g2 count patches with [ sp = 2 ]

set g3 count patches with [ sp = 3 ]

set g4 count patches with [ sp = 4 ]

set g5 count patches with [ sp = 5 ] / 9 ; give the intermediate infaunal groups abundance variables values equal to the number of patches occupied by each group divided by the individually occupied number of patches

set g6 count patches with [ sp = 6 ] / 9

set g7 count patches with [ sp = 7 ] / 9

set g8 count patches with [ sp = 8 ] / 9

set g9 count patches with [ sp = 9 ] / 9

set ba g9 ; give the infaunal basibionts abundance variable a value equal to the infaunal abundance of the basibiotic group

set g10 count patches with [ sp = 10 ] / 25 ; give the large infaunal groups abundance variables values equal to the number of patches occupied by each group divided by the individually occupied number of patches

set g11 count patches with [ sp = 11 ] / 25

set p1 round ((g4 + g7) / 10) ; give the predatory groups abundance variables values equal to the sum of their prey groups abundance divided by 10

set p3 round ((g4 + g5) / 10)

set p2 round ((g7 + g11) / 10)

set p4 round ((p1 + p3) / 10)

set es (g5 + g10) ; give the sediment stabilizing groups abundance variable a value equal to the sum of the abundances of intermediate and large sessile stabilizers

set ed (g7 + g11) ; give the sediment destabilizing groups abundance variable a value equal to the sum of the abundances of intermediate and large destabilizers

ask patches [set pcolor sp] ; color patches according to their infaunal group occupation variable

set g12 count patches with [ ep = 13 ] ; give the small epifaunal groups abundance variables values equal to the number of patches occupied by each group

set g13 count patches with [ ep = 14 ]

set g14 count patches with [ ep = 15 ]

set g9 g9 + count patches with [ ep = 16 ] ; give the basibiotic group abundance variable a value equal to the sum of the abundances of the infaunal and epifaunal individuals of the group

set g15 count patches with [ ep = 17 ] / 9 ; give the intermediate epifaunal groups abundance variables values equal to the number of patches occupied by each group divided by the individually occupied number of patches

set g16 count patches with [ ep = 18 ] / 9

ask patches with [ ep > 0 ] [ set pcolor ep ] ; color patches occupied by epifaunal individuals according to their epifaunal group occupation variable

output-type "g " output-type g1 output-type " " output-type g2 output-type " " output-type g3 output-type " " output-type g4 output-type " " output-type g5 output-type " " output-type g6 output-type " " output-type g7 output-type " " output-type g8 output-type " " output-type g9 output-type " " output-type g10 output-type " " output-type g11 output-type " " output-type g12 output-type " " output-type g13 output-type " " output-type g14 output-type " " output-type g15 output-type " " output-type g16 output-type " " output-type p1 output-type " " output-type p2 output-type " " output-type p3 output-type " " output-print p4 ; print out the values of infaunal/epifaunal and predatory groups abundance

set n length filter [? > 0] (list g1 g2 g3 g4 g5 g6 g7 g8 g9 g10 g11 g12 g13 g14 g15 g16 p1 p2 p3 p4) ; give the variable of number of functional groups in the system a value equal to the number of groups with abundances larger than 0

end

GRAPHICS-WINDOW

501

10

995

525

60

60

4.0

1

4

1

1

1

0

1

1

1

-60

60

-60

60

1

1

1

ticks

30.0

BUTTON

1062

10

1135

43

NIL

setup

NIL

1

T

OBSERVER

NIL

NIL

NIL

NIL

1

PLOT

37

274

484

537

Evolution of functional group abundance

Ticks

Abundance

0.0

0.0

0.0

0.0

true

true

"" ""

PENS

"FG1" 1.0 0 -2674135 true "" "plot g1"

"FG2" 1.0 0 -955883 true "" "plot g2"

"FG3" 1.0 0 -1184463 true "" "plot g3"

"FG4" 1.0 0 -10899396 true "" "plot g4"

"FG5" 1.0 0 -13840069 true "" "plot g5"

"FG6" 1.0 0 -11221820 true "" "plot g6"

"FG7" 1.0 0 -13791810 true "" "plot g7"

"FG8" 1.0 0 -8630108 true "" "plot g8"

"FG9" 1.0 0 -6459832 true "" "plot g9"

"FG10" 1.0 0 -5825686 true "" "plot g10"

"FG11" 1.0 0 -2064490 true "" "plot g11"

MONITOR

1033

452

1156

501

Number of groups

n

0

1

12

PLOT

37

10

484

273

Functional group abundance

Functional group

Abundance

0.0

20.0

0.0

10.0

true

false

"" ""

PENS

"5" 1.0 1 -16777216 true "" "plotxy 4 g5"

"6" 1.0 1 -16777216 true "" "plotxy 5 g6"

"7" 1.0 1 -16777216 true "" "plotxy 6 g7"

"8" 1.0 1 -16777216 true "" "plotxy 7 g8"

"9" 1.0 1 -16777216 true "" "plotxy 8 g9"

"10" 1.0 1 -16777216 true "" "plotxy 9 g10"

"1" 1.0 1 -16777216 true "" "plotxy 0 g1"

"2" 1.0 1 -16777216 true "" "plotxy 1 g2"

"3" 1.0 1 -16777216 true "" "plotxy 2 g3"

"4" 1.0 1 -16777216 true "" "plotxy 3 g4"

"pen-10" 1.0 1 -16777216 true "" "plotxy 16 p1"

"pen-11" 1.0 1 -16777216 true "" "plotxy 17 p2"

"pen-12" 1.0 1 -16777216 true "" "plotxy 18 p3"

"pen-13" 1.0 1 -16777216 true "" "plotxy 19 p4"

"pen-14" 1.0 1 -16777216 true "" "plotxy 10 g11"

"pen-15" 1.0 1 -16777216 true "" "plotxy 11 g12"

"pen-16" 1.0 1 -16777216 true "" "plotxy 12 g13"

"pen-17" 1.0 1 -16777216 true "" "plotxy 13 g14"

"pen-18" 1.0 1 -16777216 true "" "plotxy 14 g15"

"pen-19" 1.0 1 -16777216 true "" "plotxy 15 g16"

BUTTON

1068

66

1131

99

go

go

T

1

T

OBSERVER

NIL

NIL

NIL

NIL

1

BUTTON

1042

120

1159

153

make-movie

make-movie

NIL

1

T

OBSERVER

NIL

NIL

NIL

NIL

1

MONITOR

1033

281

1189

330

stabilizer abundance

es

0

1

12

MONITOR

1033

338

1189

387

destabilizer abundance

ed

0

1

12

BUTTON

1060

173

1138

206

export

export

NIL

1

T

OBSERVER

NIL

NIL

NIL

NIL

1

BUTTON

1061

226

1139

259

import

import

NIL

1

T

OBSERVER

NIL

NIL

NIL

NIL

1

PLOT

37

538

484

801

Evolution of predatory group abundance

Ticks

Abundance

0.0

0.0

0.0

0.0

true

true

"" ""

PENS

"FGP1" 1.0 0 -2674135 true "" "plot p1"

"FGP2" 1.0 0 -1184463 true "" "plot p2"

"FGP3" 1.0 0 -13840069 true "" "plot p3"

"FGP4" 1.0 0 -13345367 true "" "plot p4"

OUTPUT

501

550

1189

801

12

MONITOR

1033

395

1189

444

basibiont abundance

ba

0

1

12

**S.4.2 Intertidal version of the fine-scale model**

globals [es ed s m l sm la xy n p1 p2 p3 p4 g1 g2 g3 g4 g5 g6 g7 g8 g9 g10 g11 g12 g13 g14 g15 g16 ba fg1 fg2 fg3 fg4 fg5 fg6 fg7 fg8 fg9 fg10 fg11 fg12 fg13 fg14 fg15 fg16] ; define variables of abundances of sediment stabilizing (es) and destabilizing (ed) groups, central patches of the individuals of small (s), intermediate (m), large (l) infaunal and small (sm), intermediate (la) epifaunal groups that survive at the end of each year, IBM name according to x-y coordinates of respective Rance patch (xy), number of functional groups in the system (n), abundances of 4 predatory groups (p1-p4), abundances of 16 infaunal/epifaunal groups (g1-g16), abundance of infaunal individuals of basibiotic group (ba) and contributions of infaunal/epifaunal groups to the respective spawner pool (fg1-fg16)

patches-own [sp ep g h a ag] ; define patch variables of infaunal (sp) and epifaunal (ep) group occupation, central patches of intermediate and large infaunal (g) and intermediate epifaunal (h) individuals that grow in spite of competition and age of infaunal (a) and epifaunal (ag) individuals that survive at the end of a year

to make-movie ; make movie of model interface during initialization plus 9 time steps

user-message "First, save your new movie file (choose a name ending with .mov)"

let path user-new-file

if not is-string? path [ stop ]

setup

movie-start path

movie-set-frame-rate 1

movie-grab-interface

while [ ticks < 10 ]

[go

movie-grab-interface ]

movie-close

user-message (word "Exported movie to " path)

end

to export ; export all model entities and output to an external file named after the value of the xy variable

export-world (word xy ".csv")

end

to import ; import all model entities and output from an external file named after the value of the xy variable

import-world (word xy ".csv")

end

to setup ; initialize the model

clear-all ; clear all model entities and output

set s no-patches ; set variable s to an empty patch agentset

set m no-patches ; set variable m to an empty patch agentset

set l no-patches ; set variable l to an empty patch agentset

set sm no-patches ; set variable sm to an empty patch agentset

set la no-patches ; set variable la to an empty patch agentset

ask patches [set sp one-of (list 45 45 55 65 65 65 65 75 75 75 85 85 85 95 95 105 105 105 105 105 105 115 115 115 115 115 115 125 125 125 135 135 135 135 135 135 145 145 145 145 145 145)] ; randomly attribute an infaunal group to the infaunal group occupation variable of each patch/the chances of each group to be attributed are defined by its tolerance of tidal exposure, fecundity, dispersal distance and early development mode

ask n-of ( 0.9 * count patches with [ sp = 45 or sp = 55 or sp = 65 or sp = 75 ] ) patches with [ sp = 45 or sp = 55 or sp = 65 or sp = 75 ] [ set sp 35 ] ; randomly remove a subset of newly settled infaunal juveniles of each group from the system/juvenile mortality rates are defined by the body size of each group

ask n-of ( 0.5 * count patches with [ sp = 85 or sp = 95 or sp = 105 or sp = 115 or sp = 125] ) patches with [ sp = 85 or sp = 95 or sp = 105 or sp = 115 or sp = 125] [ set sp 35 ]

ask n-of ( 0.1 * count patches with [ sp = 135 or sp = 145 ] ) patches with [ sp = 135 or sp = 145 ] [ set sp 35 ]

ask patches with [sp > 80 and sp < 120] [ ; in random order expand patch occupation of surviving juveniles of purely infaunal intermediate groups to 8 immediate neighbors, unless any of them is occupied by an infaunal individual of the same size

if map [? < 85 or ? > 125] [sp] of neighbors = [true true true true true true true true] [

ask neighbors [set sp [sp] of myself]

set g -3 ; assign the value -3 to the central patches variable of purely infaunal intermediate individuals that grew in spite of competition

]

]

ask patches with [sp = 125] [ ; in random order expand patch occupation of surviving juveniles of the basibiotic group to 8 immediate neighbors, unless any of them is occupied by an infaunal individual of the same size and provided that at least one patch within each patch's 24 closest neighbors is occupied by an individual of the same group

if map [? < 85 or ? > 125] [sp] of neighbors = [true true true true true true true true] and count patches in-radius 2.9 with [sp = 125] > 1 [

ask neighbors [set sp [sp] of myself]

set g -3 ; assign the value -3 to the central patches variable of basibiotic intermediate individuals that grew in spite of competition

]

]

ask patches with [sp > 130] [ ; in random order expand patch occupation of surviving juveniles of infaunal large groups to 24 closest neighbors, unless any of them is occupied by an infaunal individual of the same size

if map [? < 135] [sp] of other patches in-radius 2.9 = [true true true true true true true true true true true true true true true true true true true true true true true true] [

ask patches in-radius 2.9 [set sp [sp] of myself]

set g -5 ; assign the value -5 to the central patches variable of large infaunal individuals that grew in spite of competition

]

]

ask patches with [sp > 40 and sp < 80] [set sp (sp - 35) / 10 set g -11 set a 0] ; assign the value -11 to the central patches variable of small infaunal individuals that were not overgrown and set their age to 1 year

set s (patch-set patches with [g = -11]) ; assign the central patches of small infaunal individuals that were not overgrown to the respective patch set

ask patches with [g = -3 and sp < 130 and length (filter [? = sp] [sp] of patches in-radius 1.9) = 9] [ask patches in-radius 1.9 [set sp (sp - 35) / 10] set g -7 set a 0] ; assign the value -7 to the central patches variable of intermediate infaunal individuals that grew and were not overgrown and set their age to 1 year

set m (patch-set patches with [g = -7]) ; assign the central patches of intermediate infaunal individuals that grew and were not overgrown to the respective patch set

ask patches with [g = -5 and length (filter [? = sp] [sp] of patches in-radius 2.9) = 25] [ask patches in-radius 2.9 [set sp (sp - 35) / 10] set g -9 set a 0] ; assign the value -9 to the central patches variable of large infaunal individuals that grew and were not overgrown and set their age to 1 year

set l (patch-set patches with [g = -9]) ; assign the central patches of large infaunal individuals that grew and were not overgrown to the respective patch set

ask patches with [sp > 11] [set sp 35] ; clear the infaunal group occupation variable of patches occupied by infaunal individuals that did not grow or were overgrown

ask patches with [sp = 9] [set ep one-of (list 165 165 165 175 175 175 175 175 175 185 185 185 195 195 195 205 205 205 215 215)] ; randomly attribute an epifaunal group to the epifaunal group occupation variable of patches occupied by infaunal individuals of the basibiotic group/the chances of each group to be attributed are defined by its tolerance of tidal exposure, fecundity, dispersal distance and early development mode

ask n-of ( 0.9 * count patches with [ ep = 165 or ep = 175 or ep = 185 ] ) patches with [ ep = 165 or ep = 175 or ep = 185 ] [ set ep 0 ] ; randomly remove a subset of newly settled epifaunal juveniles of each group from the system/juvenile mortality rates are defined by the body size of each group

ask n-of ( 0.5 * count patches with [ ep = 195 ] ) patches with [ ep = 195 ] [ set ep 0 ]

ask n-of ( 0.1 * count patches with [ ep = 205 or ep = 215 ] ) patches with [ ep = 205 or ep = 215 ] [ set ep 0 ]

ask patches with [ep = 215] [ ; in random order expand patch occupation of surviving juveniles of epifaunal intermediate groups associated with rock to 8 immediate neighbors, unless any of them is occupied by an epifaunal individual of the same size

if map [? < 200] [ep] of neighbors = [true true true true true true true true] [

ask neighbors [set ep [ep] of myself]

set h 3 ; assign the value 3 to the central patches variable of epifaunal intermediate individuals associated with rock that grew in spite of competition

]

]

ask patches with [ep = 205] [ ; in random order expand patch occupation of surviving juveniles of epifaunal intermediate groups associated with gravel to 8 immediate neighbors, unless any of them is occupied by an epifaunal individual of the same size

if map [? < 200] [ep] of neighbors = [true true true true true true true true] [

ask neighbors [set ep [ep] of myself]

set h 3 ; assign the value 3 to the central patches variable of epifaunal intermediate individuals associated with gravel that grew in spite of competition

]

]

ask patches with [ep > 160 and ep < 200] [set ep (ep - 35) / 10 set h 5 set ag 0] ; assign the value 5 to the central patches variable of small epifaunal individuals that were not overgrown and set their age to 1 year

set sm (patch-set patches with [h = 5]) ; assign the central patches of small epifaunal individuals that were not overgrown to the respective patch set

ask patches with [h = 3 and length (filter [? = ep] [ep] of patches in-radius 1.9) = 9] [ask patches in-radius 1.9 [set ep (ep - 35) / 10] set h 7 set ag 0] ; assign the value 7 to the central patches variable of intermediate epifaunal individuals that grew and were not overgrown and set their age to 1 year

set la (patch-set patches with [h = 7]) ; assign the central patches of intermediate epifaunal individuals that grew and were not overgrown to the respective patch set

ask patches with [ep > 18] [set ep 0] ; clear the epifaunal group occupation variable of patches occupied by epifaunal individuals that did not grow

set g1 count patches with [ sp = 1 ] ; give the small infaunal groups abundance variables values equal to the number of patches occupied by each group

set g2 count patches with [ sp = 2 ]

set g3 count patches with [ sp = 3 ]

set g4 count patches with [ sp = 4 ]

set g5 count patches with [ sp = 5 ] / 9 ; give the intermediate infaunal groups abundance variables values equal to the number of patches occupied by each group divided by the individually occupied number of patches

set g6 count patches with [ sp = 6 ] / 9

set g7 count patches with [ sp = 7 ] / 9

set g8 count patches with [ sp = 8 ] / 9

set g9 count patches with [ sp = 9 ] / 9

set ba g9 ; give the infaunal basibionts abundance variable a value equal to the infaunal abundance of the basibiotic group

set g10 count patches with [ sp = 10 ] / 25 ; give the large infaunal groups abundance variables values equal to the number of patches occupied by each group divided by the individually occupied number of patches

set g11 count patches with [ sp = 11 ] / 25

set p1 round ((max list g4 g7) / 10) ; give the predatory groups abundance variables values equal to those of the most abundant of their prey groups divided by 10

set p3 round ((max list g4 g5) / 10)

set p2 round ((max list g7 g11) / 10)

set p4 round ((max list p1 p3) / 10)

set es (g8 + g10) ; give the sediment stabilizing groups abundance variable a value equal to the sum of the abundances of intermediate and large sessile stabilizers that tolerate tidal exposure

set ed (g7 + g11) ; give the sediment destabilizing groups abundance variable a value equal to the sum of the abundances of intermediate and large destabilizers that tolerate tidal exposure

ask patches [set pcolor sp] ; color patches according to their infaunal group occupation variable

set g12 count patches with [ ep = 13 ] ; give the small epifaunal groups abundance variables values equal to the number of patches occupied by each group

set g13 count patches with [ ep = 14 ]

set g14 count patches with [ ep = 15 ]

set g9 g9 + count patches with [ ep = 16 ] ; give the basibiotic group abundance variable a value equal to the sum of the abundances of the infaunal and epifaunal individuals of the group

set g15 count patches with [ ep = 17 ] / 9 ; give the intermediate epifaunal groups abundance variables values equal to the number of patches occupied by each group divided by the individually occupied number of patches

set g16 count patches with [ ep = 18 ] / 9

ask patches with [ ep > 0 ] [ set pcolor ep ] ; color patches occupied by epifaunal individuals according to their epifaunal group occupation variable

set n length filter [? > 0] (list g1 g2 g3 g4 g5 g6 g7 g8 g9 g10 g11 g12 g13 g14 g15 g16 p1 p2 p3 p4) ; give the variable of number of functional groups in the system a value equal to the number of groups with abundances larger than 0

reset-ticks ; set tick counter to zero, set up and update all plots

end

to go ; move the model one step forward

set-current-plot "Functional group abundance" ; clear the histogram of functional group abundances

clear-plot

tick ; advance the tick counter by one and update all plots

do-recruitment ; run the recruitment submodel

do-competition ; run the competition submodel

do-ageing ; run the ageing submodel

do-mortality ; run the mortality submodel

end

to do-recruitment ; run the recruitment submodel

output-print " " output-type "f " output-type fg1 output-type " " output-type fg2 output-type " " output-type fg3 output-type " " output-type fg4 output-type " " output-type fg5 output-type " " output-type fg6 output-type " " output-type fg7 output-type " " output-type fg8 output-type " " output-type fg9 output-type " " output-type fg10 output-type " " output-type fg11 output-type " " output-type fg12 output-type " " output-type fg13 output-type " " output-type fg14 output-type " " output-type fg15 output-type " " output-print fg16 ; print out the values of infaunal/epifaunal groups contribution to the respective spawner pool

let fg ( fg1 + fg2 + fg3 + fg4 + fg5 + fg6 + fg7 + fg8 + fg9 + fg10 + fg11 ) ; define the contribution of infaunal groups to the respective spawner pool as the contribution of each group to the total abundance of the respective set of groups during the previous time step

ifelse es > ed ; randomly attribute an infaunal group to the infaunal group occupation variable of patches that are not occupied by infauna/the chances of each group to be attributed are defined by its tolerance of tidal exposure, fecundity, dispersal distance, early development mode, role in sediment engineering, position in the sediment, contribution to the infaunal spawner pool and the relative abundance of sediment stabilizing and destabilizing groups

[ ask patches with [sp = 35] [ set sp one-of (se n-values ((fg1 / fg) * 100) [45] n-values ((fg2 / fg) * 50) [55] n-values ((fg3 / fg) * 100) [65] n-values ((fg4 / fg) * 150) [75] n-values ((fg5 / fg) * 150) [85] n-values ((fg6 / fg) * 100) [95] n-values ((fg7 / fg) * 150) [105] n-values ((fg8 / fg) * 600) [115] n-values ((fg9 / fg) * 150) [125] n-values ((fg10 / fg) * 600) [135] n-values ((fg11 / fg) * 150) [145]) ] ]

[ ask patches with [sp = 35] [ set sp one-of (se n-values ((fg1 / fg) * 100) [45] n-values ((fg2 / fg) * 50) [55] n-values ((fg3 / fg) * 400) [65] n-values ((fg4 / fg) * 150) [75] n-values ((fg5 / fg) * 150) [85] n-values ((fg6 / fg) * 100) [95] n-values ((fg7 / fg) * 600) [105] n-values ((fg8 / fg) * 150) [115] n-values ((fg9 / fg) * 150) [125] n-values ((fg10 / fg) * 150) [135] n-values ((fg11 / fg) * 600) [145]) ] ]

ask n-of ( 0.9 * count patches with [ sp = 45 or sp = 55 or sp = 65 or sp = 75 ] ) patches with [ sp = 45 or sp = 55 or sp = 65 or sp = 75 ] [ set sp 35 ] ; randomly remove a subset of newly settled infaunal juveniles of each group from the system/juvenile mortality rates are defined by the body size of each group

ask n-of ( 0.5 * count patches with [ sp = 85 or sp = 95 or sp = 105 or sp = 115 or sp = 125 ] ) patches with [ sp = 85 or sp = 95 or sp = 105 or sp = 115 or sp = 125 ] [ set sp 35 ]

ask n-of ( 0.1 * count patches with [ sp = 135 or sp = 145 ] ) patches with [ sp = 135 or sp = 145 ] [ set sp 35 ]

let fge ( fg9 + fg12 + fg13 + fg14 + fg15 + fg16 ) ; define the contribution of epifaunal groups to the respective spawner pool as the contribution of each group to the total abundance of the respective set of groups during the previous time step

ask patches with [sp = 9 and ep = 0] [ set ep one-of (se n-values ((fg9 / fge) * 300) [195] n-values ((fg12 / fge) * 300) [165] n-values ((fg13 / fge) * 600) [175] n-values ((fg14 / fge) * 300) [185] n-values ((fg15 / fge) * 300) [205] n-values ((fg16 / fg) * 200) [215] ) ] ; randomly attribute an epifaunal group to the epifaunal group occupation variable of patches occupied by infaunal individuals of the basibiotic group that are not occupied by epifauna/the chances of each group to be attributed are defined by its tolerance of tidal exposure, fecundity, dispersal distance, early development mode and contribution to the infaunal spawner pool

ask n-of ( 0.9 * count patches with [ ep = 165 or ep = 175 or ep = 185 ] ) patches with [ ep = 165 or ep = 175 or ep = 185 ] [ set ep 0 ] ; randomly remove a subset of newly settled epifaunal juveniles of each group from the system/juvenile mortality rates are defined by the body size of each group

ask n-of ( 0.5 * count patches with [ ep = 195 ] ) patches with [ ep = 195 ] [ set ep 0 ]

ask n-of ( 0.1 * count patches with [ ep = 205 or ep = 215 ] ) patches with [ ep = 205 or ep = 215 ] [ set ep 0 ]

end

to do-competition ; run the competition submodel

ask patches with [sp > 80 and sp < 120] [ ; in random order expand patch occupation of surviving juveniles of purely infaunal intermediate groups to 8 immediate neighbors, unless any of them is occupied by an infaunal individual of the same size

if map [(? < 5 or ? > 11) and (? < 85 or ? > 125)] [sp] of neighbors = [true true true true true true true true] [

ask neighbors [set sp [sp] of myself]

set g 1 / ticks ; assign the value 1/time step to the central patches variable of purely infaunal intermediate individuals that grew in spite of competition

]

]

ask patches with [sp = 125] [ ; in random order expand patch occupation of surviving juveniles of the basibiotic group to 8 immediate neighbors, unless any of them is occupied by an infaunal individual of the same size and provided that at least one patch within each patch's 24 closest neighbors is occupied by an individual of the same group

if map [(? < 5 or ? > 11) and (? < 85 or ? > 125)] [sp] of neighbors = [true true true true true true true true] and count patches in-radius 2.9 with [sp = 9] > 0 [

ask neighbors [set sp [sp] of myself]

set g 1 / ticks ; assign the value 1/time step to the central patches variable of basibiotic intermediate individuals that grew in spite of competition

]

]

ask patches with [sp > 130] [ ; in random order expand patch occupation of surviving juveniles of infaunal large groups to 24 closest neighbors, unless any of them is occupied by an infaunal individual of the same size

if map [(? < 10 or ? > 11) and ? < 135] [sp] of other patches in-radius 2.9 = [true true true true true true true true true true true true true true true true true true true true true true true true] [

ask patches in-radius 2.9 [set sp [sp] of myself]

set g 2 * ticks ; assign the value 2xtime step to the central patches variable of large infaunal individuals that grew in spite of competition

]

]

ask patches with [ep = 215] [ ; in random order expand patch occupation of surviving juveniles of epifaunal intermediate groups associated with rock to 8 immediate neighbors, unless any of them is occupied by an epifaunal individual of the same size

if map [(? < 17 or ? > 18) and ? < 205] [ep] of neighbors = [true true true true true true true true] [

ask neighbors [set ep [ep] of myself]

set h 2 * ticks ; assign the value 2xtime step to the central patches variable of epifaunal intermediate individuals associated with rock that grew in spite of competition

]

]

ask patches with [ep = 205] [ ; in random order expand patch occupation of surviving juveniles of epifaunal intermediate groups associated with gravel to 8 immediate neighbors, unless any of them is occupied by an epifaunal individual of the same size

if map [(? < 17 or ? > 18) and ? < 205] [ep] of neighbors = [true true true true true true true true] [

ask neighbors [set ep [ep] of myself]

set h 2 * ticks ; assign the value 2xtime step to the central patches variable of epifaunal intermediate individuals associated with gravel that grew in spite of competition

]

]

end

to do-ageing ; run the ageing submodel

ask s [set a a + 1] ; add one year to the age of small infaunal individuals that survived from the previous time step

ask m [set a a + 1] ; add one year to the age of intermediate infaunal individuals that survived from the previous time step

ask l [set a a + 1] ; add one year to the age of large infaunal individuals that survived from the previous time step

ask sm [set ag ag + 1] ; add one year to the age of small epifaunal individuals that survived from the previous time step

ask la [set ag ag + 1] ; add one year to the age of intermediate epifaunal individuals that survived from the previous time step

end

to do-mortality ; run the mortality submodel

ask patches with [sp > 40 and sp < 80] [set sp (sp - 35) / 10 set a 0] ; set the age of newly settled small infaunal individuals that were not overgrown to 1 year and

set s patches with [sp < 5] ; add them to the respective patch set

ask m with [length (filter [? = sp] [sp] of patches in-radius 1.9) != 9 or sp > 9] [ask patches in-radius 1.9 with [sp < 130] [set sp 35] ask la in-radius 1.9 [set ep 0] ask patches in-radius 1.9 with [ep < 17 or (ep > 160 and ep < 200) or h = 2 * ticks] [set ep 0]] ; clear the infaunal and epifaunal group occupation variable of patches occupied by intermediate infaunal individuals that were overgrown

set m m with [sp < 10] ; remove the central patches of intermediate infaunal individuals that were overgrown from the respective patch set of individuals that survive at the end of the year

ask patches with [g = 1 / ticks and sp > 80 and sp < 130 and length (filter [? = sp] [sp] of patches in-radius 1.9) = 9] [ask patches in-radius 1.9 [set sp (sp - 35) / 10] set g -1 / ticks set a 0] ; assign the value -1/time step to the central patches variable of intermediate infaunal individuals that grew and were not overgrown and set their age to 1 year

set m (patch-set m patches with [g = -1 / ticks]) ; add the central patches of intermediate infaunal individuals that grew and were not overgrown to the respective patch set

ask patches with [g = 2 * ticks] [ask patches in-radius 2.9 [set sp (sp - 35) / 10] set a 0] ; set the age of newly settled large infaunal individuals that grew to 1 year and

set l (patch-set l patches with [g = 2 * ticks]) ; add them to the respective patch set

ask patches with [sp > 11] [set sp 35] ; clear the infaunal group occupation variable of patches occupied by newly settled infaunal individuals that did not grow or were overgrown

ask s with [sp = 1 and a > 0] [set sp 35] ; clear the infaunal group occupation variable of patches occupied by small infaunal individuals that reached their lifespan

ask s with [sp = 2 and a > 8] [set sp 35]

ask s with [sp = 3 and a > 0] [set sp 35]

ask s with [sp = 4 and a > 0] [set sp 35]

ask m with [sp = 5 and a > 2] [ask patches in-radius 1.9 [set sp 35]] ; clear the infaunal group occupation variable of patches occupied by intermediate infaunal individuals that reached their lifespan

ask m with [sp = 6 and a > 13] [ask patches in-radius 1.9 [set sp 35]]

ask m with [sp = 7 and a > 1] [ask patches in-radius 1.9 [set sp 35]]

ask m with [sp = 8 and a > 7] [ask patches in-radius 1.9 [set sp 35]]

ask patches with [ep > 160 and ep < 200] [set ep (ep - 35) / 10 set ag 0] ; set the age of newly settled small epifaunal individuals that were not overgrown to 1 year

ask sm with [ep = 13 and ag > 0] [set ep 0] ; clear the epifaunal group occupation variable of patches occupied by small epifaunal individuals that reached their lifespan

ask sm with [ep = 14 and ag > 0] [set ep 0]

ask sm with [ep = 15 and ag > 0] [set ep 0]

ask sm with [ep = 16 and ag > 9] [set ep 0]

ask m with [sp = 9 and a > 9] [ ; ask infaunal individuals of the basibiotic group that reached their lifespan

ifelse length (filter [? = 16] [ep] of patches in-radius 1.9) > 0 ; if they have any epibiotic individuals of the same group

[set a max [ag] of patches in-radius 1.9 with [ep = 16] ask max-one-of patches in-radius 1.9 with [ep = 16] [ag] [set ep 0]] ; to take the age of the oldest one and remove it from the epibionts

[ask patches in-radius 1.9 [set sp 35 set ep 0]]] ; otherwise clear the infaunal and epifaunal group occupation variable of patches occupied by them

set sm patches with [ep > 0 and ep < 17] ; add newly settled small epifaunal individuals that were not overgrown to the respective patch set

ask la with [length (filter [? = ep] [ep] of patches in-radius 1.9) != 9] [ask patches in-radius 1.9 [set ep 0]] ; clear the epifaunal group occupation variable of patches occupied by intermediate epifaunal individuals that are no more epibionts

ask patches with [h = 2 * ticks and ep > 200] [ask patches in-radius 1.9 [set ep (ep - 35) / 10] set h -2 * ticks set ag 0] ; set the age of newly settled intermediate epifaunal individuals that grew to 1 year and

set la (patch-set la with [ep > 0] patches with [h = -2 * ticks]) ; add them to the respective patch set

ask patches with [ep > 18] [set ep 0] ; clear the epifaunal group occupation variable of patches occupied by intermediate epifaunal individuals that did not grow

ask la with [ep = 17 and ag > 8] [ask patches in-radius 1.9 [set ep 0]] ; clear the epifaunal group occupation variable of patches occupied by intermediate epifaunal individuals that reached their lifespan

ask la with [ep = 18 and ag > 10] [ask patches in-radius 1.9 [set ep 0]]

set sm sm with [(ep = 13 and ag < 1) or (ep = 14 and ag < 1) or (ep = 15 and ag < 1) or (ep = 16 and ag < 10)] ; update the patch set of small epifaunal individuals

set la la with [(ep = 17 and ag < 9) or (ep = 18 and ag < 11)] ; update the patch set of intermediate epifaunal individuals

ask l with [sp = 10 and a > 2] [ask patches in-radius 2.9 [set sp 35]] ; clear the infaunal group occupation variable of patches occupied by large infaunal individuals that reached their lifespan

ask l with [sp = 11 and a > 1] [ask patches in-radius 2.9 [set sp 35]]

let pr4 count s with [sp = 4] ; assign the abundances of prey groups to the potential prey variables

let pr5 count m with [sp = 5]

let pr7 count m with [sp = 7]

let pr11 count l with [sp = 11]

ifelse pr11 >= pr7 ; if larger prey was more or equally abundant to smaller prey

[ask n-of min list p2 count l with [sp = 11 and a < 2] l with [sp = 11 and a < 2] [ask patches in-radius 2.9 [set sp 35]]] ; randomly remove as many larger prey individuals as there were large predators

[ask n-of min list max list 0 (p2 - count m with [sp = 7 and a < 2]) count l with [sp = 11 and a < 2] l with [sp = 11 and a < 2] [ask patches in-radius 2.9 [set sp 35]]] ; otherwise randomly remove as many larger prey individuals as there were large predators minus the abundance of the smaller prey

set l l with [(sp = 10 and a < 3) or (sp = 11 and a < 2)] ; update the patch set of large epifaunal individuals

ifelse pr7 > pr11 ; if smaller prey was more abundant than larger prey

[ask n-of min list p2 count m with [sp = 7 and a < 2] m with [sp = 7 and a < 2] [ask patches in-radius 1.9 [set sp 35]]] ; randomly remove as many smaller prey individuals as there were large predators

[ask n-of min list max list 0 (p2 - count l with [sp = 11 and a < 2]) count m with [sp = 7 and a < 2] m with [sp = 7 and a < 2] [ask patches in-radius 1.9 [set sp 35]]] ; otherwise randomly remove as many smaller prey individuals as there were large predators minus the abundance of the larger prey

ifelse pr5 >= pr4 ; if larger prey was more or equally abundant to smaller prey

[ask n-of min list p3 count m with [sp = 5 and a < 3] m with [sp = 5 and a < 3] [ask patches in-radius 1.9 [set sp 35]]] ; randomly remove as many larger prey individuals as there were intermediate predators

[ask n-of min list max list 0 (p3 - count s with [sp = 4 and a < 1]) count m with [sp = 5 and a < 3] m with [sp = 5 and a < 3] [ask patches in-radius 1.9 [set sp 35]]] ; otherwise randomly remove as many larger prey individuals as there were intermediate predators minus the abundance of the smaller prey

ifelse pr7 >= pr4 ; if larger prey was more or equally abundant to smaller prey

[ask n-of min list p1 count m with [sp = 7 and a < 2] m with [sp = 7 and a < 2] [ask patches in-radius 1.9 [set sp 35]]] ; randomly remove as many larger prey individuals as there were small predators

[ask n-of min list max list 0 (p1 - count s with [sp = 4 and a < 1]) count m with [sp = 7 and a < 2] m with [sp = 7 and a < 2] [ask patches in-radius 1.9 [set sp 35]]] ; otherwise randomly remove as many larger prey individuals as there were small predators minus the abundance of the smaller prey

set m m with [(sp = 5 and a < 3) or (sp = 6 and a < 14) or (sp = 7 and a < 2) or (sp = 8 and a < 8) or (sp = 9 and a < 10)] ; update the patch set of intermediate epifaunal individuals

ifelse pr4 > pr5 ; if smaller prey was more abundant than larger prey

[ask n-of min list p3 count s with [sp = 4] s with [sp = 4] [set sp 35]] ; randomly remove as many smaller prey individuals as there were intermediate predators

[ask n-of min list max list 0 (p3 - count m with [sp = 5]) count s with [sp = 4] s with [sp = 4] [set sp 35]] ; otherwise randomly remove as many smaller prey individuals as there were small predators minus the abundance of the larger prey

ifelse pr4 > pr7 ; if smaller prey was more abundant than larger prey

[ask n-of min list p1 count s with [sp = 4] s with [sp = 4] [set sp 35]] ; randomly remove as many smaller prey individuals as there were small predators

[ask n-of min list max list 0 (p1 - count m with [sp = 7]) count s with [sp = 4] s with [sp = 4] [set sp 35]] ; otherwise randomly remove as many smaller prey individuals as there were small predators minus the abundance of the larger prey

set s s with [(sp = 1 and a < 1) or (sp = 2 and a < 9) or (sp = 3 and a < 1) or (sp = 4 and a < 1)] ; update the patch set of small epifaunal individuals

set g1 count patches with [ sp = 1 ] ; give the small infaunal groups abundance variables values equal to the number of patches occupied by each group

set g2 count patches with [ sp = 2 ]

set g3 count patches with [ sp = 3 ]

set g4 count patches with [ sp = 4 ]

set g5 count patches with [ sp = 5 ] / 9 ; give the intermediate infaunal groups abundance variables values equal to the number of patches occupied by each group divided by the individually occupied number of patches

set g6 count patches with [ sp = 6 ] / 9

set g7 count patches with [ sp = 7 ] / 9

set g8 count patches with [ sp = 8 ] / 9

set g9 count patches with [ sp = 9 ] / 9

set ba g9 ; give the infaunal basibionts abundance variable a value equal to the infaunal abundance of the basibiotic group

set g10 count patches with [ sp = 10 ] / 25 ; give the large infaunal groups abundance variables values equal to the number of patches occupied by each group divided by the individually occupied number of patches

set g11 count patches with [ sp = 11 ] / 25

set p1 round ((max list g4 g7) / 10) ; give the predatory groups abundance variables values equal to those of the most abundant of their prey groups divided by 10

set p3 round ((max list g4 g5) / 10)

set p2 round ((max list g7 g11) / 10)

set p4 round ((max list p1 p3) / 10)

set es (g8 + g10) ; give the sediment stabilizing groups abundance variable a value equal to the sum of the abundances of intermediate and large sessile stabilizers that tolerate tidal exposure

set ed (g7 + g11) ; give the sediment destabilizing groups abundance variable a value equal to the sum of the abundances of intermediate and large destabilizers that tolerate tidal exposure

ask patches [set pcolor sp] ; color patches according to their infaunal group occupation variable

set g12 count patches with [ ep = 13 ] ; give the small epifaunal groups abundance variables values equal to the number of patches occupied by each group

set g13 count patches with [ ep = 14 ]

set g14 count patches with [ ep = 15 ]

set g9 g9 + count patches with [ ep = 16 ] ; give the basibiotic group abundance variable a value equal to the sum of the abundances of the infaunal and epifaunal individuals of the group

set g15 count patches with [ ep = 17 ] / 9 ; give the intermediate epifaunal groups abundance variables values equal to the number of patches occupied by each group divided by the individually occupied number of patches

set g16 count patches with [ ep = 18 ] / 9

ask patches with [ ep > 0 ] [ set pcolor ep ] ; color patches occupied by epifaunal individuals according to their epifaunal group occupation variable

output-type "g " output-type g1 output-type " " output-type g2 output-type " " output-type g3 output-type " " output-type g4 output-type " " output-type g5 output-type " " output-type g6 output-type " " output-type g7 output-type " " output-type g8 output-type " " output-type g9 output-type " " output-type g10 output-type " " output-type g11 output-type " " output-type g12 output-type " " output-type g13 output-type " " output-type g14 output-type " " output-type g15 output-type " " output-type g16 output-type " " output-type p1 output-type " " output-type p2 output-type " " output-type p3 output-type " " output-print p4 ; print out the values of infaunal/epifaunal and predatory groups abundance

set n length filter [? > 0] (list g1 g2 g3 g4 g5 g6 g7 g8 g9 g10 g11 g12 g13 g14 g15 g16 p1 p2 p3 p4) ; give the variable of number of functional groups in the system a value equal to the number of groups with abundances larger than 0

end

GRAPHICS-WINDOW

501

10

995

525

60

60

4.0

1

4

1

1

1

0

1

1

1

-60

60

-60

60

1

1

1

ticks

30.0

BUTTON

1062

10

1135

43

NIL

setup

NIL

1

T

OBSERVER

NIL

NIL

NIL

NIL

1

PLOT

37

274

484

537

Evolution of functional group abundance

Ticks

Abundance

0.0

0.0

0.0

0.0

true

true

"" ""

PENS

"FG1" 1.0 0 -2674135 true "" "plot g1"

"FG2" 1.0 0 -955883 true "" "plot g2"

"FG3" 1.0 0 -1184463 true "" "plot g3"

"FG4" 1.0 0 -10899396 true "" "plot g4"

"FG5" 1.0 0 -13840069 true "" "plot g5"

"FG6" 1.0 0 -11221820 true "" "plot g6"

"FG7" 1.0 0 -13791810 true "" "plot g7"

"FG8" 1.0 0 -8630108 true "" "plot g8"

"FG9" 1.0 0 -6459832 true "" "plot g9"

"FG10" 1.0 0 -5825686 true "" "plot g10"

"FG11" 1.0 0 -2064490 true "" "plot g11"

MONITOR

1033

452

1156

501

Number of groups

n

0

1

12

PLOT

37

10

484

273

Functional group abundance

Functional group

Abundance

0.0

20.0

0.0

10.0

true

false

"" ""

PENS

"5" 1.0 1 -16777216 true "" "plotxy 4 g5"

"6" 1.0 1 -16777216 true "" "plotxy 5 g6"

"7" 1.0 1 -16777216 true "" "plotxy 6 g7"

"8" 1.0 1 -16777216 true "" "plotxy 7 g8"

"9" 1.0 1 -16777216 true "" "plotxy 8 g9"

"10" 1.0 1 -16777216 true "" "plotxy 9 g10"

"1" 1.0 1 -16777216 true "" "plotxy 0 g1"

"2" 1.0 1 -16777216 true "" "plotxy 1 g2"

"3" 1.0 1 -16777216 true "" "plotxy 2 g3"

"4" 1.0 1 -16777216 true "" "plotxy 3 g4"

"pen-10" 1.0 1 -16777216 true "" "plotxy 16 p1"

"pen-11" 1.0 1 -16777216 true "" "plotxy 17 p2"

"pen-12" 1.0 1 -16777216 true "" "plotxy 18 p3"

"pen-13" 1.0 1 -16777216 true "" "plotxy 19 p4"

"pen-14" 1.0 1 -16777216 true "" "plotxy 10 g11"

"pen-15" 1.0 1 -16777216 true "" "plotxy 11 g12"

"pen-16" 1.0 1 -16777216 true "" "plotxy 12 g13"

"pen-17" 1.0 1 -16777216 true "" "plotxy 13 g14"

"pen-18" 1.0 1 -16777216 true "" "plotxy 14 g15"

"pen-19" 1.0 1 -16777216 true "" "plotxy 15 g16"

BUTTON

1068

66

1131

99

go

go

T

1

T

OBSERVER

NIL

NIL

NIL

NIL

1

BUTTON

1042

120

1159

153

make-movie

make-movie

NIL

1

T

OBSERVER

NIL

NIL

NIL

NIL

1

MONITOR

1033

281

1189

330

stabilizer abundance

es

0

1

12

MONITOR

1033

338

1189

387

destabilizer abundance

ed

0

1

12

BUTTON

1060

173

1138

206

export

export

NIL

1

T

OBSERVER

NIL

NIL

NIL

NIL

1

BUTTON

1061

226

1139

259

import

import

NIL

1

T

OBSERVER

NIL

NIL

NIL

NIL

1

PLOT

37

538

484

801

Evolution of predatory group abundance

Ticks

Abundance

0.0

0.0

0.0

0.0

true

true

"" ""

PENS

"FGP1" 1.0 0 -2674135 true "" "plot p1"

"FGP2" 1.0 0 -1184463 true "" "plot p2"

"FGP3" 1.0 0 -13840069 true "" "plot p3"

"FGP4" 1.0 0 -13345367 true "" "plot p4"

OUTPUT

501

550

1189

801

12

MONITOR

1033

395

1189

444

basibiont abundance

ba

0

1

12

**S.4.3 Coarse-scale model**

extensions [ ls gis ] ; load LevelSpace (ls) and GIS (gis) extensions

globals [ Rance ] ; define Rance GIS dataset

patches-own [ sediment sys sp p1 p2 p3 p4 g1 g2 g3 g4 g5 g6 g7 g8 g9 g10 g11 g12 g13 g14 g15 g16 fg1 fg2 fg3 fg4 fg5 fg6 fg7 fg8 fg9 fg10 fg11 fg12 fg13 fg14 fg15 fg16 ba ] ; define patch variables of sediment type (sediment), tidal zone (sys), number of functional groups (sp), abundances of 4 predatory groups (p1-p4), abundances of 16 infaunal/epifaunal groups (g1-g16), interim abundances of 16 infaunal/epifaunal groups (fg1-fg16), abundance of infaunal individuals of basibiotic group (ba)

to setup ; initialize the model

ca ; clear all model entities and output

ls:reset ; close down all child models

set Rance gis:load-dataset <give path and name of ".shp" file> ; load Rance GIS dataset

gis:apply-coverage Rance "LEG_ORIG" sediment ; copy sediment values from Rance GIS dataset to patch variable of sediment type

display-sediment ; color Rance patches according to their sediment type

display-system ; assign Rance patches to the subtidal or intertidal zone and color them accordingly

ls:load-gui-model <give path and name of ".nlogo" file for the subtidal fine-scale model> ; load subtidal IBM

ask patches with [ sys = 1 ] [ ; ask patches of the subtidal zone to

let pxy (word pxcor "-" pycor) ; take a name according to their x-y coordinates

ls:ask 0 "setup" ; ask the subtidal IBM to initialize

(ls:ask 0 "set xy ?" pxy) ; ask the subtidal IBM to take their name

ls:ask 0 "export" ; ask the subtidal IBM to export all entities and output to an external file with this name

ifelse "es" ls:of 0 > "ed" ls:of 0 ; take a color according to the dominance in the subtidal IBM of

[ set pcolor cyan ] ; sediment stabilizers

[ set pcolor pink ] ; or destabilizers

set g1 "g1" ls:of 0 ; give the variables of group abundances and number of functional groups the values of the respective variables of the subtidal IBM

set g2 "g2" ls:of 0

set g3 "g3" ls:of 0

set g4 "g4" ls:of 0

set g5 "g5" ls:of 0

set g6 "g6" ls:of 0

set g7 "g7" ls:of 0

set g8 "g8" ls:of 0

set g9 "g9" ls:of 0

set ba "ba" ls:of 0

set g10 "g10" ls:of 0

set g11 "g11" ls:of 0

set g12 "g12" ls:of 0

set g13 "g13" ls:of 0

set g14 "g14" ls:of 0

set g15 "g15" ls:of 0

set g16 "g16" ls:of 0

set p1 "p1" ls:of 0

set p2 "p2" ls:of 0

set p3 "p3" ls:of 0

set p4 "p4" ls:of 0

set sp "n" ls:of 0

output-type sys output-type " " output-type pxcor output-type " " output-type pycor output-type " " output-type g1 output-type " " output-type g2 output-type " " output-type g3 output-type " " output-type g4 output-type " " output-type g5 output-type " " output-type g6 output-type " " output-type g7 output-type " " output-type g8 output-type " " output-type g9 output-type " " output-type g10 output-type " " output-type g11 output-type " " output-type g12 output-type " " output-type g13 output-type " " output-type g14 output-type " " output-type g15 output-type " " output-type g16 output-type " " output-type p1 output-type " " output-type p2 output-type " " output-type p3 output-type " " output-type p4 output-type " " output-print ba ; print out the values of tidal zone, x-y coordinates and group abundances variables

]

ls:load-gui-model <give path and name of ".nlogo" file for the intertidal fine-scale model> ; load intertidal IBM

ask patches with [ sys = 2 ] [ ; ask patches of the intertidal zone to

let pxy (word pxcor "-" pycor) ; take a name according to their x-y coordinates

ls:ask 1 "setup" ; ask the intertidal IBM to initialize

(ls:ask 1 "set xy ?" pxy) ; ask the intertidal IBM to take their name

ls:ask 1 "export" ; ask the intertidal IBM to export all entities and output to an external file with this name

ifelse "es" ls:of 1 > "ed" ls:of 1 ; take a color according to the dominance in the intertidal IBM of

[ set pcolor sky ] ; sediment stabilizers

[ set pcolor magenta ] ; or destabilizers

set g1 "g1" ls:of 1 ; give the variables of group abundances and number of functional groups the values of the respective variables of the intertidal IBM

set g2 "g2" ls:of 1

set g3 "g3" ls:of 1

set g4 "g4" ls:of 1

set g5 "g5" ls:of 1

set g6 "g6" ls:of 1

set g7 "g7" ls:of 1

set g8 "g8" ls:of 1

set g9 "g9" ls:of 1

set ba "ba" ls:of 1

set g10 "g10" ls:of 1

set g11 "g11" ls:of 1

set g12 "g12" ls:of 1

set g13 "g13" ls:of 1

set g14 "g14" ls:of 1

set g15 "g15" ls:of 1

set g16 "g16" ls:of 1

set p1 "p1" ls:of 1

set p2 "p2" ls:of 1

set p3 "p3" ls:of 1

set p4 "p4" ls:of 1

set sp "n" ls:of 1

output-type sys output-type " " output-type pxcor output-type " " output-type pycor output-type " " output-type g1 output-type " " output-type g2 output-type " " output-type g3 output-type " " output-type g4 output-type " " output-type g5 output-type " " output-type g6 output-type " " output-type g7 output-type " " output-type g8 output-type " " output-type g9 output-type " " output-type g10 output-type " " output-type g11 output-type " " output-type g12 output-type " " output-type g13 output-type " " output-type g14 output-type " " output-type g15 output-type " " output-type g16 output-type " " output-type p1 output-type " " output-type p2 output-type " " output-type p3 output-type " " output-type p4 output-type " " output-print ba ; print out the values of tidal zone, x-y coordinates and group abundances variables

]

reset-ticks ; set tick counter to zero, set up and update all plots

end

to go ; move the model one step forward

set-current-plot "Subtidal functional group abundance" ; clear the plot of subtidal groups abundances

clear-plot

set-current-plot "Intertidal functional group abundance" ; clear the plot of intertidal groups abundances

clear-plot

tick ; advance the tick counter by one and update all plots

if ticks > 9 [ stop ] ; stop the model after initialization plus 9 time steps

ask patches with [ sys = 1 ] [ ; ask patches of the subtidal zone to

let pxy (word pxcor "-" pycor) ; take a name according to their x-y coordinates

let f1 median [g1] of patches in-radius 1.9 with [ pcolor > 0 ] ; define the infaunal/epifaunal groups contribution to the respective IBM spawner pool as the median abundance of each group within the patch and its eight immediate neighbors that are part of the system

let f2 median [g2] of patches in-radius 1.9 with [ pcolor > 0 ]

let f3 median [g3] of patches in-radius 1.9 with [ pcolor > 0 ]

let f4 median [g4] of patches in-radius 1.9 with [ pcolor > 0 ]

let f5 median [g5] of patches in-radius 1.9 with [ pcolor > 0 ]

let f6 median [g6] of patches in-radius 1.9 with [ pcolor > 0 ]

let f7 median [g7] of patches in-radius 1.9 with [ pcolor > 0 ]

let f8 median [g8] of patches in-radius 1.9 with [ pcolor > 0 ]

let f9 median [g9] of patches in-radius 1.9 with [ pcolor > 0 ]

let f10 median [g10] of patches in-radius 1.9 with [ pcolor > 0 ]

let f11 median [g11] of patches in-radius 1.9 with [ pcolor > 0 ]

let f12 median [g12] of patches in-radius 1.9 with [ pcolor > 0 ]

let f13 median [g13] of patches in-radius 1.9 with [ pcolor > 0 ]

let f14 median [g14] of patches in-radius 1.9 with [ pcolor > 0 ]

let f15 median [g15] of patches in-radius 1.9 with [ pcolor > 0 ]

let f16 median [g16] of patches in-radius 1.9 with [ pcolor > 0 ]

(ls:ask 0 "set xy ?" pxy) ; ask the subtidal IBM to take their name

ls:ask 0 "import" ; ask the subtidal IBM to import all entities and output from an external file with this name

(ls:ask 0 "set fg1 ?1 set fg2 ?2 set fg3 ?3 set fg4 ?4 set fg5 ?5 set fg6 ?6 set fg7 ?7 set fg8 ?8 set fg9 ?9 set fg10 ?10 set fg11 ?11 set fg12 ?12 set fg13 ?13 set fg14 ?14 set fg15 ?15 set fg16 ?16" f1 f2 f3 f4 f5 f6 f7 f8 f9 f10 f11 f12 f13 f14 f15 f16) ; give the values of the variables of the infaunal/epifaunal groups contribution to the respective spawner pool to the respective variables of the subtidal IBM

ls:ask 0 "go" ; ask the subtidal IBM to move one step forward

ls:ask 0 "export" ; ask the subtidal IBM to export all entities and output to an external file with this name

ifelse "es" ls:of 0 > "ed" ls:of 0 ; take a color according to the dominance in the subtidal IBM of

[ set pcolor yellow ] ; sediment stabilizers

[ set pcolor red ] ; or destabilizers

set sp "n" ls:of 0 ; give the variables of number of functional groups and interim infaunal/epifaunal and predatory group abundances the values of the respective variables of the subtidal IBM

set fg1 "g1" ls:of 0

set fg2 "g2" ls:of 0

set fg3 "g3" ls:of 0

set fg4 "g4" ls:of 0

set fg5 "g5" ls:of 0

set fg6 "g6" ls:of 0

set fg7 "g7" ls:of 0

set fg8 "g8" ls:of 0

set fg9 "g9" ls:of 0

set fg10 "g10" ls:of 0

set fg11 "g11" ls:of 0

set fg12 "g12" ls:of 0

set fg13 "g13" ls:of 0

set fg14 "g14" ls:of 0

set fg15 "g15" ls:of 0

set fg16 "g16" ls:of 0

set p1 "p1" ls:of 0

set p2 "p2" ls:of 0

set p3 "p3" ls:of 0

set p4 "p4" ls:of 0

output-type sys output-type " " output-type pxcor output-type " " output-type pycor output-type " " output-type fg1 output-type " " output-type fg2 output-type " " output-type fg3 output-type " " output-type fg4 output-type " " output-type fg5 output-type " " output-type fg6 output-type " " output-type fg7 output-type " " output-type fg8 output-type " " output-type fg9 output-type " " output-type fg10 output-type " " output-type fg11 output-type " " output-type fg12 output-type " " output-type fg13 output-type " " output-type fg14 output-type " " output-type fg15 output-type " " output-type fg16 output-type " " output-type p1 output-type " " output-type p2 output-type " " output-type p3 output-type " " output-type p4 output-type " " output-print ba ; print out the values of tidal zone, x-y coordinates and group abundances variables

]

ask patches with [ sys = 1 ] [ ; ask patches of the subtidal zone to give the infaunal/epifaunal group abundances variables the values of the interim infaunal/epifaunal group abundances variables

set g1 fg1

set g2 fg2

set g3 fg3

set g4 fg4

set g5 fg5

set g6 fg6

set g7 fg7

set g8 fg8

set g9 fg9

set g10 fg10

set g11 fg11

set g12 fg12

set g13 fg13

set g14 fg14

set g15 fg15

set g16 fg16

]

ask patches with [ sys = 2 ] [ ; ask patches of the intertidal zone to

let pxy (word pxcor "-" pycor) ; take a name according to their x-y coordinates

let f1 median [g1] of patches in-radius 1.9 with [ pcolor > 0 ] ; define the infaunal/epifaunal groups contribution to the respective IBM spawner pool as the median abundance of each group within the patch and its eight immediate neighbors that are part of the system

let f2 median [g2] of patches in-radius 1.9 with [ pcolor > 0 ]

let f3 median [g3] of patches in-radius 1.9 with [ pcolor > 0 ]

let f4 median [g4] of patches in-radius 1.9 with [ pcolor > 0 ]

let f5 median [g5] of patches in-radius 1.9 with [ pcolor > 0 ]

let f6 median [g6] of patches in-radius 1.9 with [ pcolor > 0 ]

let f7 median [g7] of patches in-radius 1.9 with [ pcolor > 0 ]

let f8 median [g8] of patches in-radius 1.9 with [ pcolor > 0 ]

let f9 median [g9] of patches in-radius 1.9 with [ pcolor > 0 ]

let f10 median [g10] of patches in-radius 1.9 with [ pcolor > 0 ]

let f11 median [g11] of patches in-radius 1.9 with [ pcolor > 0 ]

let f12 median [g12] of patches in-radius 1.9 with [ pcolor > 0 ]

let f13 median [g13] of patches in-radius 1.9 with [ pcolor > 0 ]

let f14 median [g14] of patches in-radius 1.9 with [ pcolor > 0 ]

let f15 median [g15] of patches in-radius 1.9 with [ pcolor > 0 ]

let f16 median [g16] of patches in-radius 1.9 with [ pcolor > 0 ]

(ls:ask 1 "set xy ?" pxy) ; ask the intertidal IBM to take their name

ls:ask 1 "import" ; ask the intertidal IBM to import all entities and output from an external file with this name

(ls:ask 1 "set fg1 ?1 set fg2 ?2 set fg3 ?3 set fg4 ?4 set fg5 ?5 set fg6 ?6 set fg7 ?7 set fg8 ?8 set fg9 ?9 set fg10 ?10 set fg11 ?11 set fg12 ?12 set fg13 ?13 set fg14 ?14 set fg15 ?15 set fg16 ?16" f1 f2 f3 f4 f5 f6 f7 f8 f9 f10 f11 f12 f13 f14 f15 f16) ; give the values of the variables of the infaunal/epifaunal groups contribution to the respective spawner pool to the respective variables of the intertidal IBM

ls:ask 1 "go" ; ask the intertidal IBM to move one step forward

ls:ask 1 "export" ; ask the intertidal IBM to export all entities and output to an external file with this name

ifelse "es" ls:of 1 > "ed" ls:of 1 ; take a color according to the dominance in the intertidal IBM of

[ set pcolor green ] ; sediment stabilizers

[ set pcolor orange ] ; or destabilizers

set sp "n" ls:of 1 ; give the variables of number of functional groups and interim infaunal/epifaunal and predatory group abundances the values of the respective variables of the intertidal IBM

set fg1 "g1" ls:of 1

set fg2 "g2" ls:of 1

set fg3 "g3" ls:of 1

set fg4 "g4" ls:of 1

set fg5 "g5" ls:of 1

set fg6 "g6" ls:of 1

set fg7 "g7" ls:of 1

set fg8 "g8" ls:of 1

set fg9 "g9" ls:of 1

set fg10 "g10" ls:of 1

set fg11 "g11" ls:of 1

set fg12 "g12" ls:of 1

set fg13 "g13" ls:of 1

set fg14 "g14" ls:of 1

set fg15 "g15" ls:of 1

set fg16 "g16" ls:of 1

set p1 "p1" ls:of 1

set p2 "p2" ls:of 1

set p3 "p3" ls:of 1

set p4 "p4" ls:of 1

output-type sys output-type " " output-type pxcor output-type " " output-type pycor output-type " " output-type fg1 output-type " " output-type fg2 output-type " " output-type fg3 output-type " " output-type fg4 output-type " " output-type fg5 output-type " " output-type fg6 output-type " " output-type fg7 output-type " " output-type fg8 output-type " " output-type fg9 output-type " " output-type fg10 output-type " " output-type fg11 output-type " " output-type fg12 output-type " " output-type fg13 output-type " " output-type fg14 output-type " " output-type fg15 output-type " " output-type fg16 output-type " " output-type p1 output-type " " output-type p2 output-type " " output-type p3 output-type " " output-type p4 output-type " " output-print ba ; print out the values of tidal zone, x-y coordinates and group abundances variables

]

ask patches with [ sys = 2 ] [ ; ask patches of the intertidal zone to give the infaunal/epifaunal group abundances variables the values of the interim infaunal/epifaunal group abundances variables

set g1 fg1

set g2 fg2

set g3 fg3

set g4 fg4

set g5 fg5

set g6 fg6

set g7 fg7

set g8 fg8

set g9 fg9

set g10 fg10

set g11 fg11

set g12 fg12

set g13 fg13

set g14 fg14

set g15 fg15

set g16 fg16

]

end

to display-sediment ; color Rance patches according to their sediment type and all other patches black

ask patches

[ ifelse ( is-string? sediment )

[ set pcolor read-from-string sediment ]

[ set pcolor black ] ]

end

to display-system ; assign Rance patches to one of two tidal zones according to their sediment type, subtidal (gravel, coarse sand, intermediate/coarse sand, fine/intermediate sand, muddy sand, sandy mud) or intertidal (silty mud, mud, pure mud, salt marshes) and color them accordingly

ask patches with [pcolor > 10 and pcolor < 90] [set pcolor 15 set sys 1]

ask patches with [pcolor > 90 and pcolor < 140] [set pcolor 45 set sys 2]

end

GRAPHICS-WINDOW

462

10

968

537

-1

-1

16.0

1

16

1

1

1

0

0

0

1

0

30

0

30

0

0

1

ticks

30.0

BUTTON

159

10

232

43

setup

setup

NIL

1

T

OBSERVER

NIL

NIL

NIL

NIL

1

BUTTON

80

51

232

84

display sediment

display-sediment

NIL

1

T

OBSERVER

NIL

NIL

NIL

NIL

1

BUTTON

246

51

390

84

display system

display-system

NIL

1

T

OBSERVER

NIL

NIL

NIL

NIL

1

BUTTON

246

10

309

43

go

go

T

1

T

OBSERVER

NIL

NIL

NIL

NIL

1

MONITOR

132

151

232

200

sub stab

count patches with [ pcolor = 45 ]

0

1

12

MONITOR

25

151

123

200

sub dest

count patches with [ pcolor = 15 ]

0

1

12

PLOT

2

203

460

546

Number of functional groups

Ticks

Functional groups

0.0

10.0

0.0

10.0

true

true

"" ""

PENS

"max" 1.0 0 -2674135 true "" "plot max [sp] of patches with [ pcolor > 0 ]"

"median" 1.0 0 -1184463 true "" "plot median [sp] of patches with [ pcolor > 0 ]"

"min" 1.0 0 -13345367 true "" "plot min [sp] of patches with [ pcolor > 0 ]"

OUTPUT

2

553

977

806

12

PLOT

983

10

1892

408

Subtidal functional group abundance

Functional group

Abundance

0.0

20.0

0.0

10.0

true

false

"" ""

PENS

"default" 1.0 1 -16777216 true "" "plotxy 0 mean [g1] of patches with [sys = 1]"

"pen-1" 1.0 1 -16777216 true "" "plotxy 1 mean [g2] of patches with [sys = 1]"

"pen-2" 1.0 1 -16777216 true "" "plotxy 2 mean [g3] of patches with [sys = 1]"

"pen-3" 1.0 1 -16777216 true "" "plotxy 3 mean [g4] of patches with [sys = 1]"

"pen-4" 1.0 1 -16777216 true "" "plotxy 4 mean [g5] of patches with [sys = 1]"

"pen-5" 1.0 1 -16777216 true "" "plotxy 5 mean [g6] of patches with [sys = 1]"

"pen-6" 1.0 1 -16777216 true "" "plotxy 6 mean [g7] of patches with [sys = 1]"

"pen-7" 1.0 1 -16449023 true "" "plotxy 7 mean [g8] of patches with [sys = 1]"

"pen-8" 1.0 1 -16777216 true "" "plotxy 8 mean [g9] of patches with [sys = 1]"

"pen-9" 1.0 1 -16777216 true "" "plotxy 9 mean [g10] of patches with [sys = 1]"

"pen-10" 1.0 1 -16777216 true "" "plotxy 16 mean [p1] of patches with [sys = 1]"

"pen-11" 1.0 1 -16777216 true "" "plotxy 17 mean [p2] of patches with [sys = 1]"

"pen-12" 1.0 1 -16777216 true "" "plotxy 18 mean [p3] of patches with [sys = 1]"

"pen-13" 1.0 1 -16777216 true "" "plotxy 19 mean [p4] of patches with [sys = 1]"

"pen-14" 1.0 1 -16777216 true "" "plotxy 10 mean [g11] of patches with [sys = 1]"

"pen-15" 1.0 1 -16777216 true "" "plotxy 11 mean [g12] of patches with [sys = 1]"

"pen-16" 1.0 1 -16777216 true "" "plotxy 12 mean [g13] of patches with [sys = 1]"

"pen-17" 1.0 1 -16777216 true "" "plotxy 13 mean [g14] of patches with [sys = 1]"

"pen-18" 1.0 1 -16777216 true "" "plotxy 14 mean [g15] of patches with [sys = 1]"

"pen-19" 1.0 1 -16777216 true "" "plotxy 15 mean [g16] of patches with [sys = 1]"

PLOT

983

411

1892

809

Intertidal functional group abundance

Functional group

Abundance

0.0

20.0

0.0

10.0

true

false

"" ""

PENS

"default" 1.0 1 -16777216 true "" "plotxy 0 mean [g1] of patches with [sys = 2]"

"pen-1" 1.0 1 -16777216 true "" "plotxy 1 mean [g2] of patches with [sys = 2]"

"pen-2" 1.0 1 -16777216 true "" "plotxy 2 mean [g3] of patches with [sys = 2]"

"pen-3" 1.0 1 -16777216 true "" "plotxy 3 mean [g4] of patches with [sys = 2]"

"pen-4" 1.0 1 -16777216 true "" "plotxy 4 mean [g5] of patches with [sys = 2]"

"pen-5" 1.0 1 -16777216 true "" "plotxy 5 mean [g6] of patches with [sys = 2]"

"pen-6" 1.0 1 -16777216 true "" "plotxy 6 mean [g7] of patches with [sys = 2]"

"pen-7" 1.0 1 -16777216 true "" "plotxy 7 mean [g8] of patches with [sys = 2]"

"pen-8" 1.0 1 -16777216 true "" "plotxy 8 mean [g9] of patches with [sys = 2]"

"pen-9" 1.0 1 -16777216 true "" "plotxy 9 mean [g10] of patches with [sys = 2]"

"pen-10" 1.0 1 -16777216 true "" "plotxy 16 mean [p1] of patches with [sys = 2]"

"pen-11" 1.0 1 -16777216 true "" "plotxy 17 mean [p2] of patches with [sys = 2]"

"pen-12" 1.0 1 -16777216 true "" "plotxy 18 mean [p3] of patches with [sys = 2]"

"pen-13" 1.0 1 -16777216 true "" "plotxy 19 mean [p4] of patches with [sys = 2]"

"pen-14" 1.0 1 -16777216 true "" "plotxy 10 mean [g11] of patches with [sys = 2]"

"pen-15" 1.0 1 -16777216 true "" "plotxy 11 mean [g12] of patches with [sys = 2]"

"pen-16" 1.0 1 -16777216 true "" "plotxy 12 mean [g13] of patches with [sys = 2]"

"pen-17" 1.0 1 -16777216 true "" "plotxy 13 mean [g14] of patches with [sys = 2]"

"pen-18" 1.0 1 -16777216 true "" "plotxy 14 mean [g15] of patches with [sys = 2]"

"pen-19" 1.0 1 -16777216 true "" "plotxy 15 mean [g16] of patches with [sys = 2]"

MONITOR

246

151

343

200

inter dest

count patches with [ pcolor = 25 ]

0

1

12

MONITOR

352

151

449

200

inter stab

count patches with [ pcolor = 55 ]

0

1

12

MONITOR

25

93

123

142

in sub dest

count patches with [ pcolor = 135 ]

0

1

12

MONITOR

132

93

232

142

in sub stab

count patches with [ pcolor = 85 ]

0

1

12

MONITOR

246

93

343

142

in inter dest

count patches with [ pcolor = 125 ]

0

1

12

MONITOR

352

93

449

142

in inter stab

count patches with [ pcolor = 95 ]

0

1

12

**S.5 Model output of the benchmark simulation**


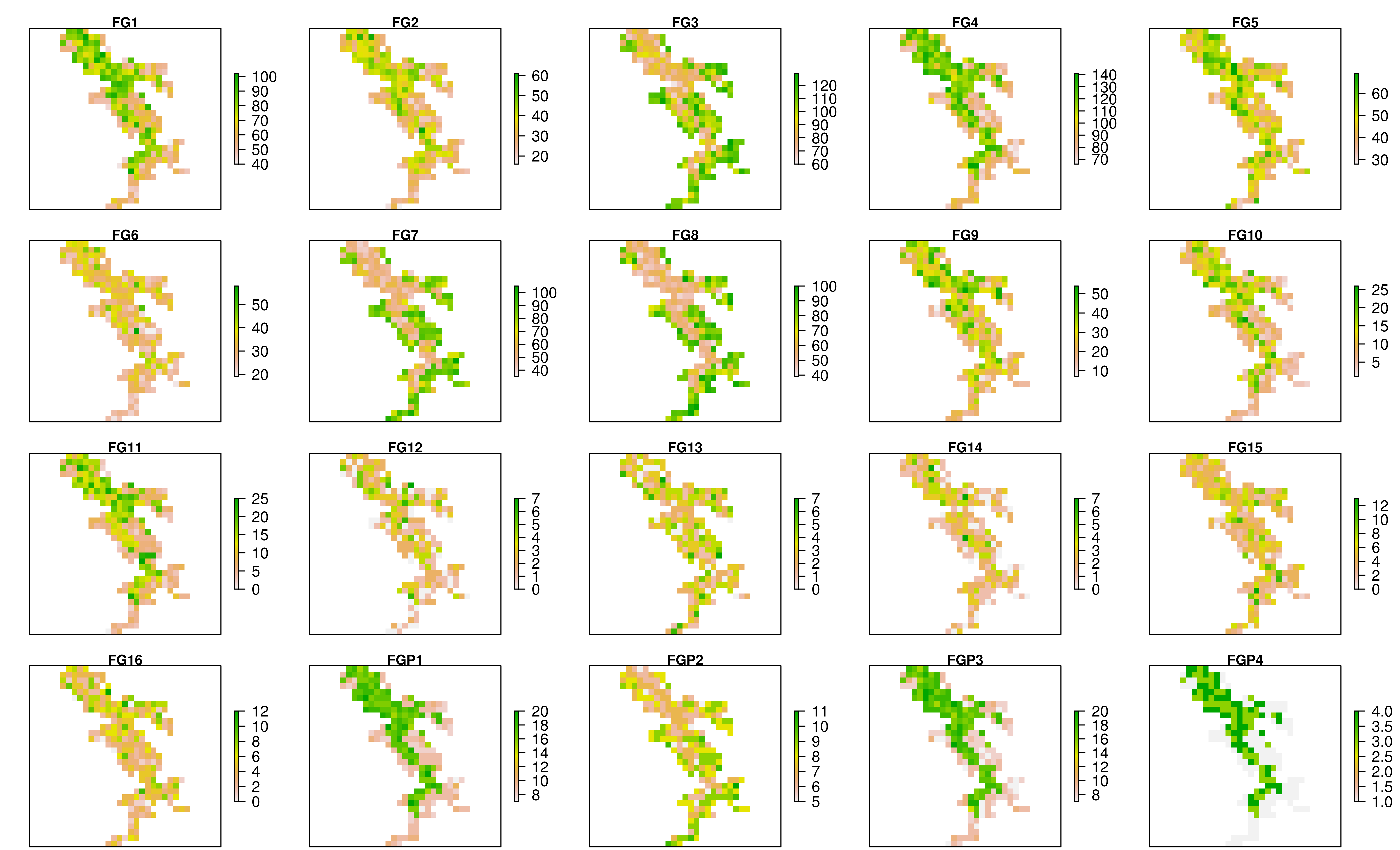


**Fig. A** Functional group abundances in the 1^st^ year of the benchmark simulation


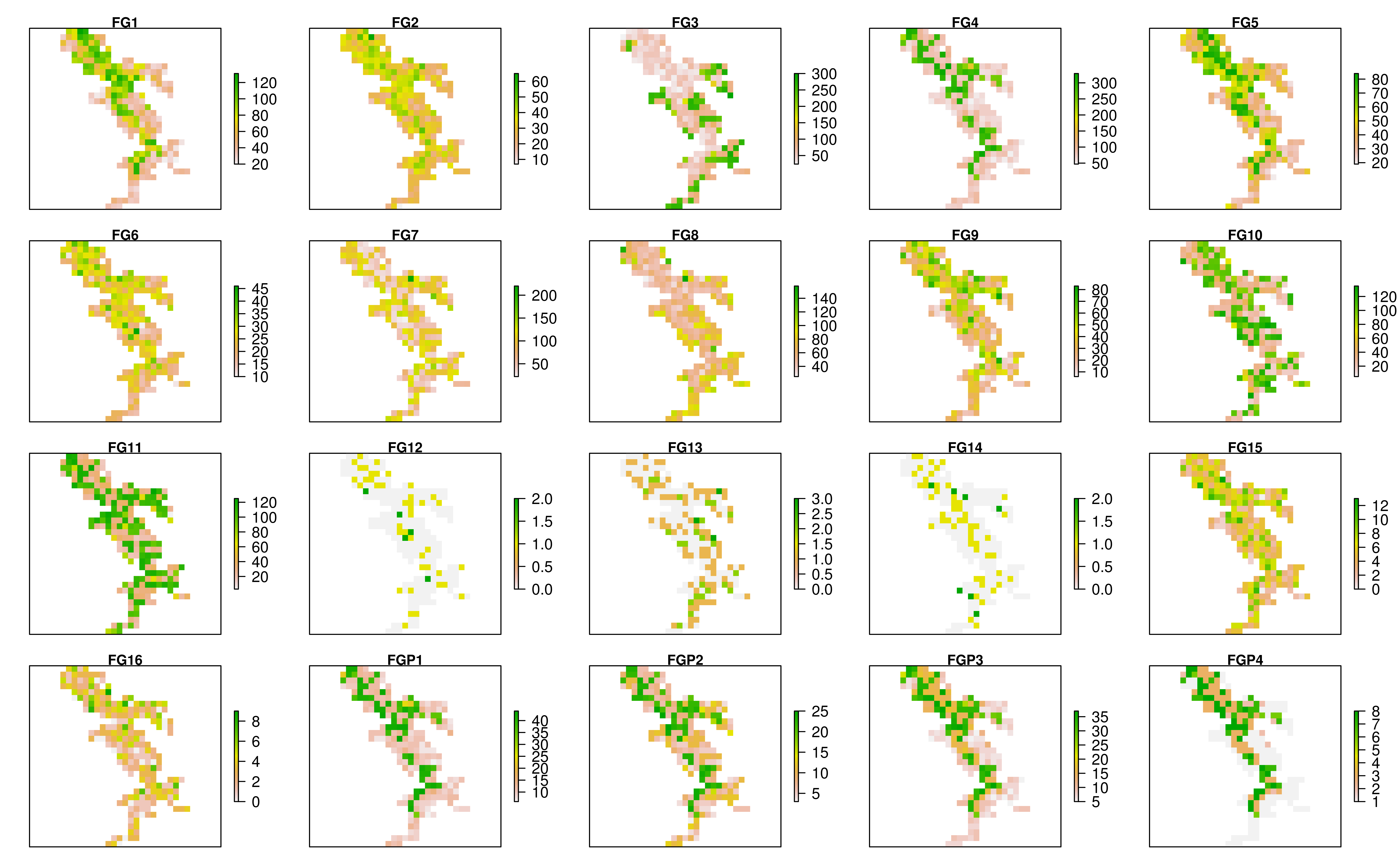


**Fig. B** Functional group abundances in the 2^nd^ year of the benchmark simulation


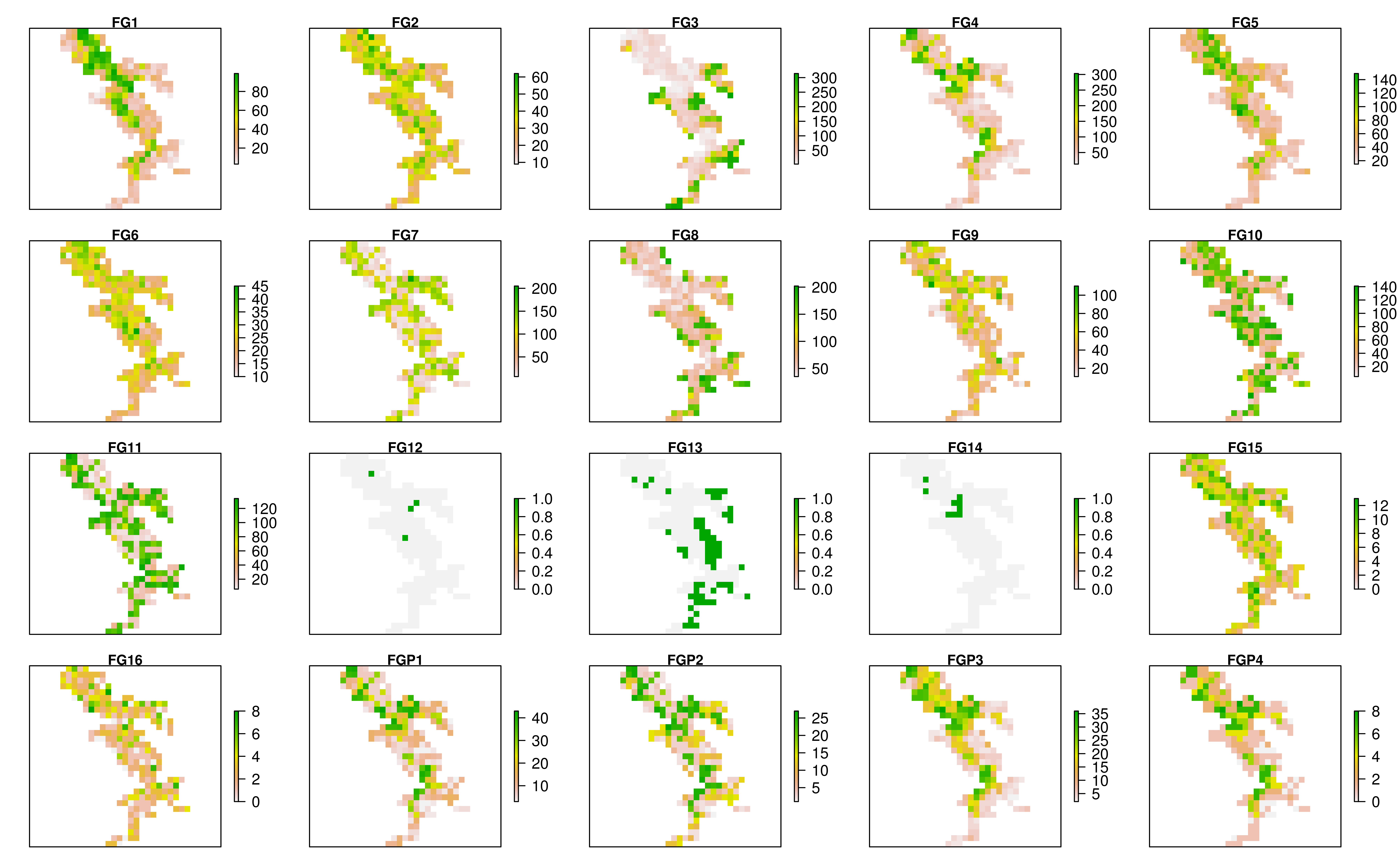


**Fig. C** Functional group abundances in the 3^rd^ year of the benchmark simulation


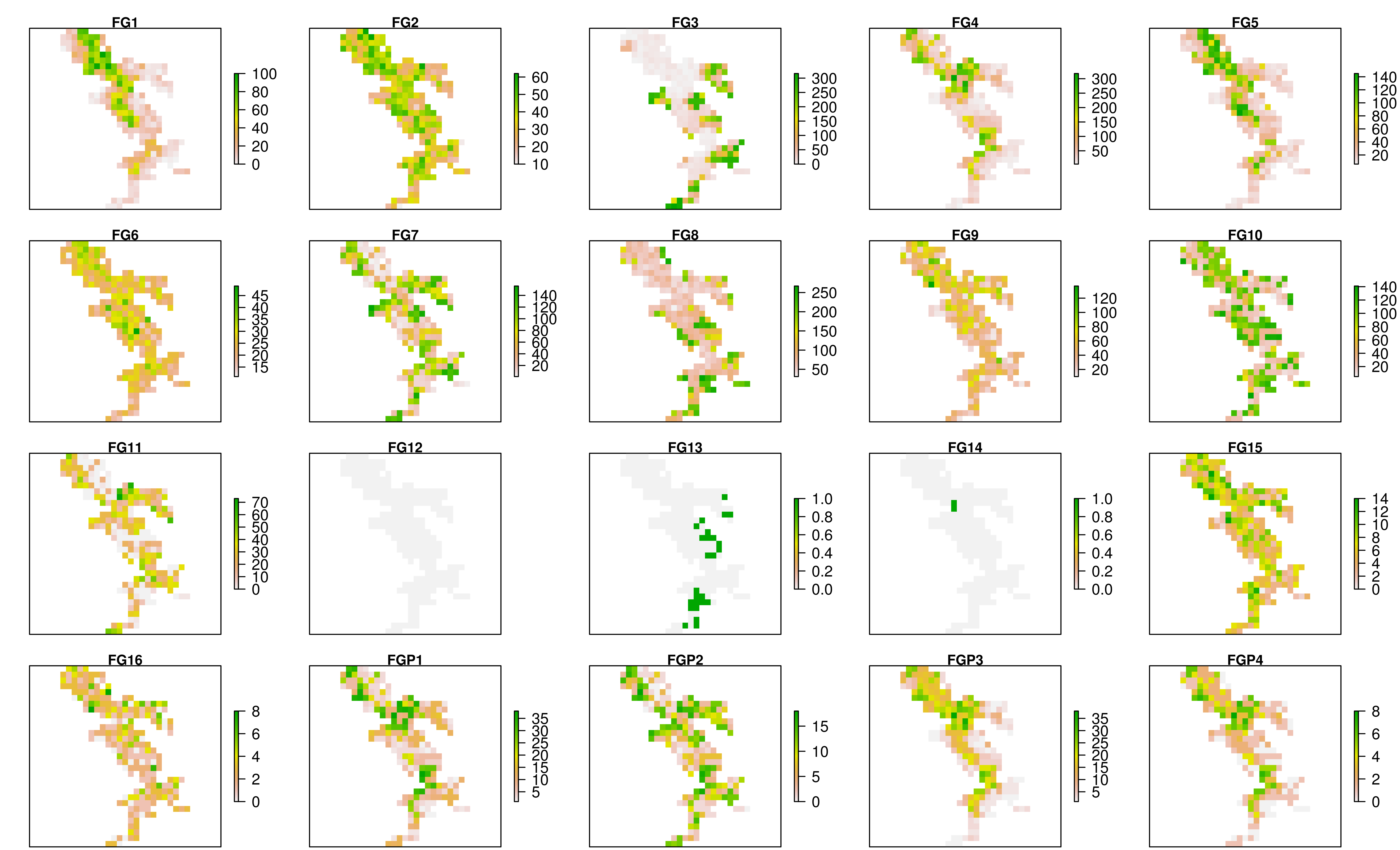


**Fig. D** Functional group abundances in the 4^th^ year of the benchmark simulation


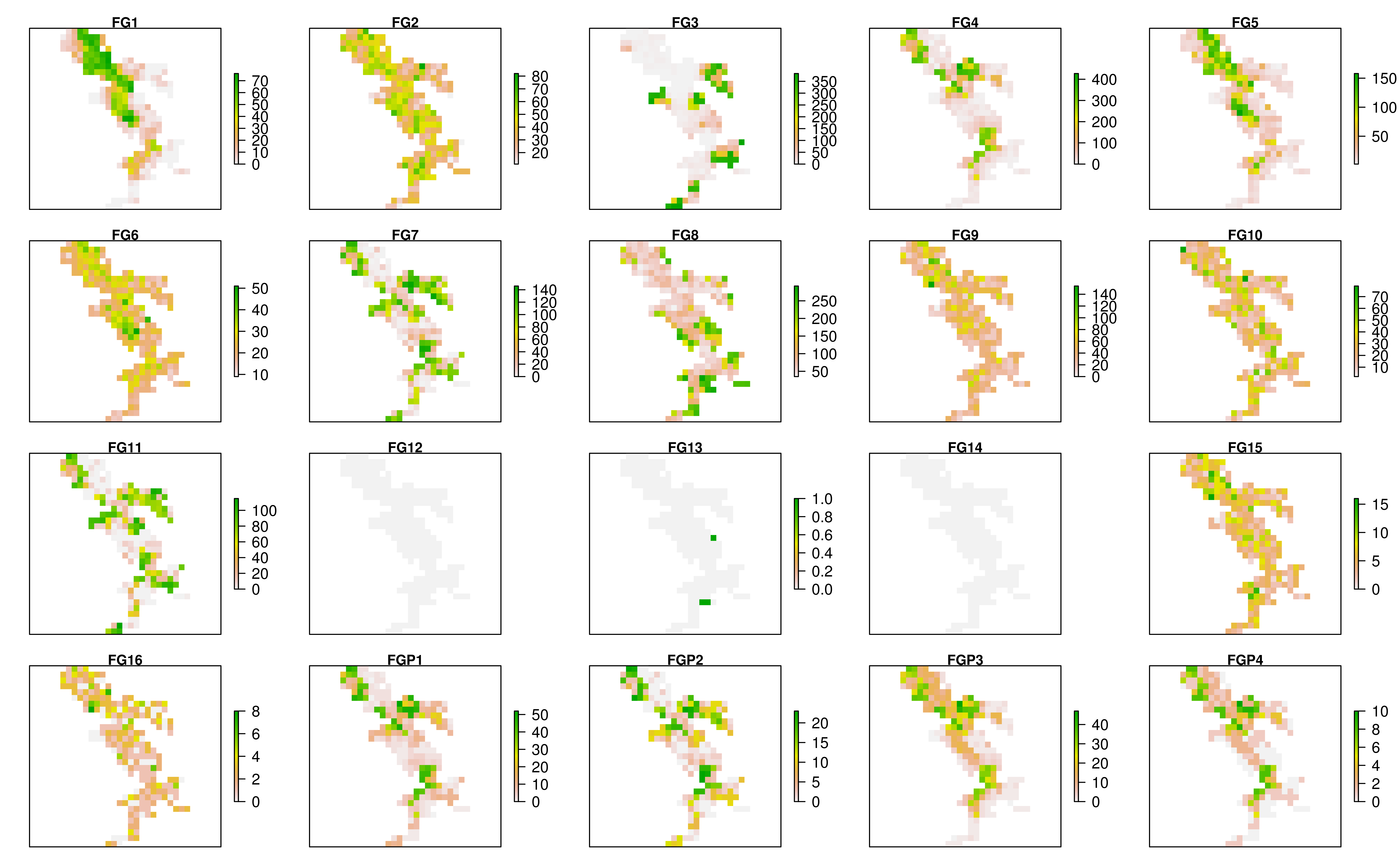


**Fig. E** Functional group abundances in the 5^th^ year of the benchmark simulation


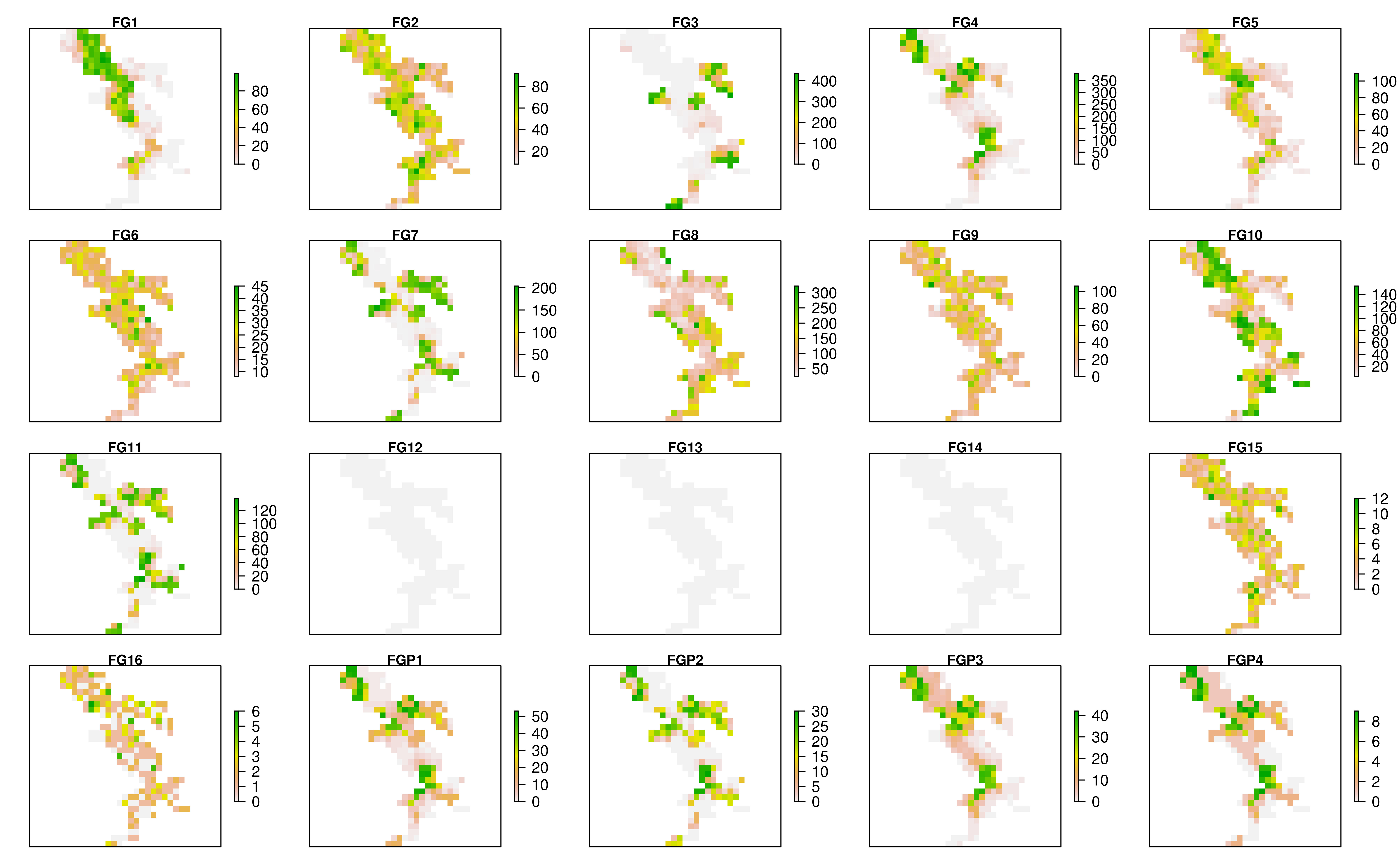


**Fig. F** Functional group abundances in the 6^th^ year of the benchmark simulation


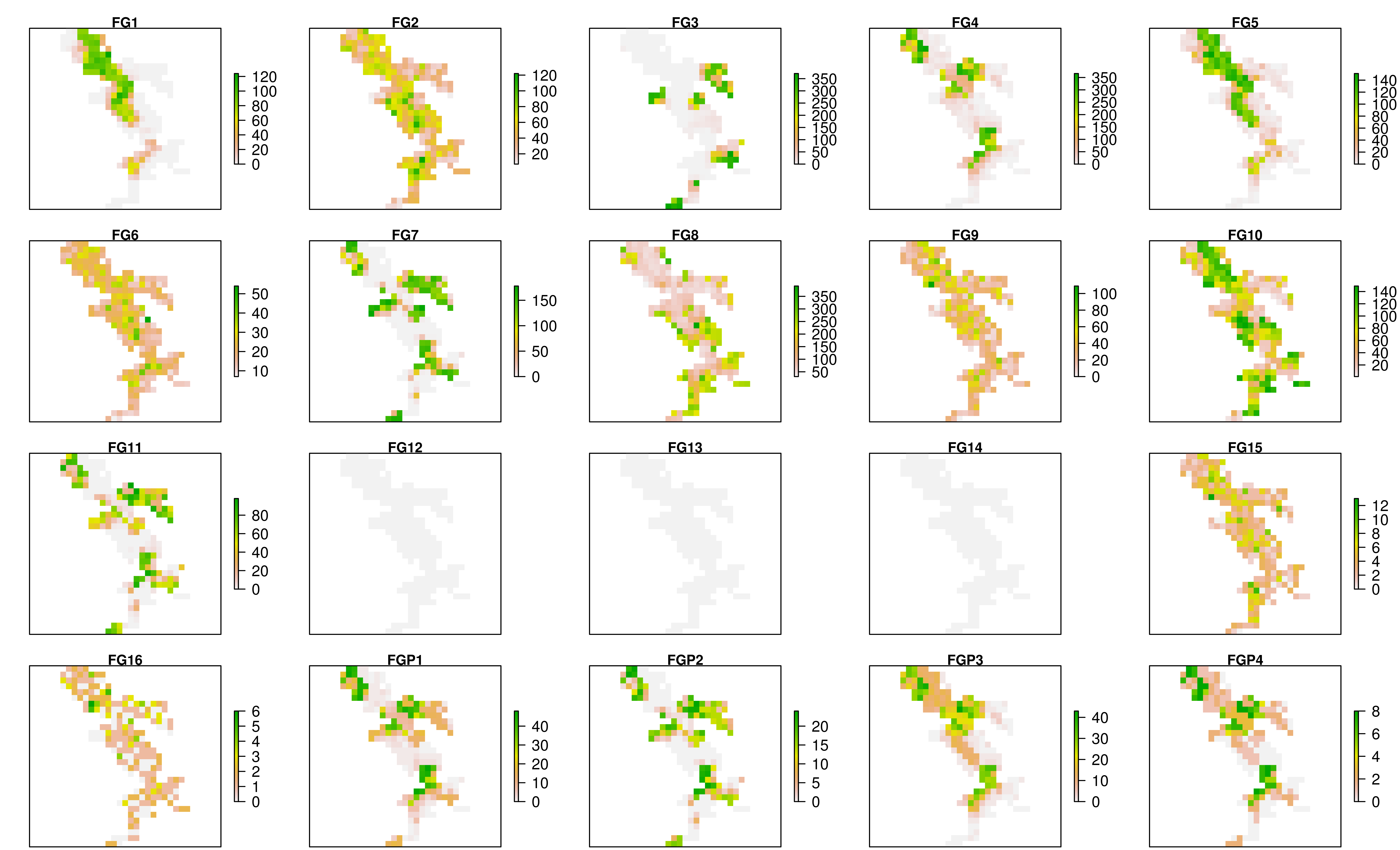


**Fig. G** Functional group abundances in the 7^th^ year of the benchmark simulation


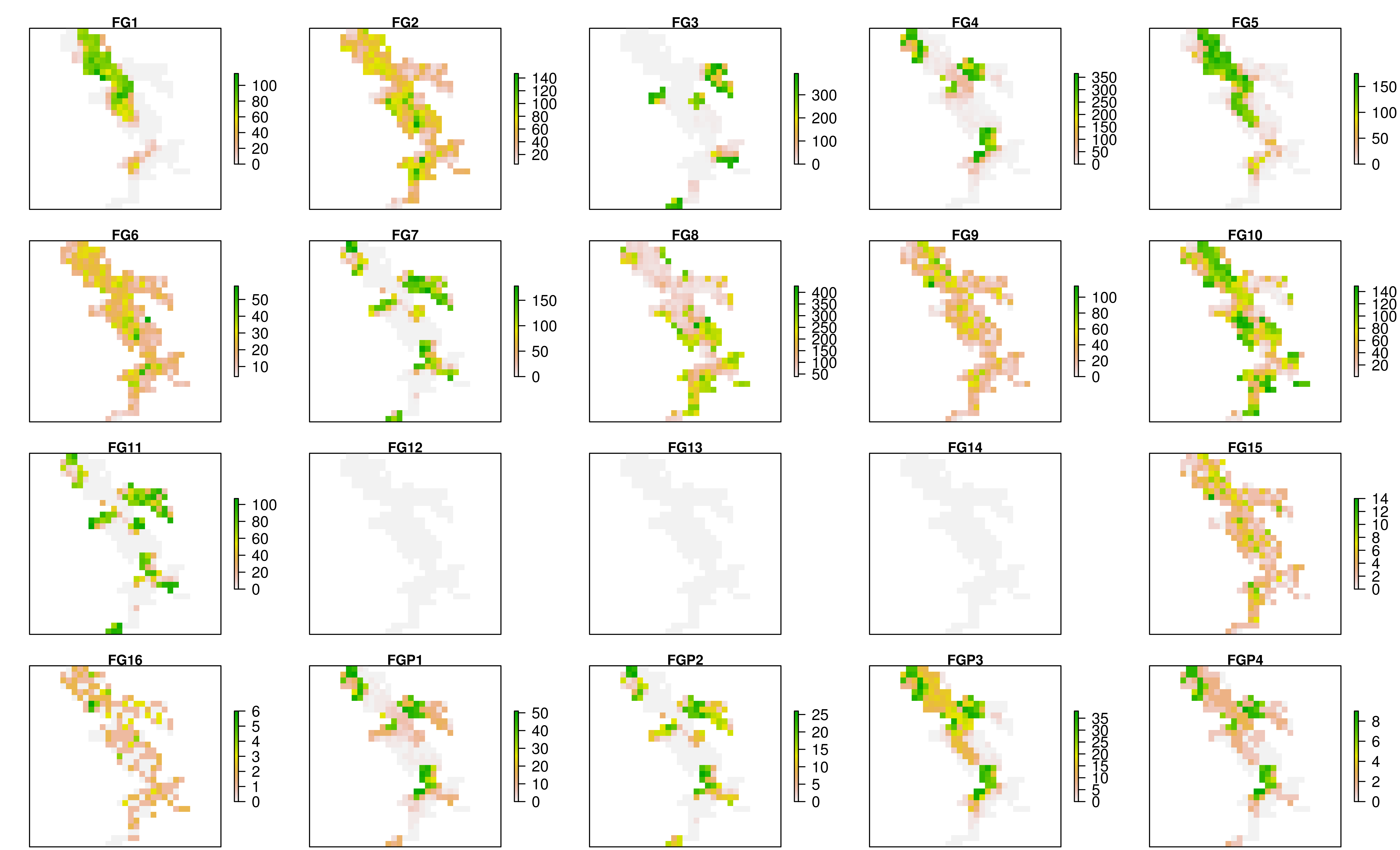


**Fig. H** Functional group abundances in the 8^th^ year of the benchmark simulation


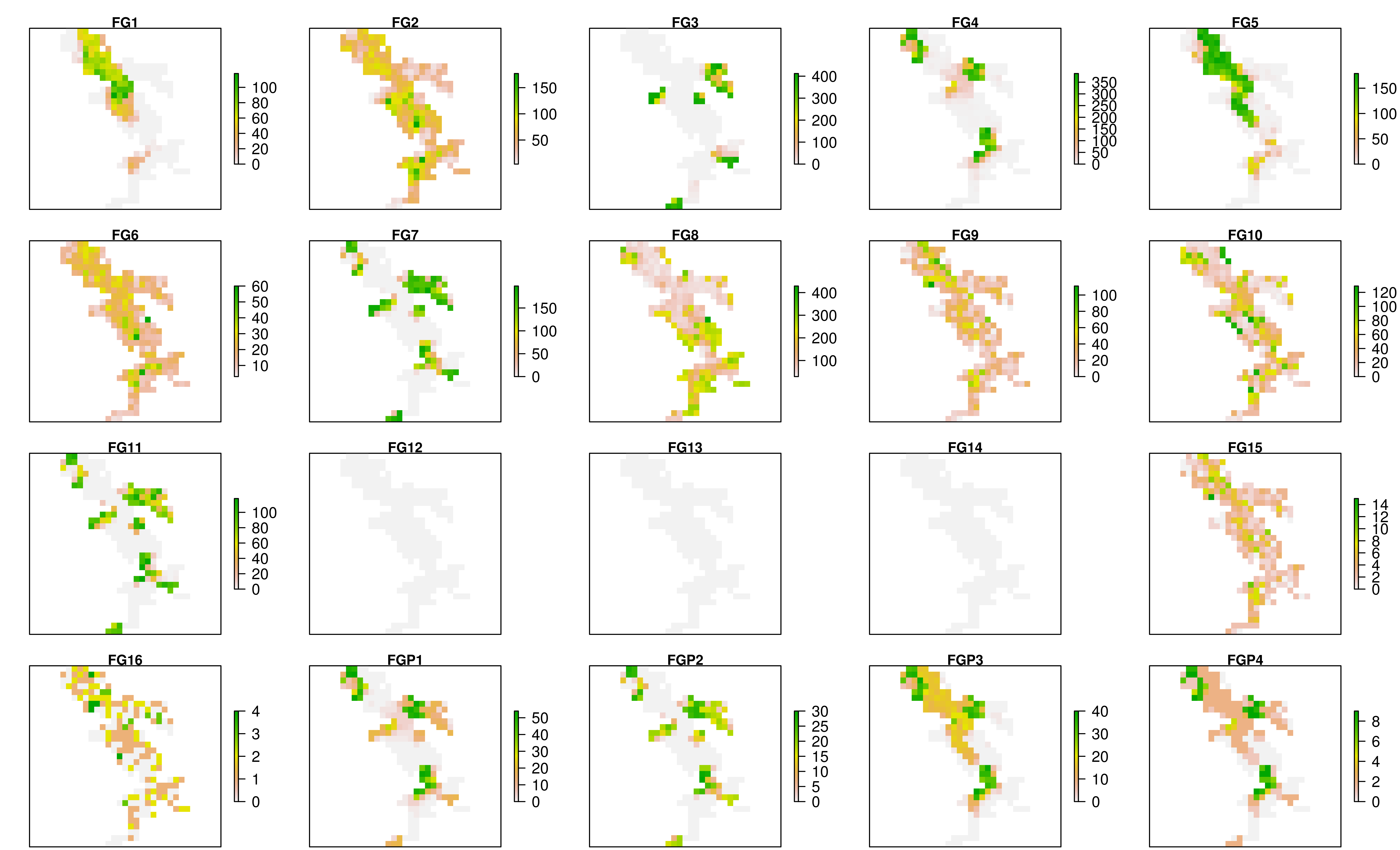


**Fig. I** Functional group abundances in the 9^th^ year of the benchmark simulation


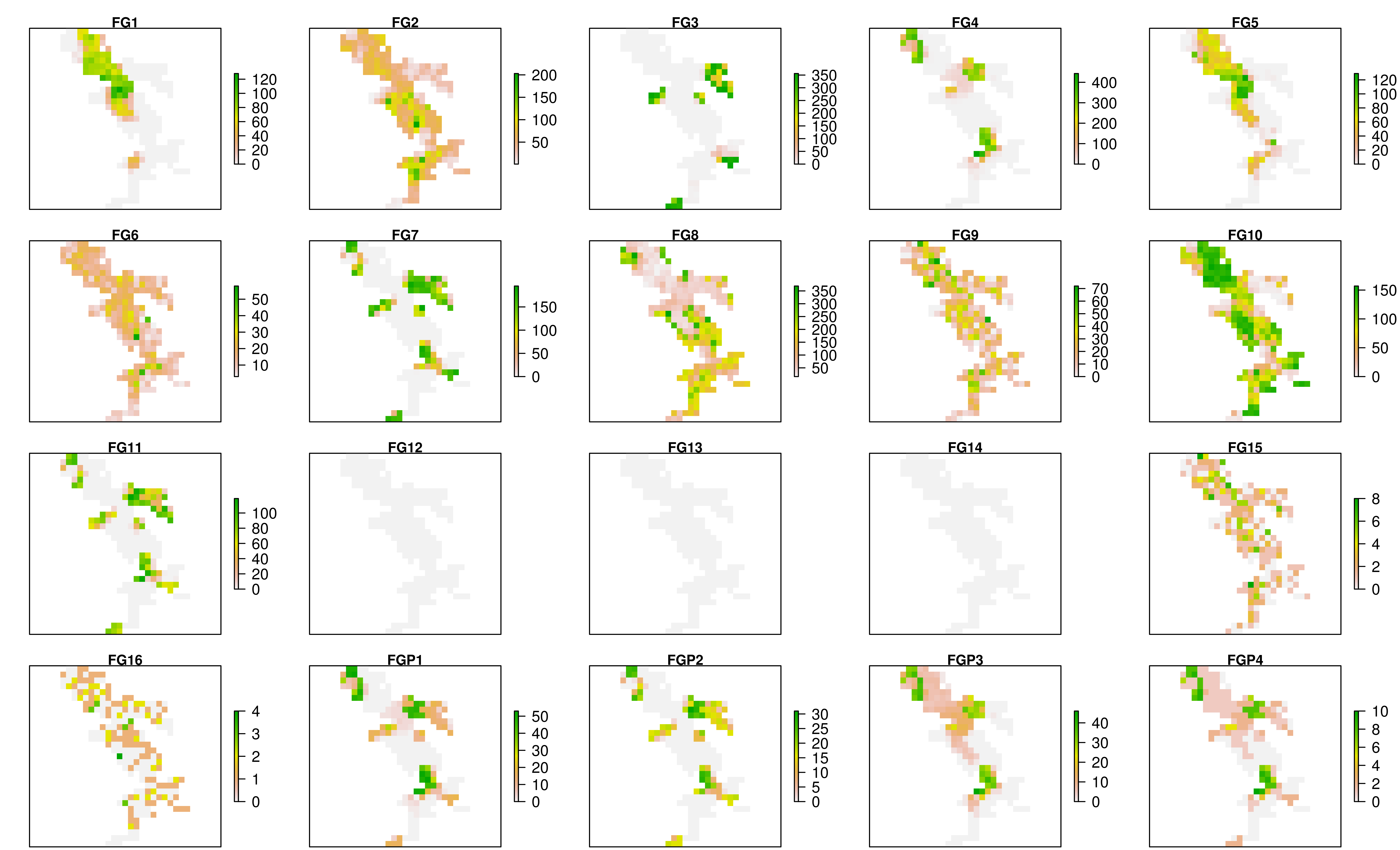


**Fig. J** Functional group abundances in the 10^th^ year of the benchmark simulation

**References**

Alexandridis, N., Bacher, C., Desroy, N., Jean, F., 2017a. Building functional groups of marine benthic macroinvertebrates on the basis of general community assembly mechanisms. Journal of Sea Research 121, 59-70.

Alexandridis, N., Dambacher, J.M., Jean, F., Desroy, N., Bacher, C., 2017b. Qualitative modelling of functional relationships in marine benthic communities. Ecological Modelling 360, 300-312.

Brose, U., Jonsson, T., Berlow, E.L., Warren, P., Banasek-Richter, C., Bersier, L.-F., Blanchard, J.L., Brey, T., Carpenter, S.R., Blandenier, M.-F.C., Cushing, L., Dawah, H.A., Dell, T., Edwards, F., Harper-Smith, S., Jacob, U., Ledger, M.E., Martinez, N.D., Memmott, J., Mintenbeck, K., Pinnegar, J.K., Rall, B.C., Rayner, T.S., Reuman, D.C., Ruess, L., Ulrich, W., Williams, R.J., Woodward, G., Cohen, J.E., 2006a. Consumer–resource body-size relationships in natural food webs. Ecology 87, 2411-2417.

Brose, U., Williams, R.J., Martinez, N.D., 2006b. Allometric scaling enhances stability in complex food webs. Ecology Letters 9, 1228-1236.

Cohen, J.E., Pimm, S.L., Yodzis, P., Saldaña, J., 1993. Body Sizes of Animal Predators and Animal Prey in Food Webs. Journal of Animal Ecology 62, 67-78.

Côté, J., Himmelman, J.H., Claereboudt, M.R., 1994. Separating effects of limited food and space on growth of the giant scallop Placopecten magellanicus in suspended culture. Marine Ecology Progress Series 106, 85-91.

Desroy, N., 1998. Les peuplements benthiques de substrats meubles du bassin maritime de la Rance. Évolution de la biodiversité et effets de l'activité prédatrice de *Nephtys hombergii* (Annélide Polychète). Université de Rennes 1, Rennes, France.

Gosselin, L.A., Qian, P.Y., 1997. Juvenile mortality in benthic marine invertebrates. Marine Ecology Progress Series 146, 265-282.

Hunt, H.L., Scheibling, R.E., 1997. Role of early post-settlement mortality in recruitment of benthic marine invertebrates. Marine Ecology Progress Series 155, 269-301.

Kupriyanova, E.K., Nishi, E., Ten Hove, H.A., Rzhavsky, A.V., 2001. Life-history patterns in serpulimorph polychaetes: Ecological and evolutionary perspectives (Gibson, R.B., Barnes, M., Atkinson, R.J.A., eds). Taylor & Francis Ltd, London, UK.

Meadows, P.S., Meadows, A., Murray, J.M.H., 2012. Biological modifiers of marine benthic seascapes: Their role as ecosystem engineers. Geomorphology 157–158, 31-48.

Pineda, J., Reyns, N.B., Starczak, V.R., 2009. Complexity and simplification in understanding recruitment in benthic populations. Population Ecology 51, 17-32.

Posey, M.H., 1987. Influence of relative mobilities on the composition of bentic communities. Marine Ecology Progress Series 39, 99-104.

Snelgrove, P., Butman, C., 1994. Animal-sediment relationships revisited: cause versus effect. Oceanography and Marine Biology: an Annual Review 32, 111-117.

Van Colen, C., Montserrat, F., Vincx, M., Herman, P.M.J., Ysebaert, T., Degraer, S., 2008. Macrobenthic recovery from hypoxia in an estuarine tidal mudflat. Marine Ecology Progress Series 372, 31-42.

Wahl, M., 1989. Marine epibiosis. I. Fouling and antifouling: some basic aspects. Marine Ecology Progress Series 58, 175-189.
